# *Sareomycetes*: more diverse than meets the eye

**DOI:** 10.1186/s43008-021-00056-0

**Published:** 2021-03-16

**Authors:** James K. Mitchell, Isaac Garrido-Benavent, Luis Quijada, Donald H. Pfister

**Affiliations:** 1 Farlow Reference Library and Herbarium of Cryptogamic Botany, Harvard University, 22 Divinity Avenue, Cambridge, MA 02138 USA; 2 Department of Physics, Harvard University, 17 Oxford Street, Cambridge, MA 02138 USA; 3 Institut Cavanilles de Biodiversitat i Biologia Evolutiva (ICBIBE) & Dept. Botànica i Geologia, Universitat de València, C/ Dr. Moliner 50, 46100–Burjassot, València, Spain; 4 Department of Organismic and Evolutionary Biology, Harvard University, 26 Oxford Street, Cambridge, MA 02138 USA

**Keywords:** *Atrozythia*, Cryptic diversity, Integrative taxonomy, Resinicolous fungi, *Sarea*, *Zythia*, New taxa

## Abstract

Since its resurrection, the resinicolous discomycete genus *Sarea* has been accepted as containing two species, one with black apothecia and pycnidia, and one with orange. We investigate this hypothesis using three ribosomal (nuITS, nuLSU, mtSSU) regions from and morphological examination of 70 specimens collected primarily in Europe and North America. The results of our analyses support separation of the traditional *Sarea difformis s.lat.* and *Sarea resinae s.lat.* into two distinct genera, *Sarea* and *Zythia*. *Sarea* as circumscribed is shown to conservatively comprise three phylospecies, with one corresponding to *Sarea difformis s.str.* and two, morphologically indistinguishable, corresponding to the newly combined *Sarea coeloplata*. *Zythia* is provisionally maintained as monotypic, containing only a genetically and morphologically variable *Z. resinae*. The new genus *Atrozythia* is erected for the new species *A. klamathica*. *Arthrographis lignicola* is placed in this genus on molecular grounds, expanding the concept of *Sareomycetes* by inclusion of a previously unknown type of asexual morph. Dating analyses using additional marker regions indicate the emergence of the *Sareomycetes* was roughly concurrent with the diversification of the genus *Pinus*, suggesting that this group of fungi emerged to exploit the newly-available resinous ecological niche supplied by *Pinus* or another, extinct group of conifers. Our phylogeographic studies also permitted us to study the introductions of these fungi to areas where they are not native, including Antarctica, Cape Verde, and New Zealand and are consistent with historical hypotheses of introduction.

**Supplementary Information:**

The online version contains supplementary material available at 10.1186/s43008-021-00056-0.

## INTRODUCTION

Conifers, particularly in the families *Araucariaceae*, *Pinaceae*, and *Cupressaceae*, produce resins in their tissues (Langenheim 2003) as part of a complex defence system to protect against herbivores (Smith 1961; Rudinsky 1966; van Buijtenen and Santamour 1972), pathogenic fungi (Whitney and Denyer 1969; Gibbs 1972; Hart et al. 1975; Yamada 2001), protists (Krupa and Nylund 1972; Bunny and Tippett 1988), and bacteria (Hemingway and Greaves 1973; Hartmann et al. 1981). To protect against fungi, resins have the potential to act in several different manners. First, they present a physical barrier to penetration by fungal hyphae (Verrall 1938; Shain 1971; Rishbeth 1972; Prior 1976). When soft, resin can flow, trapping fungal hyphae and spores; when hard, the resin is difficult to penetrate. Furthermore, the components of the resin can inhibit the growth of fungi, acting as a chemical barrier (Cobb Jr et al. 1968; Hintikka 1970; De Groot 1972; Fries 1973; Väisälä 1974; Chou and Zabkiewicz 1976; Bridges 1987; Yamamoto et al. 1997). Despite this apparently inhospitable environment, a number of so-called "resinicolous" fungi have evolved to exploit this niche (Cappelletti 1924; Selva and Tuovila 2016).

The study of fungi growing on conifer resins has a long history, dating back to the fathers of mycology (Persoon 1801; Fries 1815, 1822). The first species described was *Helotium aureum*, described in 1801 by Christiaan Persoon, though he made no mention of the resinicolous habit (Seifert and Carpenter 1987). Thus, the first author to describe fungi dwelling on resin was Elias Fries, who described three such fungi in 1815. *Sphaeria resinae* and *Lecidea resinae* were described as sharing the same habitat and easily confused; these were later determined to represent the asexual and sexual morphs of the same fungus, currently known as *Sarea resinae* (Ayers 1941; Hawksworth and Sherwood 1981). The third species, *Racodium resinae*, described from *Picea* resin, is a synnematous hyphomycete now called *Sorocybe resinae* (Seifert et al. 2007). These three Friesian species were followed by *Cytospora resinae*, described by Ehrenberg (1818); this was later determined to be a synonym of Fries' *Sphaeria resinae* (Fries 1823; von Thümen 1880). The last of these early species was described in 1822, again by Fries, as *Peziza difformis,* currently known as *Sarea difformis*. No additional new resinicolous taxa were noted until Arnold (1858).

The two species assigned to the genus *Sarea*, *S. resinae* and *S. difformis*, are the most commonly collected and reported of these resinicolous fungi. A search of the Global Biodiversity Information Facility (GBIF) database for *S. resinae* yielded 1261 records, and one for "*Sarea resinae*" on Google Scholar 249 results; *S. difformis* gave 519 records and 196 results, respectively. In contrast, *Sorocybe resinae* gives only 24 records and 56 results (accessed 13 July 2020). In addition to frequent reports, the two *Sarea* species have also been a subject of some interest regarding their systematic placement, which has been unclear (Reeb et al. 2004; Miadlikowska et al. 2014). A recent study resolved the uncertainty and has supported the erection of a new class in *Pezizomycotina*, *Sareomycetes* (Beimforde et al. 2020). This study, as well as a recent study that yielded 31 endolichenic isolates of *Sarea* species (Masumoto and Degawa 2019), have illustrated that both *Sarea* species are genetically diverse. This pattern is present in published sequences of both *Sarea* species deposited in public repositories. Sequence similarity and phylogenetic analyses also suggest that *Arthrographis lignicola*, though morphologically unlike *Sarea* species, is a close relative (Giraldo et al. 2014). This, combined with the wide distributions of these species, suggest a higher than known diversity, both obvious and cryptic, in *Sareomycetes*. The aim of this study is to assess this diversity.

To assess this diversity within *Sareomycetes*, an integrative taxonomic approach was employed. Fresh and fungarium specimens of orange (*Sarea resinae*) and black (*S. difformis*) species from around the world were borrowed or collected and examined morphologically. Where possible, DNA was extracted, and several regions amplified and sequenced. Two multi-locus datasets were assembled to explore species boundaries and their phylogenetic relationships and to provide further insights on the evolutionary history of *Sareomycetes* on a temporal and spatial scale.

## MATERIALS AND METHODS

### Specimens examined and microscopic examination

During the course of this study, a number of specimens of *Sarea* were collected and examined by us. The host range and distribution of these specimens was broad, with collections from the United States (California, Georgia, Maine, Massachusetts, Minnesota, New Hampshire, Rhode Island, and Vermont) made by J.K.M. and collections from Austria, Cape Verde, Spain, and Switzerland made by I.G.-B. Further specimens were collected by and lent by Tomás J. Curtis (Ohio), Alden C. Dirks (Michigan, Wisconsin), Michael Haldeman (Idaho, Washington), Jason M. Karakehian (Maine, Massachusetts, Newfoundland), Elizabeth Kneiper (Maine, Massachusetts), Jiří Malíček (Czechia), Rubén Negrín Piñero (Canary Islands), Donald H. Pfister (Dominican Republic), Michaela Schmull (New York), Judi Thomas (Missouri), Per Vetlesen (Norway), and Andrus Voitk (Newfoundland); these specimens are deposited in FH, KE, MICH, VAL, and several personal herbaria. Further specimens of *Sarea* and other critical materials from the following fungaria were studied: B, CANL, DUKE, FH, H, K, LD, MICH, NCSLG, NY, TFM, TNS, and TROM.

**Fig. 1:**
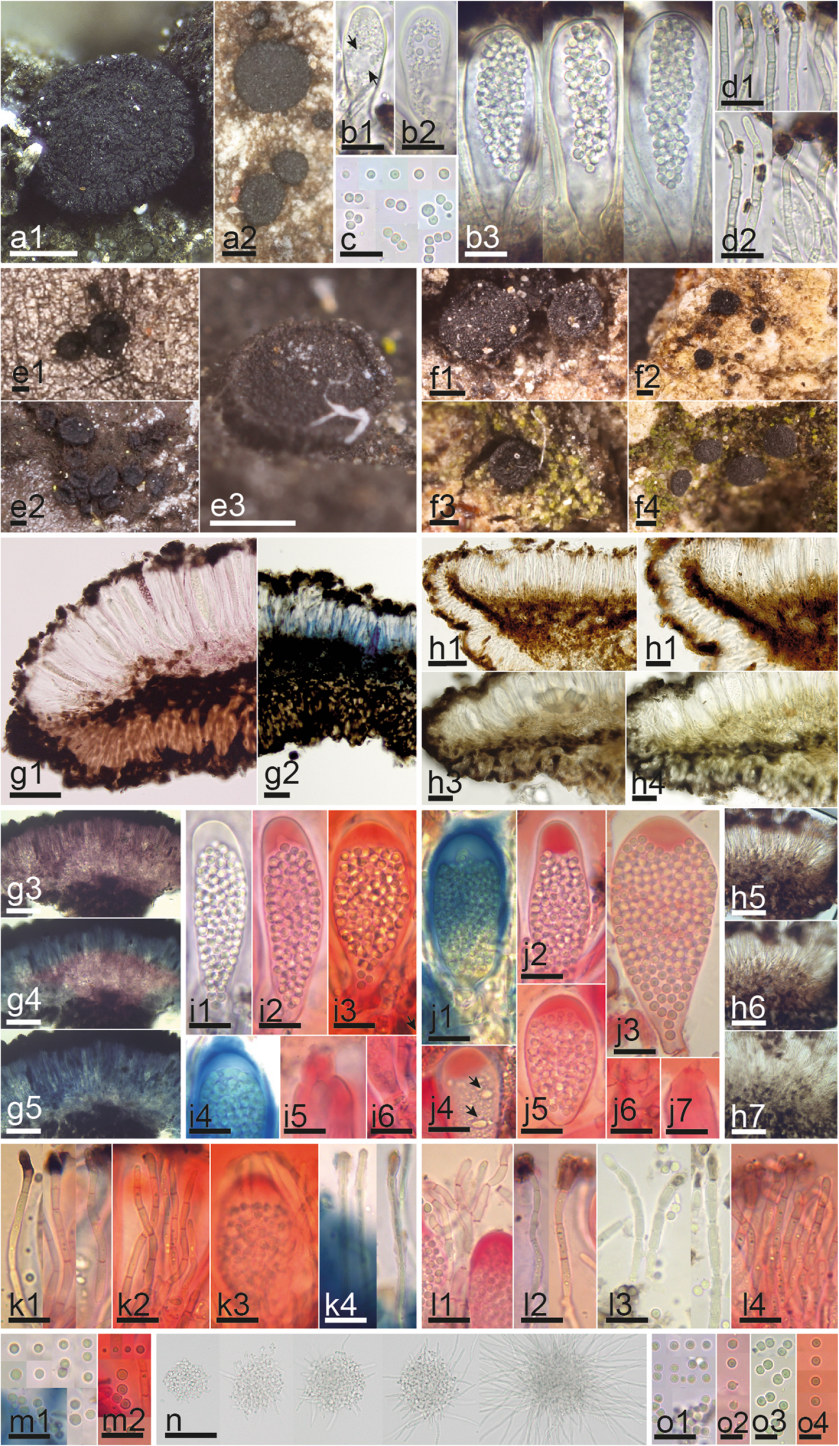
Morphological features of *Sarea* spp. **a1–d2** Fresh collection and living asci, ascospores and paraphyses of *Sarea coeloplata*. **e–o** Comparative morphology between *S. difformis* (**e, g, i, k, m, n**) and *S. coeloplata* (**f, h, j, l, o**). **e–f** Dry or rehydrated apothecia on substrate. **g–h** Median section of apothecium with ectal and medullary excipulum and changes after adding KOH. **i–j** Mature asci with ascus dehiscence and base with croziers. **k–l** Paraphyses. **m & o** Ascospores. **n** Ascospore shoot in culture and hyphal germination. Reagents: H_2_O = b1–3, c, d1–2; g1, h1, h3, n; KOH = g2–5; h2, h4–7, i1, l3, o3; KOH+CR = i2–3, i5–6, j2–7, k1–3, l1–2, l4, m2, o2, o4; KOH+MLZ = i4, j1, k4, m1, o1. Scale bars: 200 μm = a1–2, e1–3, f1–4; 50 μm = g1–5, h1, h5–7, n; 10 μm = b1–3, c, d1–2, h2–4, i1–6, j1–7, k1–4, l1–4, m1–2, o1; 10 μm = o2–4. Collections: BHI-F925 = f3, h3–7, j3, l3, o3; IGB454 = f1–2; IGB457 = j5–7, l1, o1; IGB448 = h1–2; JM0007 = e1, i1–2, i4–6, k4, m1–2; JM0009.2 = i3, k1–3; JM0010.1 = g3–5; JM0011 = f4, j1, j4, l4, o4; JM0132 = a1–2, b1–3, c, d1–2, n; JM0072.1 = j2, l2, o2; JM0074.1 = e3; JMEK = e2; PV-D836 = g2; Rehm *Ascomyceten* 577 (FH 00995483) = g1

**Fig. 2:**
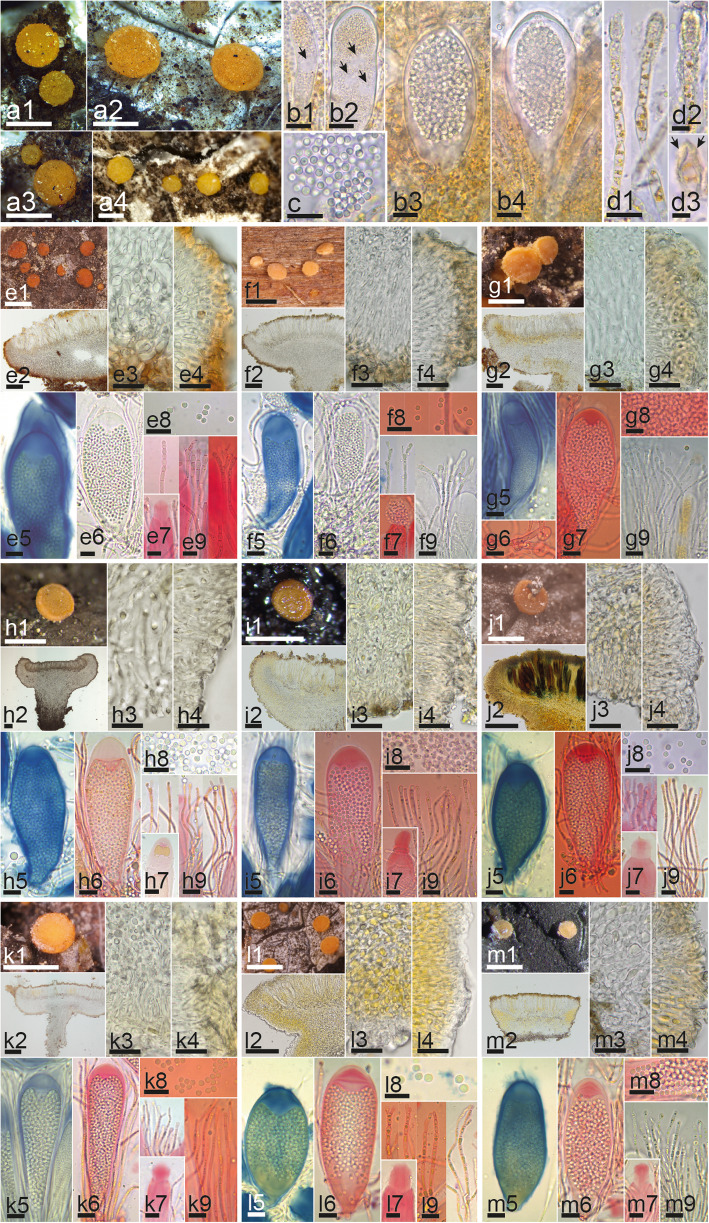
Morphological features of *Zythia resinae*. **a1–d3** Fresh collection and living asci, ascospores and paraphyses. From e to m each letter represents the morphology of one specimen for each different clade (Fig. S1): **e1–9** Clade 3, **f1–9** Clade 2, **g1–9** Clade 1, **h1–9** Clade 5, **i1–9** Clade 6, **j1–9** Clade 8, **k1–9** Clade 12, **l1–9** Clade 13, **m1–9** Clade 9. Numbers after the letter e to m indicate different morphological features: 1. Dry apothecia, 2. Median section of apothecium, 3–4. Excipular cells. 5–7. Asci, 8. Ascospores, and 9. Paraphyses. Reagents: H_2_O = b1–4, c, d1–3; KOH = e2–4, e6, e8, f2–4, f6, f9, g2–4, g9, h2–4, h8, i2–4, j3–4, j8, k3–4, l3–4, m3–4, m9; KOH+CR = e7, e9, f7–8, g6–8, h6–7, h9, i6–9, j6–7, j9, k2, k6–9, l6–7, l9, m6–8; KOH+MLZ = e5, f5, g5, h5, i5, j2, j5, k5, l2, l5, l8, m2, m5. Scale bars: 500 μm = a1–4; e1, f1, g1, h1, i1, j1, k1, l1, m1; 100 μm = e2, f2, g2, h2, i2, j2 k2, l2, m2; 20 μm = e3–4, f3–4, g3–4, h3–4, i3–4, j3–4, k3–4, l3–4, m3–4; 10 μm = b1–4, c, d1, e5–9, f5–9, g5–9, h5–9, i5–9, j5–9, k5–9, l5–9, m5–9; 5 μm = d2–3. Collections: 17121601 = m1–9; HJMS11998 = i1–9; JM0120 = h1–9; JM0014 = l1–9; JM0131 = a1–4, b1–4, c, d1–3; JM0006 = j1–9; JM0065.1 = k1–9; LD1356193 = e1–9; PV-D836-Ba = g1–9; TNS-F-41522 = f1–9

**Fig. 3:**
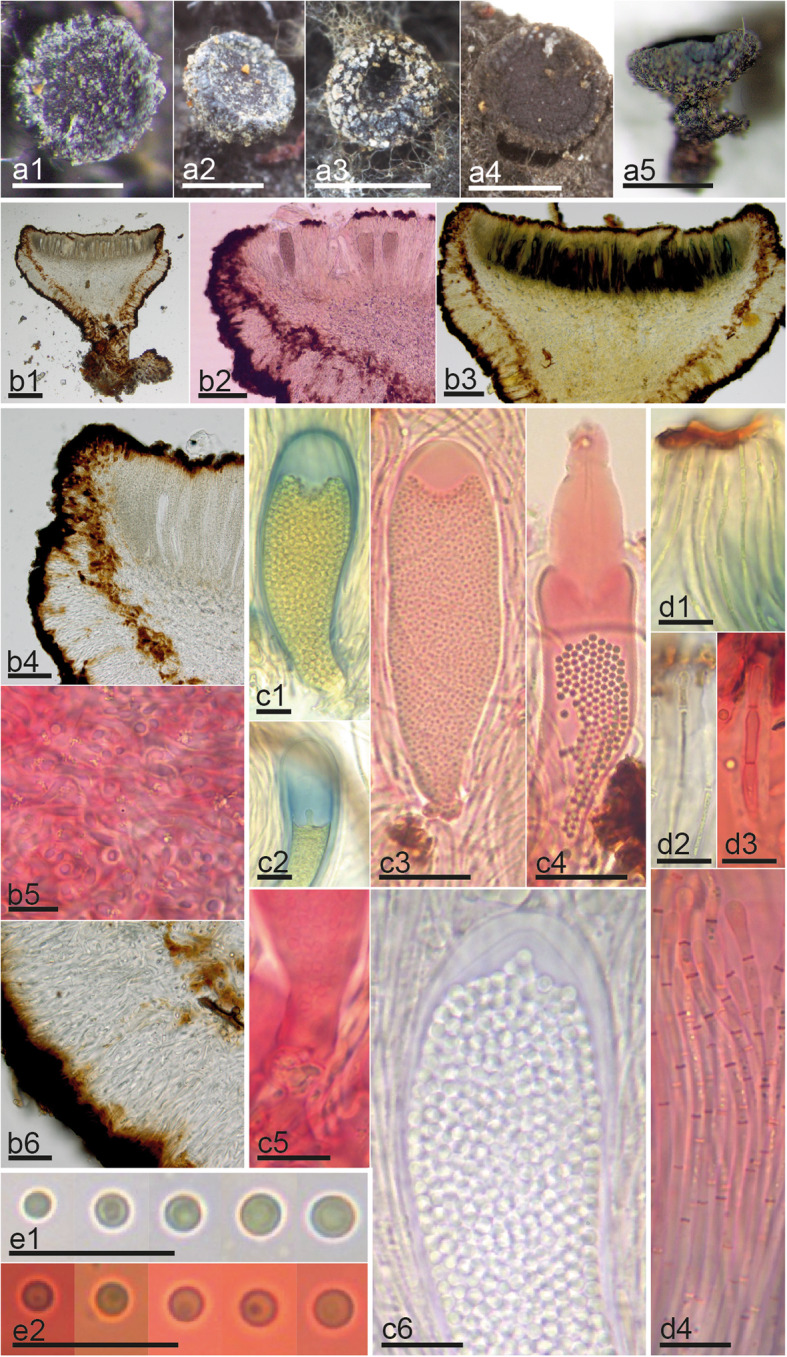
Morphological features of *Atrozythia klamathica*. **a1–5** Dry apothecia. **b1–6** Median section of apothecium with details of excipulum: b4. Ectal excipulum at margin, b5. Medullary excipulum, b6. Ectal excipulum at lower flanks. **c1–6** Morphological variation of asci: c1–2. Amyloid walls, c3. multispored mature ascus, c4. Ascus dehiscence, c5. Perforated crozier, c6. Details of ascus walls. **d1–4** Paraphyses. **e1–2** Ascospores. Reagents: H_2_O = b4, b6; KOH = b1, c6, e1; KOH+CR = b2, b5, c3–5, d1, d3–4, e2; KOH+MLZ = b3, c1–2, d2. Scale bars: 500 μm = a1–5; 200 μm = b1; 100 μm = b2–3; 50 μm = b4, b6, c3–4; 10 μm = b5, c1–2, c5–6, d1–4, e1–2. Collections: JM0068 = a1–2, a5, b1–6, c5–6, d2–4, e1; Haldeman 2748 = a3–4, c1–4, d1, e2

Microscopic examination of hymenial elements was conducted using free-hand sections cut under a dissecting microscope (Wild M5; Leica Geosystems, Heerbrugg, Switzerland) and of the excipulum using sections made on a freezing microtome. Microtome sections were prepared by stabilizing water-hydrated apothecia on a freezing stage (Physitemp BFS-MP; Physitemp Instruments, Clifton, NJ) with a diluted gum arabic solution and sectioning with a sliding microtome (Bausch & Lomb Optical, Rochester, NY) set at approximately 25 μm. The resulting sections were applied serially to a clean glass slide and allowed to adhere by drying in the remaining gum arabic. Slides were prepared under a dissecting microscope (Olympus SZX9; Olympus Corporation, Tokyo, Japan) and studied with a compound microscope (Olympus BX40; Olympus Corporation, Tokyo, Japan). Digital images were captured with an Olympus XC50 USB camera (Olympus Corporation, Tokyo, Japan). Hand sections were studied with a compound microscope (Motic B1; Motic, Hong Kong, China). Except for two fresh collections studied alive in tap water (Fig. 1, b1-d2, Fig. 2, b1-d3) and a culture studied on potato dextrose agar (PDA) (Fig. 1, n), all the other specimens (Fig. 1, g1-m2, o1-o4, Fig. 2, e2-e9, f2-f9, g2-g9, h2-h9, i2-i9, j2-j9, k2-k9, l2-l9, m2-m9, Fig. 3, b1-d4), were pre-treated in 5% KOH prior to morphological studies. Melzer’s reagent (MLZ) was used to test amyloidicity and Congo red (CR) to contrast cells walls. Images were captured with a Moticam 2500 USB camera and processed with the software Motic images Plus 2.0 (Motic, Hong Kong, China). The 95% confidence intervals of the median were calculated with SPSS 15.0 (SPSS, Chicago, IL) for each morphological feature. Measurements are given as follows: (the smallest single measurement) smallest value for percentile of 95% - Largest value for percentile of 95% (largest single measurement). Whenever possible, biometric values are based on ≥10 measurements for each character on an individual specimen.

### Culturing

Some specimens were grown in axenic culture. Cultures were generated from discharged ascospores. A living apothecium was placed oriented upward on a dab of petroleum jelly on a filter paper. This assemblage was then placed in the lid of an upside-down, sterile petri dish containing either PDA or cornmeal agar (CMA) prepared according to the manufacturer's instructions (HiMedia Laboratories, Mumbai, India). The filter paper was saturated with water, and the chamber sealed with Parafilm (Bemis Company, Neenah, WI). After incubation at room temperature for one or two days, the lid was removed and replaced with another sterile lid. The culture was then allowed to grow at 25**°**C for up to one month before sampling. Once sampled, cultures were not preserved.

### DNA extraction, PCR, and sequencing

DNA extractions were performed from axenic culture when available and from fresh or preserved apothecia or pycnidia otherwise. Fresh or plentiful dried material was extracted by grinding 1-2 apothecia, 3-4 pycnidia, or a rice grain-sized slice of a culture and employing the DNeasy Plant Mini Kit (QIAGEN, Venlo, The Netherlands) following the manufacturer's recommendations. Preserved or scanty material was extracted by grinding .25-2 apothecia or 2-3 pycnidia and employing the QIAamp DNA Micro Kit (QIAGEN), again following the manufacturer's recommendations.

Three rDNA regions were amplified: the internal transcribed spacer regions plus 5.8S gene (nuITS), the nuclear large subunit ribosomal RNA gene (nuLSU), and the mitochondrial small subunit ribosomal RNA gene (mtSSU). For older material, nuITS was obtained in two parts by employing the primer pairs ITS1-F (Gardes and Bruns 1993) + 5.8S (Vilgalys and Hester 1990) and 5.8S-R (Vilgalys and Hester 1990) + ITS4 (White et al. 1990). For other extractions nuITS+nuLSU was amplified in one or two pieces, using the primer pairs ITS1-F + LR5 (Vilgalys and Hester 1990), ITS1-F + LR3 (Vilgalys and Hester 1990) and LR0R (Rehner and Samuels 1994) + LR5, or ITS1-F + ITS4 and LR0R + LR5. The region mtSSU was amplified using the primer pair mrSSU1 + mrSSU3R (Zoller et al. 1999). For our dating analysis, two additional genes were obtained for a small subset of fresh specimens, the nuclear small subunit ribosomal RNA gene (nuSSU) and the second largest subunit of RNA polymerase II gene (*RPB2*). The nuSSU was obtained employing the primer pair NS1 + NS4 (White et al. 1990). *RPB2* was amplified in two pieces, employing the primer pairs fRPB2-5F + fRPB2-7cR and fRPB2-7cF + fRPB2-11aR (Liu et al. 1999). All primers were purchased from Integrated DNA Technologies (Coralville, IA).

When nuITS+nuLSU was amplified in a single piece, REDExtract-N-Amp PCR ReadyMix (Sigma-Aldrich, St Louis, MO) was used; when amplified in multiple parts or amplifying nuSSU, EconoTaq DNA Polymerase (Lucigen, Middleton, WI) was used. Amplification was performed for mtSSU and *RPB2* using Q5 High-Fidelity DNA Polymerase (New England BioLabs, Ipswich, MA). All PCR reactions were performed using 5 μL of full strength, 1/10 dilution, or 1/100 dilutions of the DNA extracts as templates in a total reaction volume of 25 μL and utilised either a Mastercycler ep Gradient (Eppendorf, Hamburg, Germany) or a C1000 Touch Thermal Cycler (Bio-rad, Hercules, CA). All PCR protocols are included in File S1.

PCR products sometimes contained multiple bands. In these cases, the band of interest was excised from a 2% agarose gel and purified using either a QIAquick Gel Extraction Kit (QIAGEN) or a Monarch DNA Gel Extraction Kit (New England BioLabs). Otherwise, single-band PCR products were purified with a QIAquick PCR Purification Kit (QIAGEN) or a Monarch PCR & DNA Cleanup Kit (New England BioLabs). In the case of faint PCR products, reamplification was performed using 5 μL of a 1/100 dilution of the previous PCR product as template in a total reaction volume of 25 μL using the same polymerase, primers, reaction recipe, and cycling parameters as previously.

In preparation for sequencing, all purified products were run on a 1% agarose gel with 0.0001% GelRed Nucleic Acid Stain, 10,000x in Water (Biotium, Hayward, CA) added for DNA visualisation and using Gel Loading Dye Purple (6x), no SDS (New England BioLabs). UV photographs of gels were taken with an AlphaImager EP (Alpha Innotech, San Leandro, CA), and band fluorescence was estimated using the AlphaView software (Alpha Innotech). Purified PCR product concentration was assessed by comparison with the fluorescence of the bands in Low DNA Mass Ladder (Invitrogen, Carlsbad, CA) run on the same gel. All PCR products of all genes were sent to GeneWiz Inc. sequencing facilities (Cambridge, MA) for Sanger Sequencing. The forward and reverse sequences from each PCR product were edited and a consensus sequence generated using Sequencher v. 5.1 (GeneCodes, AnnArbor, MI). All sequences were submitted to GenBank, with accession numbers listed in Table S1. Our alignments were submitted to TreeBase (S27765).

### Assembling published Sareomycetes sequences

Sequences of species in *Sareomycetes*, either already identified or identified by us through BLAST similarity, are available on public databases such as GenBank, UNITE (Nilsson et al. 2019), and the NARO Genebank Microorganism Search System (Genebank Project 2020). Those nuITS sequences used from these databases were restricted to complete or nearly complete (>450 bp). The identified sequences were obtained by searching GenBank and the NARO Genebank Microorganism Search System for *Sarea*, *Sarea resinae*, *Sarea difformis*, or *Arthrographis lignicola* and downloading those sufficiently complete nuITS and nuLSU sequences (71 and 19 sequences, respectively).

Unidentified and misidentified sequences were found by searching GenBank using the Nucleotide Basic Local Alignment Search Tool (BLAST) (Altschul 1997) with nuITS, nuLSU, and mtSSU sequences derived from morphologically identified specimens. The "distance tree of results" feature was employed, with sequences identified as *Lecanoromycetes* species excluded from consideration. The remaining sequences on branches with or adjacent to identified *Sareomycetes* sequences were downloaded and identified by comparison to further sequences of identified specimens. This yielded an additional 30 sequences. Two of these were discarded because they were identified as chimeric by BLASTing their nuITS1 and nuITS2 portions separately. This method was used to determine that KF274061 consists of a nuITS1 region from *Sarea resinae s. lat.* and a nuITS2 region from an unidentifiable fungus with affinities to *Leotiomycetes*, and KM104053 consists of a nuITS1 region from *Sarea difformis s. lat.* and a nuITS2 region from *Sarea resinae s. lat.* In addition, the UNITE database was searched by examining sequences unique to the UNITE database included in the 8 species hypotheses for the genus *Sarea* and the 11 species hypotheses for the genus *Arthrographis*. These were downloaded and identified by comparison with sequences of identified specimens; low similarity sequences were discarded. In this way, 8 sequences were identified.

Host, locality, and specimen/culture strain data were determined for all published sequences by consulting the information provided in the source database, relevant publications, and relevant culture collection databases (*e.g.*, ATCC 2020; Westerdijk Fungal Biodiversity Institute 2020; University of Toronto 2021). These data as well as accession numbers and updated identifications are included in Table S1.

### Sequence alignments

MAFFT v. 7.308 (Katoh 2002; Katoh and Standley 2013) was used to generate a multiple sequence alignment (MSA) independently for each marker with the following parameters: the FFT-NS-I x1000 algorithm, the 200PAM / k = 2 scoring matrix, a gap open penalty of 1.5 and an offset value of 0.123. The resulting alignments were manually optimised in Geneious v. 9.0.2 (a) to replace gaps at the ends of shorter sequences with an IUPAC base representing any base (“N”), and (b) to trim ends of longer sequences in the nuITS MSA that included part of the 18S–28S ribosomal subunits. The software GBlocks v. 0.91b (Castresana 2000) was used to automatically remove ambiguously aligned regions in the nuITS and mtSSU MSAs using the least stringent parameters but allowing gaps in 50% of the sequences.

### Phylogenetic tree inference

The online version of RAxML-HPC2 hosted at the CIPRES Science Gateway (Stamatakis 2006; Stamatakis et al. 2008; Miller et al. 2010) was used to estimate a three-locus phylogeny under a Maximum Likelihood (ML) framework based on a dataset comprising specimens with at least two available sequenced markers. Several specimens of *Pycnora* were included as outgroup to root phylogenetic trees. Prior to concatenation, and to test for topological incongruence among sequence datasets, we inferred ML trees independently for each locus with RAxML-HPC2, using 1000 bootstrap pseudoreplicates, and assumed bootstrap values ≥70 % as significant for conflicting relationships among the same set of taxa (Mason-Gamer and Kellogg 1996). Because no conflicts were detected, the RAxML analysis was conducted using the GTRGAMMA substitution model for the four delimited partitions (nuITS1+2, 5.8S, nuLSU, mtSSU) and 1000 rapid bootstrap pseudoreplicates were implemented to evaluate nodal support. Evolutionary relationships were additionally inferred in a Bayesian context using MrBayes v. 3.2.6 (Ronquist et al. 2012). Optimal substitution models and partition schemes for these four sequence data partitions were estimated with PartitionFinder v. 1.1.1 (Lanfear et al. 2012) considering a model with linked branch lengths and the Bayesian Information Criterion (BIC). This analysis favoured the SYM+Γ model for the nuITS1+2 partition, the K80+I+Γ for the 5.8S+nuLSU, and the HKY+I+Γ for the mtSSU. The analysis was then conducted with two parallel, simultaneous four-chain runs executed over 5 × 10^7^ generations starting with a random tree, and sampling after every 500^th^ step. The first 25% of data were discarded as burn-in, and the 50% majority-rule consensus tree and corresponding posterior probabilities were calculated from the remaining trees. Average standard deviation of split frequencies (ASDSF) values below 0.01 and potential scale reduction factor (PSRF) values approaching 1.00 were considered as indicators of chain convergence. Tree nodes showing bootstrap support (BP) values equal or higher than 70 % (RAxML analysis) and Bayesian posterior probabilities (PP) equal or higher than 0.95 (MrBayes analysis) were regarded as significantly supported. Phylogenetic trees were visualised in FigTree v. 1.4 (Rambaut 2012) and Adobe Illustrator CS5 was used for artwork.

### Species discovery-validation approach

Based on the existence of well-delimited and highly supported clades in the three-locus phylogenetic tree inferred above, we conducted a preliminary exploration of species boundaries independently for the orange and black *Sarea*. To this end, we used the distance-based Automatic Barcode Gap Discovery method (ABGD) (Puillandre et al. 2012), restricting the analyses to specimens with available data for the fungal barcode nuITS. The analyses used the Kimura two-parameters (K2P) model to estimate genetic distances, a transition/transversion value of 3.95 (orange *Sarea*) and 3.07 (black *Sarea*) calculated with MEGA v.5.2 (Tamura et al. 2011), a *Pmax* of 0.01, and different values for the relative gap width (*X*). Subsequently, the Bayes Factor Delimitation (BFD) method, which allows for topological uncertainty in gene trees and incongruences among gene trees, was chosen to compare two species boundary hypotheses generated for the black *Sarea* on the basis of our morphological study of the specimens, and the ABGD and phylogenetic results (Table 1). *BEAST (Heled and Drummond 2010; Drummond et al. 2012) was used to build the two competing models. These comprised a three-locus dataset in which specimens with identical sequences were removed to avoid sequence redundancies; the number of specimens left was 85, including outgroup specimens. The same optimal substitution models and partition schemes selected in the MrBayes analysis were used for the *BEAST analyses except for the substitution model TrNef+I+Γ, which was preferred for the 5.8S+nuLSU partition. An uncorrelated relaxed lognormal molecular clock was chosen for the three markers based on a preliminary assessment of the adequacy of strict clocks in MEGA 5.0 (Tamura et al. 2011) (see Table S2). The mean clock rate was fixed to 1.0 for nuITS whereas rates were co-estimated for nuLSU and mtSSU under a uniform prior (1 × 10^-5^, 5). A birth-death process tree prior was imposed after conducting preliminary Bayes factors comparisons of Maximum Likelihood Estimates (MLE) calculated with Path Sampling and Stepping-Stone (Lartillot and Philippe 2006; Xie et al. 2011) for models implementing alternative tree priors (see Table S2). By using this tree prior we accommodated incomplete sampling and speciation of nodes in the topology. The *BEAST analyses used a piecewise linear and constant root model for population size (Grummer et al. 2014). Hyperpriors for the birth-death process tree prior and species population mean were given an inverse gamma distribution with an initial value of 1 or 0.1, shape parameter of 1 or 2 and scale of 1 or 2, respectively. Default (but informative) priors were given for the remaining parameters across all analyses. Finally, *BEAST runs of 1.5 × 10^8^ generations, saving every 15000th tree, were performed using the CIPRES Science Gateway (Miller et al. 2010). Tracer v.1.7 (Rambaut et al. 2018) was used to check for convergence, assumed if effective sample sizes (ESS) were > 200. Then, MLE for the two species boundary models were calculated using Path Sampling and Stepping-Stone, with default settings. Bayes Factors were calculated following Hedin et al. (2015). 2lnBF > 10 indicate very strong evidence against a model as compared with the best (Kass and Raftery 1995).

**Table 1:** Species delimitation hypotheses in *Sarea*

	Distinct species	Motivation	Path Sampling		Stepping-Stone	
			Ln (Marginal Likelihood)	2ln (Bayes Factor)	Ln (Marginal Likelihood)	2ln (Bayes Factor)
Model 1 (three *Sarea* spp.)	*Sarea difformis* / *S. coeloplata* 1 / *S. coeloplata* 2	Morphological observations and three-locus phylogenies (RAxML and MrBayes)	**-7867.9101**	**N/A**	**-7868.3128**	**N/A**
Model 2 (two *Sarea* spp.)	*Sarea difformis* + *S. coeloplata* 1 / *S. coeloplata* 2	ABGD nuITS	-7873.5589	11.2976	-7874.1365	11.6474

Marginal likelihood and Bayes factor values for two alternative species delimitation hypotheses in *Sarea* and their motivation. The best model is highlighted in bold

### Polymorphism statistics, haplotype networks, and neutrality tests

DNA polymorphism was assessed for each candidate species delineated by the species discovery-validation approach. The software DnaSP v.5.10 (Librado and Rozas 2009) was used to compute the number of segregating sites (*s*), number of haplotypes (*h*), haplotype diversity (*Hd*) calculated without considering gap positions, and nucleotide diversity (*π*) using the Jukes and Cantor (1969) correction. For these calculations, we used the original nuITS dataset (*i.e.*, not processed with GBlocks), and the GBlocks-trimmed mtSSU alignment because of the high occurrence of large indels. In the nuLSU dataset, three black *Sarea* sequences and 19 out of 51 orange *Sarea* sequences were removed due to the high number of “N” base calls. Next, statistical parsimony using the method TCS (Clement et al. 2002) as implemented in PopART v.1.7 (Leigh and Bryant 2015) was used to infer relationships among haplotypes of the orange and black *Sarea s*.*lat*. These haplotypes were inferred with DnaSP v. 5.10 considering sites with alignment gaps and removing invariable sites and were labelled according to their geographic origin. Finally, deviations from neutrality, which are useful for interpreting past population size changes, were tested with Tajima’s *D* and Fu’s *Fs* statistics in DnaSP v.5.10 using the number of segregating sites. The significance of these tests was assessed based on 10^4^ coalescent simulations.

### Estimating the age of the crown node of *Sareomycetes*

To infer the age of the crown node of class *Sareomycetes*, a six-locus dataset was compiled using sequences from nine *Sarea s*.*lat*. specimens and sequences retrieved from GenBank representing major clades in the *Ascomycota* tree of life. For ascomycete taxa compilation, we followed Pérez-Ortega et al. (2016), Lutzoni et al. (2018) and Voglmayr et al. (2019). Together with the four basidiomycete species included as outgroup, the final dataset consisted of 169 taxa (Table S3).

Alignments of the nuSSU, nuLSU, mtSSU, *RPB1*, *RPB2* and *tef1-α* were carried out in MAFFT v. 7.308 as implemented in Geneious v. 9.0.2 using the same algorithm parameters as above. Manual optimisation of the resulting MSAs consisted in removing clearly ambiguously aligned and intronic regions in rDNA marker datasets (nuSSU, nuLSU, and mtSSU), as well as non-coding regions (introns) in the protein-coding markers (*RPB1*, *RPB2*, and *tef1-α*). Sequences of the latter three datasets were also translated into amino acids to spot misaligned regions generating stop codons. Finally, “N”s were used to fill gaps at the ends of shorter sequences. The resulting alignment lengths were: nuSSU (1629 bp), nuLSU (1305 bp), mtSSU (651 bp), *RPB1* (1100 bp), *RPB2* (2001 bp), *tef1-α* (1209 bp), for a total length of 7895 bp. PartitionFinder v. 1.1.1 was used to estimate the optimal number of partitions of the data along with their corresponding best-fitting nucleotide substitution model using the linked branch lengths option and the Bayesian Information Criterion for model selection. Eight independent data blocks were suggested: (1) nuSSU; (2) nuLSU; (3) *tef1-α* codon1; (4) *tef1-α* codon2, *RPB1*-codon2, *RPB2*-codon2; (5) *tef1-α* codon3; (6) *RPB2*-codon1, *RPB1*-codon1; (7) *RPB2*-codon3, *RPB1*-codon3; and (8) mtSSU. The GTR+I+Γ substitution model was selected for all partitions but 1 (SYM+I+Γ), 2 (TRN+I+Γ), 3 (HKY+I+Γ), and 5 (GTR+Γ). Before assembling the six-locus dataset, potential topological conflicts among markers were visually explored on single-locus ML phylogenetic trees calculated with the online version of RAxML-HPC2 with 1000 bootstrap pseudoreplicates conducted to retrieve nodal support values.

Among all available fossils that may be used to calibrate a class-wide fungal phylogeny (Lücking and Nelsen 2018; Samarakoon et al. 2019), we chose six ascomycete fossils, whose details and associated reference publications are in Table S4. Divergence times and a tree topology were then co-estimated in BEAST v. 1.8.1. XML files were prepared in BEAUti v 1.8.1 (Drummond et al. 2012) using the above-mentioned six-locus dataset with the corresponding partitions and nucleotide substitution models. Additional settings included selection of an uncorrelated lognormal relaxed clock for each marker and a birth-death prior, and the use of a rooted, strictly-bifurcating ML topology obtained in RAxML as a starting tree. This ML tree was previously transformed into ultrametric using the function *chronos* in the R package *ape* (Paradis et al. 2004). In the prior settings step, we forced the co-estimation of the average rate of evolution of each locus by setting the priors for the *ucld.mean* parameter to uniform (10^-5^, 0.01). The taxa and prior distributions used to set the fossil calibrations are detailed in Table S4. Fourteen independent BEAST runs of 200 million generations each were carried out, logging parameters and trees every 2 × 10^4^ generations. Then, Tracer v. 1.7 was used to check for convergence and mixing, making sure that ESS were well above 200. After implementing an adequate burn-in portion to the sampled trees in each run, a total of 8 × 10^4^ remaining trees were combined in a single file using LogCombiner v1.8.1 (Drummond et al. 2012). Because the resulting file exceeded 6 GB and could not be handled by TreeAnnotator v.1.8.1 (Drummond et al. 2012), we implemented a custom script to generate ten files with 4 × 10^4^ randomly drawn trees each. These were then processed with TreeAnnotator v.1.8.1 to generate ten maximum clade credibility trees with annotated median node heights. Age estimates in million years ago (Ma), 95% High Posterior Density (HPD) intervals, and average substitution rates for markers reported in this study are the result of averaging over these ten annotated tree files.

### Inferring a timeframe for the diversification of Sareomycetes

We implemented a secondary calibration approach in BEAST v.1.8.1 on the concatenated three-marker dataset used in the BFD analysis (see section "Species discovery-validation approach") to estimate a temporal context for the diversification of the main lineages of *Sareomycetes*. First, a time estimate of 120.88 Ma (181.35–75.76 Ma, 95 % HPD) was used to calibrate the crown node of *Sareomycetes* based on results of our previous six-locus dating analysis. This calibration was set as a prior using a normal distribution (mean = 120.88, stdev = 35); average substitution rates for the three loci (nuITS, nuLSU and mtSSU) were co-estimated under a uniform prior (10^-5^, 0.01). For comparison, we additionally estimated divergence ages using four different substitution rates: (a) a mtSSU rate of 3.28 × 10^−10^ s/s/y inferred for the *Sareomycetes* clade in the six-locus dating approach, (b) a nuLSU rate of 2.68 × 10^−10^ s/s/y inferred for the *Sareomycetes* clade as well, (c) a nuITS rate of 2.52 × 10^−9^ s/s/y calculated for the fungal order *Erysiphales* by Takamatsu and Matsuda (2004), and (d) a nuITS rate of 3.41 × 10^−9^ s/s/y calculated for the lichenised fungal genus *Melanohalea* by Leavitt et al. (2012).

For all analyses, clock models were set identical to the BFD analyses whereas tree priors were set to “Coalescent: Constant size” to account for the increased amount of intraspecific diversity included in the dataset. The run consisted of 7.5 × 10^7^ generations, saving every 7500th tree. A 25% of burn-in was selected in the TreeAnnotator step and chronograms were drawn with FigTree v. 1.4.

## RESULTS

### Molecular sequence Data

Molecular data were obtained from 70 collections. From these, we produced 212 sequences: 70 nuITS, 63 nuLSU, 61 mtSSU, 9 *RPB2*, and 9 nuSSU (Tables S1 & S3). The nuITS alignment of the 202 sequences produced *de novo* and downloaded from GenBank was 524 bp long; 192 positions were variable and 38 were singleton sites. After processing the alignment with GBlocks, 482 positions (91% of the original alignment) were retained in 24 selected blocks; 172 positions were variable and 33 were singleton sites. The nuLSU alignment comprised 92 sequences and was 914 bp in length; the number of variable and singleton sites were 87 and 21, respectively. The original mtSSU alignment was composed of 75 sequences and 977 positions, of which 253 were variable and 21 were singleton sites. The use of GBlocks trimmed the alignment to 691 bp (70% of the original alignment), displaying 152 variable and ten singleton positions. Last, the concatenated three-locus (nuITS, nuLSU and mtSSU) dataset used for (a) estimating a phylogeny, (b) species validation with the BFD method, and (c) inferring the timing of diversification of *Sareomycetes* was composed of 87 specimens of which 63 had data for the three loci. The total number of bp was 2088, including 398 variable and 75 singleton sites.

### Phylogenetic reconstructions

**Fig. 4:**
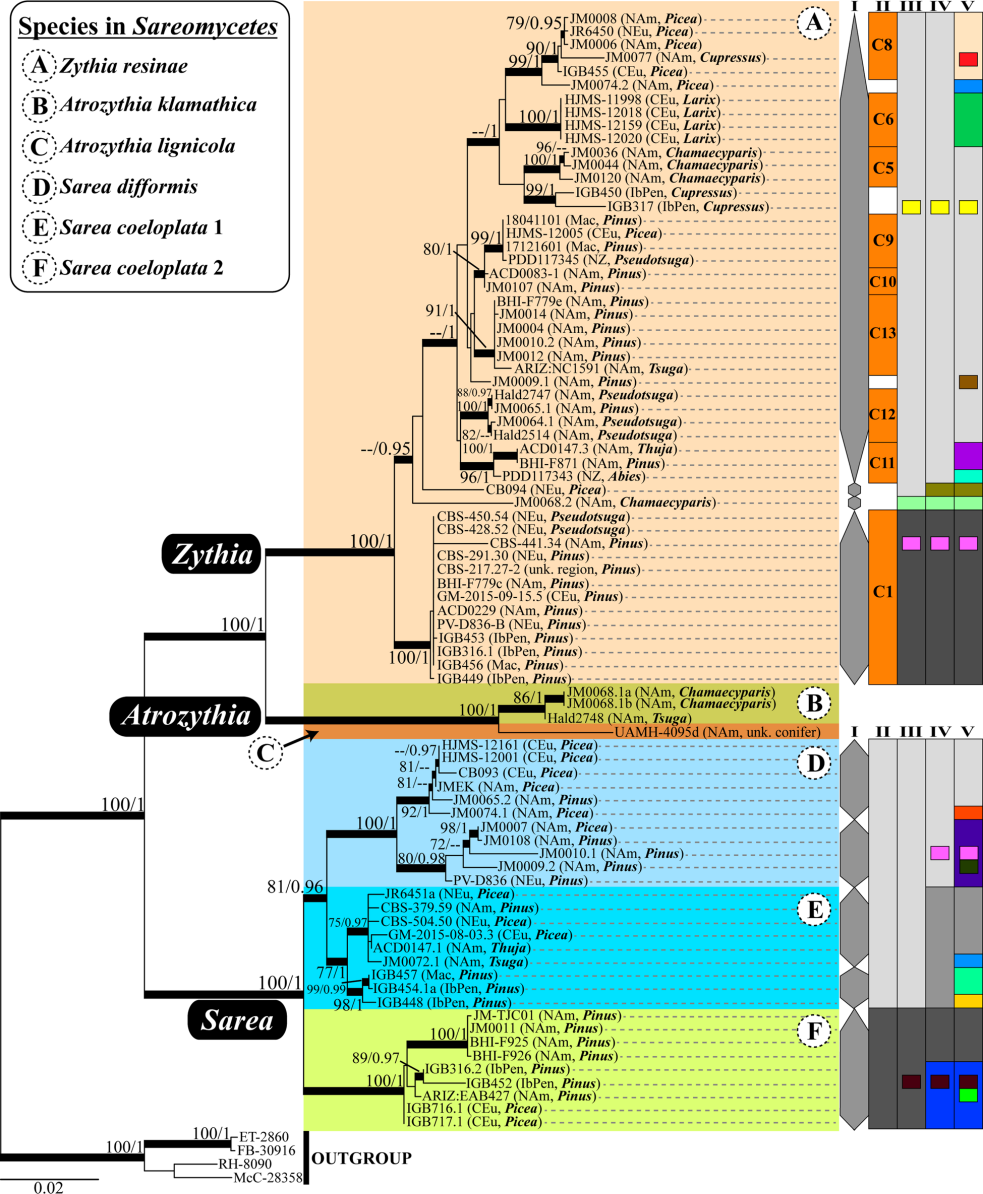
Three-locus RAxML phylogram of *Sareomycetes* with different species delimitation scenarios for *Zythia* and *Sarea*. Phylogram based on a three-locus matrix (nuITS, nuLSU and mtSSU) that depicts relationships among lineages within *Sareomycetes*. The voucher code, the geographic region, and the tree host genus on which each specimen occurred are provided. Coloured boxes delineate the different taxa (genus, species) considered in the present study; full Latin names are available in the legend on the upper-left corner. Bold branches denote high nodal support in the RAxML (bootstrap values ≥ 70%) and/or Bayesian (PP ≥ 0.95) analyses. On the right margin of *Zythia*, species delimitation schemes are based on tree branch lengths and clade support (column I), ecology and distribution (II), and the ABGD 6 (III), 10 (IV) and 24 (V) putative species solutions. On the right margin of *Sarea*, the schemes are based on tree branch lengths and clade support (column I), and the ABGD 2 (II), 3 (III), 7 (IV) and 16 (V) putative species solutions

The single-locus phylogenies produced with RAxML had lnL values of -3158.2564 (nuITS), -2229.9957 (nuLSU) and -2375.8252 (mtSSU). The nuITS and mtSSU phylogenies showed strong nodal support for (a) a clade including all orange *Sarea s*.*lat*. (hereafter referred to as *Zythia resinae*; see section "Taxonomy" below), and (b) a clade assigned to the new genus *Atrozythia* (see section "Taxonomy" below) including two species composed of a few specimens each (Figs S1, S2, S3). The two taxa referenced below as *Sarea coeloplata* 2 and *S. difformis s*.*str*. also formed well delimited and highly supported clades in these two phylogenies; however, *S. coeloplata* 1 was monophyletic with high support only in the mtSSU topology. A supported sister relationship was found for *Zythia* and *Atrozythia*, whereas a clade comprising the three *Sarea* species was only supported in the mtSSU topology, in which *S. coeloplata* 1 and *S. difformis* appeared as sister species. The nuLSU phylogeny only delimited the *S. coeloplata* 2 clade with support, and a specimen assigned to the new species *A. klamathica* was found interspersed in a non-supported clade including *Z. resinae* specimens (Fig. S2). No clear relationships among the main nuLSU lineages were inferred. On the other hand, three-locus phylogenies inferred with RAxML and MrBayes showed high support (100 % BP, PP = 1) for the clades comprising the genera *Zythia*, *Atrozythia* and *Sarea* (Fig. 4). In *Zythia*, these two phylogenetic reconstruction methods were not coherent in delimiting well-supported subclades; only a basal lineage containing samples from Northern and Central Europe, North America, the Iberian Peninsula, and Macaronesia (Cape Verde Is.) showed strong nodal support by both methods, whereas the Bayesian method provided support for at least three inner nodes. The *Atrozythia* clade was split into two well-supported clades, one corresponding to the new species *A. klamathica* (see section "Taxonomy" below), and the other to *A. lignicola*. The *Sarea* clade segregated in three well delimited and supported subclades, each corresponding to a different species: *S. difformis* and *S. coeloplata* 1 and 2. All three lineages are distributed across the Northern Hemisphere (North America and Europe) and occur mainly on *Pinus* and *Picea* resin. Interestingly, in *S. coeloplata* 1, samples from the Iberian Peninsula and Macaronesia (Cape Verde Is.) formed a well-supported subclade sister to the bulk of North American and Northern-Central European specimens. This situation also occurred, although not so markedly, in *S. coeloplata* 2.

### Species delimitation

Based on the topology (*i.e.*, branch lengths) and clade support obtained with the three-locus dataset, at least four lineages in *Zythia* (orange specimens) and five in *Sarea* (epruinose black specimens) might correspond with different species (grey column on the right margin of Fig. 4). The ABGD analyses conducted on nuITS datasets of those genera did not reveal clear barcode gaps. In *Zythia*, ABGD rendered 6, 10, 24 and 52 different partitions (*i.e.*, putative species) when the relative gap width (*X*) was set to 0.5 (Figs. S1, S4), but initial and recursive partitions only converged in the 52-partitions solution. With *X*=1, convergence was found for 1 and 52-partition solutions. In agreement with our morphological data and due to difficulties discussed below in the section "Mixed collections", we hereafter conservatively considered the existence of only one *Zythia* species for assessing genetic polymorphism and phylogeographic structure and calculating neutrality tests. In *Sarea*, although a barcode gap was not strictly found, ABGD analyses using varying levels of *X* (0.5, 1 and 1.5) rendered 2, 3, 6, 7, 16 and 34 different partitions when the relative gap width (*X*) was set to 0.5 and 1 (Figs S1, S5). The two-partition solution suggested the combination of specimens assigned to *S. difformis* and *S. coeloplata* 1 into one single partition (Fig. S5). As this solution contradicted our morphological observations of specimens suggesting the existence of three species in *Sarea*, a hypothesis in agreement with the multi-locus phylogenetic results, we compared the two alternative species delimitation models with the BFD method. Marginal likelihood values for the considered models calculated through Path Sampling and Stepping-Stone are shown in Table 1. Bayes factor comparisons favoured the three species model over the two species model.

### Genetic polymorphism, neutrality tests and phylogeographic structure

Genetic diversity indices, such as the numbers of segregating sites and haplotypes, were greater for *Zythia resinae* than for any *Sarea* species across different markers (Table 2). The nucleotide diversity index behaved in a similar way except for the mtSSU marker: though four times as many specimens of *Z. resinae* as *S. difformis* were included in their respective analyses, *S. difformis* showed slightly higher values than *Z. resinae*. Haplotype diversity values were comparable among species and markers, although *S. coeloplata* 2 consistently showed lower values. However, these results must be interpreted with caution due to the uneven number of studied specimens for each species: for example, *Z. resinae* incorporated three to eight times more individuals in the analyses than the remaining species. Neutrality tests gave significant negative values of Fu’s *Fs* in *S. coeloplata* 1 and *Z. resinae* based on nuITS data (Table 2), indicating a population expansion. Negative values of Tajima’s *D* and Fu’s *Fs* were also obtained for the same species as well as *S. difformis* using the nuLSU dataset; however, these were not statistically significant. Tajima’s *D* tests of mtSSU data generated positive values for all species, but these were not significant as well.

**Table 2:** Polymorphism statistics and neutrality tests for *Sarea* spp. and *Zythia resinae*

Dataset	n	bp	Gaps/missing	***s***	***h***	***Hd***	π (JC)	Tajima’s ***D***	Fu’s ***Fs***
***nuITS***									
*Sarea coeloplata* 1	22	482	48	17	14/20	0.948	0.00662	-1.41635	-7.954(**)
*Sarea coeloplata* 2	15	483	31	17	7/12	0.838	0.01186	0.05176	0.91
*Sarea difformis*	17	482	28	31	13/13	0.956	0.01754	-0.58929	-2.987
*Zythia resinae*	118	511	115	71	48/68	0.96	0.02835	-0.55575	-12.831(*)
***nuLSU***									
*Sarea coeloplata* 1	8	909	382	4	4/7	0.786	0.00251	-0.62573	-0.674
*Sarea coeloplata* 2	8	907	381	13	4/6	0.75	0.00803	-0.84352	1.756
*Sarea difformis*	10	908	415	9	7/7	0.911	0.00548	-0.67784	-2.631
*Zythia resinae*	32	906	226	34	16/17	0.897	0.0114	-0.32928	-1.648
***mtSSU***									
*Sarea coeloplata* 1	5	741	35	12	4/4	0.9	0.00973	1.30583	0.98
*Sarea coeloplata* 2	8	740	17	14	4/4	0.75	0.01013	1.74512	3.209
*Sarea difformis*	11	750	72	33	6/8	0.8	0.01919	0.61079	3.46
*Zythia resinae*	40	691	36	35	14/18	0.931	0.01301	0.08728	-0.796

Polymorphism statistics and neutrality tests results for each marker (nuITS, nuLSU and mtSSU), and *Sarea* spp. and *Zythia resinae*. Columns contain the number of sequences (n), their length (in bp), the number of positions in the alignment with gaps and missing data, the number of segregating sites (*s*), the number of haplotypes (*h*; value after stroke was calculated considering gaps in the alignment), haplotype diversity (*Hd*), nucleotide diversity (*π*) using the Jukes and Cantor (1969) correction, and results of neutrality tests

*: 0.01<*p*-value<0.05; **: *p*-value<0.01

**Fig. 5:**
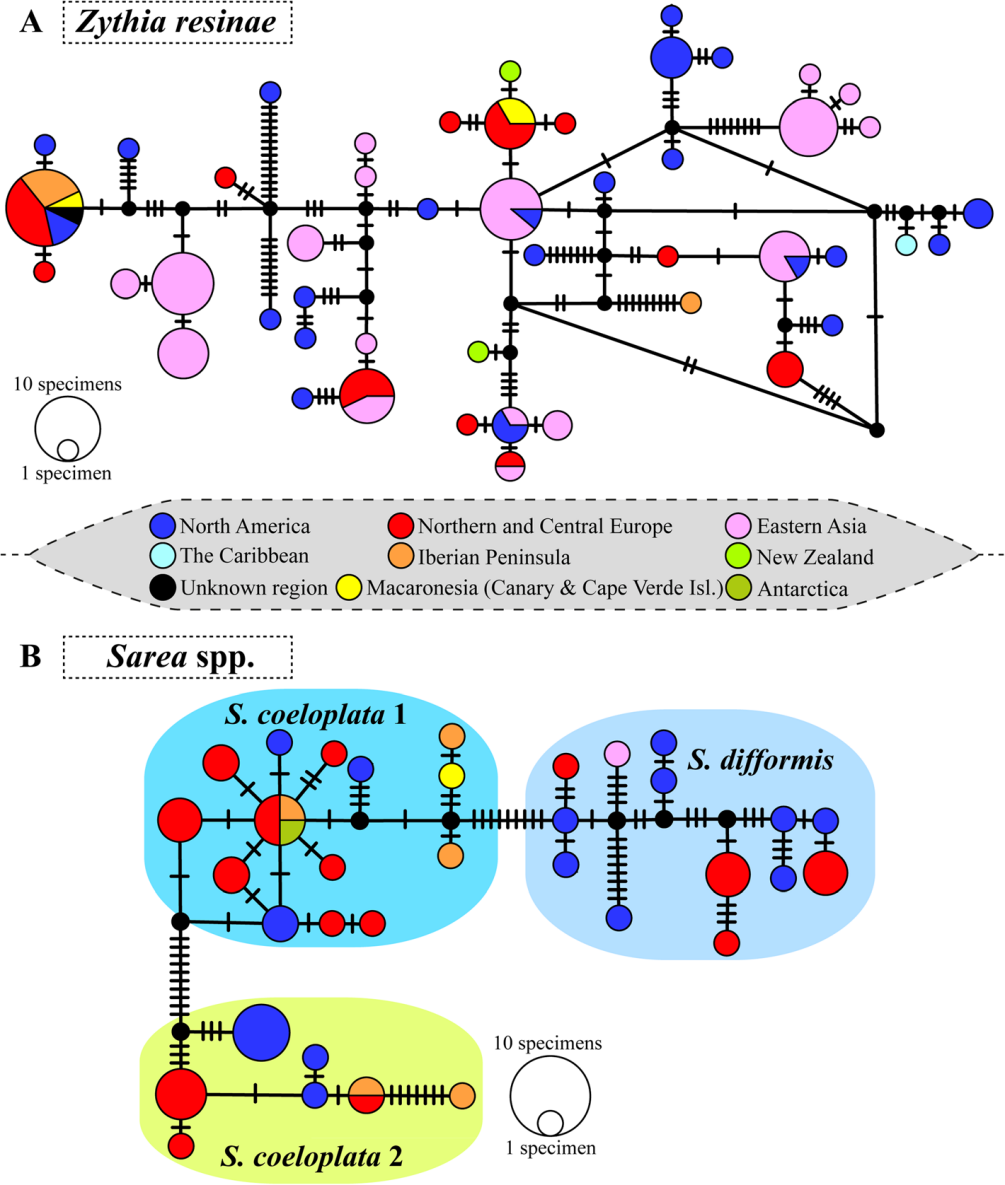
Statistical parsimony networks for *Zythia resinae* and *Sarea* spp. haplotypes. **a** *Zythia resinae*. **b** *Sarea* spp. Haplotypes were coloured according to the geographic origin of specimens (**a** legend is provided for reference). In **b**, a coloured box is used to delimitate each species within *Sarea*. The sizes of the circles in the networks are proportional to the numbers of individuals bearing the haplotype; black-filled smaller circles indicate missing haplotypes. Mutations are shown as hatch marks

Tokogenic relationships among the 48 nuITS haplotypes of *Zythia resinae* revealed no geographic structure as haplotypes from North America, Northern/Central Europe and Eastern Asia were widespread across the network (Fig. 5a). Identical haplotypes were shared among widely distant regions: (a) North America and Eastern Asia; and (b) North America, the whole of Europe, and the Macaronesian islands. The two studied New Zealand haplotypes were not closely related: whereas one was relatively close to a haplotype shared between North America and Eastern Asia, the other was linked to a haplotype shared between Northern/Central Europe and the Macaronesia. The Caribbean haplotype was close to a North American one. As for *Sarea s.lat.*, the network delimited the three considered species well (Fig. 5b). These showed differing levels of intraspecific diversity. For instance, haplotypes of *S. difformis* were separated from each other by a higher number of mutations than haplotypes of *S. coeloplata* 1 and 2. At the geographical scale, whereas haplotypes from many of the considered Northern Hemisphere regions were widespread across the network, we found no haplotypes shared between widely distant localities, except for an Antarctic haplotype shared with Northern/Central Europe and the Iberian Peninsula. These observations may also be due to the limited number of specimens studied compared to the scenario revealed for *Z. resinae*. Finally, in *S. coeloplata* 1 and 2, some Iberian Peninsula and Macaronesian haplotypes showed an increased number of separating mutations; further, *S. coeloplata* 1 haplotypes from these two regions were closely related.

### Age estimates for the crown nodes of Sareomycetes and main lineages within

**Fig. 6:**
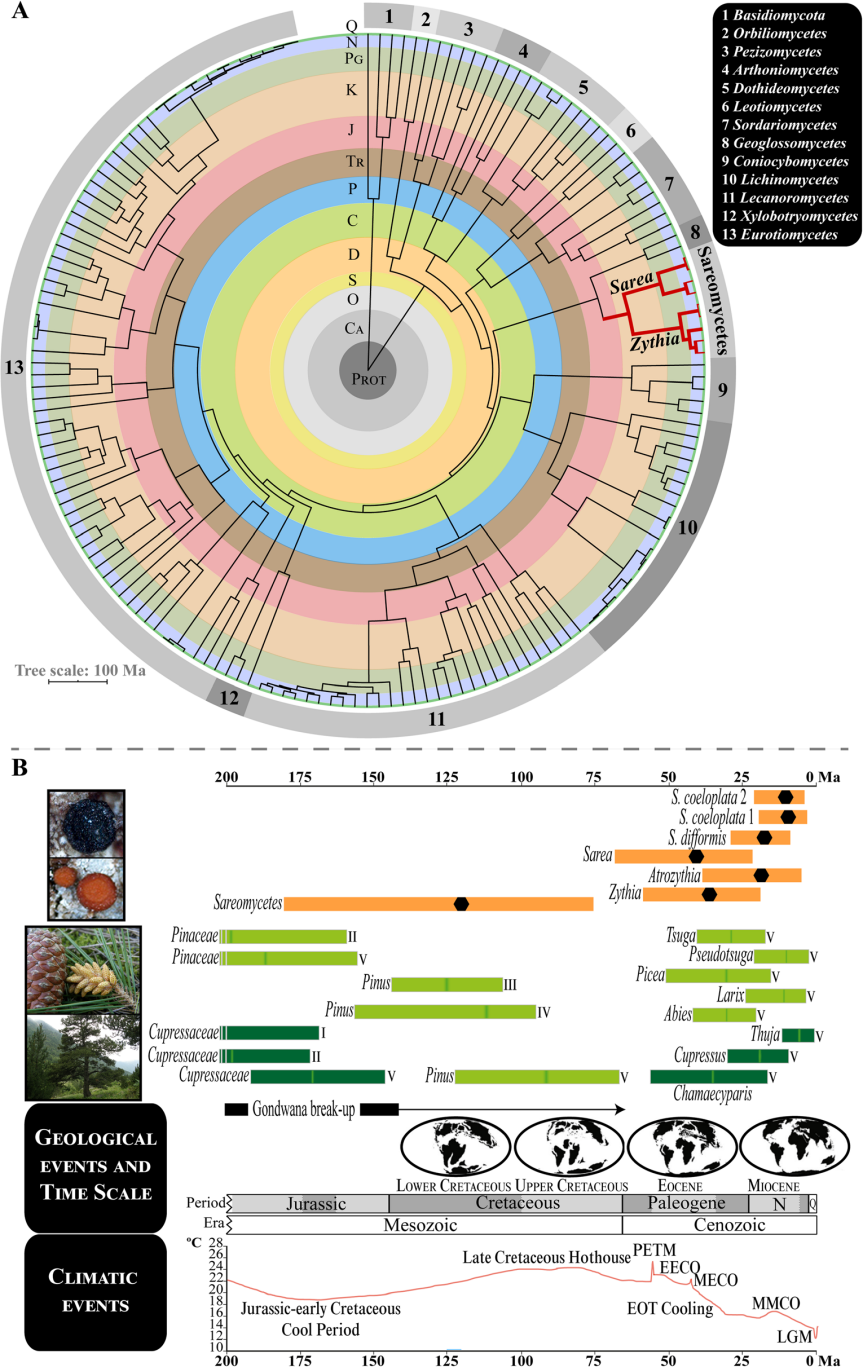
Dating analyses for *Sareomycetes*. **a** Circular time-calibrated MCC tree constructed in BEAST using a six-locus dataset and 169 fungal taxa, including representatives of main *Ascomycota* lineages and *Basidiomycota* (outgroup). The class *Sareomycetes*, comprising *Zythia* and *Sarea* in this analysis, is highlighted in red. Numbers on the chronogram perimeter designate different classes in *Ascomycota* (see legend on the upper-right corner). **b** The 95% HPD age intervals obtained in BEAST to frame in time the crowns of *Sareomycetes* and the three included genera and species; black hexagons represent median ages. Dating results were those obtained using the three-locus dataset and calibrating the crown node of *Sareomycetes* with a time estimate of 120.88 Ma (181.35–75.76 Ma, 95 % HPD) based on results of our six-locus dating analysis. 95% HPD age intervals for the crowns of different gymnosperm plant families and species are represented in boxes coloured with different shades of green (light green, *Pinaceae*; dark green, *Cupressaceae*). These were obtained from different studies: I (Mao et al. 2012), II (Lu et al. 2014), III (Saladin et al. 2017, FBDI approach), IV (Saladin et al. 2017, NDbl approach), and V (Leslie et al. 2018). Paleogeographic maps and climatic graph were drawn after Scotese (2002, 2016). Geological time periods in A and B are shaded and abbreviated as: Quaternary (Q), Neogene (N), Paleogene (Pg), Cretaceous (K), Jurassic (J), Triassic (Tr), Permian (P), Carboniferous (C), Devonian (D), Silurian (S), Ordovician (O), Cambrian (Ca), and Proterozoic (Prot); epochs are abbreviated as Pl (Pliocene), Mi (Miocene), Ol (Oligocene), and Pa (Paleocene). PETM: Paleocene-Eocene Thermal Maximum (55.8 Ma), EECO: Early Eocene Climatic Optimum (54–46 Ma), MECO: Mid-Eocene Climatic Optimum (42 Ma), EOT: Eocene-Oligocene Transition (40–33 Ma), MMCO: Mid-Miocene Climatic Optimum (15–13 Ma), and LGM: Last Glacial Maximum (21000 years ago)

The maximum clade credibility (MCC) tree with 169 fungal taxa and divergence estimates obtained with BEAST showed posterior probabilities (PP) of 1.0 for all inner nodes except for the sister relationship between the clades allocating *Coniocybomycetes*+*Lichinomycetes* and *Lecanoromycetes*+*Xylobotryomycetes*+*Eurotiomycetes* that received a support of PP= 0.96 (Fig. S6). The *Orbiliomycetes* and *Pezizomycetes* formed a clade at the base of *Pezizomycotina* which was dated back to 412.59 Ma (453–400 Ma, 95% HPD). This result is in agreement with the previous dating studies of Beimforde et al. (2014) and Pérez-Ortega et al. (2016). The class *Sareomycetes* was revealed to be sister to *Geoglossomycetes* with high support (PP= 1.0). The split between these two lineages might have occurred during the Middle Jurassic (*ca.* 168.20 Ma; 327.24–109.14 Ma, 95% HPD). The crown node of class *Sareomycetes* was dated to the Lower Cretaceous, *ca.* 120.88 Ma (181.35–75.76 Ma, 95% HPD) according to our six-locus dating using several fossils as calibration points; however, the use of alternative dating methods in our second step (see section “Inferring a Time Frame for The Diversification of *Sareomycetes*” in Materials and Methods above), which was based on a three-locus dataset, provided different time intervals for such an event (Fig. 6; Fig. S7; Table S5). Hence, median age estimates obtained with secondary calibrations drawn from our first, six-locus dating analysis generated similar time intervals as expected (*ca.* 149.37 to 114.81 Ma, Upper Jurassic-Lower Cretaceous), whereas the use of the *Erysiphales* and *Melanohalea* nuITS substitution rates shifted this temporal window towards more recent geological times (Upper Cretaceous-Eocene, *ca*. 72.87–53.1 Ma). We then drew the corresponding rate of evolution of the *Sareomycetes* nuITS from the posterior distribution of our three-locus analysis (first analysis in section “Inferring a Time Frame for The Diversification of *Sareomycetes*” in Material and Methods above) using the parameter *.rate* as reported in FigTree. The value was 1.269 × 10^-3^ s/s/Ma (minimum and maximum 95% HPD values: 8.528 × 10^-5^ and 3.075 × 10^-3^ s/s/Ma) which implies a slower rate of evolution for this region compared to estimates in the *Erysiphales* (2.52 × 10^-3^ s/s/Ma) and *Melanohalea* (3.41 × 10^-3^ s/s/Ma).

The five chronograms inferred for estimating a time frame for the diversification of *Sareomycetes* showed high posterior probabilities supporting relationships among the main lineages except for the sister relationship between *Sarea difformis* and *S. coeloplata* 1 (PP= 0.93–0.94). Similar to previous results, divergence ages obtained with *Erysiphales* and *Melanohalea* nuITS substitution rates generated much more recent time estimates (Table S5). All in all, the origin and diversification of *Zythia*, *Atrozythia* and *Sarea* occurred during the Tertiary (Table S5). Thus, the crown nodes of *Zythia* and *Sarea* were estimated in the Eocene-Miocene, whereas that of *Atrozythia* in the Oligocene-Miocene (Fig. 6). The split between the two *Atrozythia* species (*A. klamathica* and *A. lignicola*) probably occurred during the Miocene. The crown nodes of the three *Sarea* species were placed in the Oligocene-Miocene. Finally, the different dating strategies estimated that intraspecific diversification in the three studied genera occurred < 10 Ma, in the Neogene and Pleistocene (Figs. S8, S9, S10, S11, S12).

## TAXONOMY

Although the terms "holotype" and "lectotype" as defined in Article 9 of the *International Code of Nomenclature for algae, fungi, and plants* (ICN) (Turland et al. 2018) do not apply to names at ranks higher than species, they are used by analogy here to indicate type species of monotypic genera or type species selected by their authors and type species selected by later authors, respectively (Art. 10, Note 1). All specimens have been identified by us unless otherwise indicated. Colour coding refers to Inter-Society Color Council (1976). Full data on additional specimens examined are given in File S2.

***Sareomycetes*** Beimforde *et al.*, *Fungal Syst. Evol.* **6**: 29 (2020).

***Sareales*** Beimforde *et al.*, *Fungal Syst. Evol.* **6**: 29 (2020).

***Zythiaceae*** Clem., *Gen. Fung.*: 128 (1909).

*Synonym*: *Sareaceae* Beimforde *et al.*, *Fungal Syst. Evol.* **6**: 29 (2020).

***Atrozythia*** J.K. Mitch., Quijada, Garrido-Ben. & Pfister, ***gen. nov.***

MycoBank MB838699.

*Etymology*: from the Latin for black (*ater*) and the genus name "*Zythia*," referring to the macroscopic resemblance to *Zythia* species, but with a dark coloration.

*Diagnosis*: Apothecia of *Atrozythia* differ from *Zythia* in their colour (black *vs*. orange) and from *Sarea* because of their white to light blue grey pruina. Paraphyses in *Atrozythia* are unbranched whereas those in *Sarea* are always branched or anastomose, at least in the basal cells. *Zythia* can have unbranched paraphyses but differs from *Atrozythia* in the amount and colour of lipid guttules, orange and abundant *vs*. yellowish and sparse, respectively. *Atrozythia* has a hyaline ectal and medullary excipulum that are sharply delimited by a narrow dark brown pigmented layer; in *Zythia* there is no brown pigmented layer between these layers. In *Sarea* the medullary excipulum is always differentiated by its dark brown colour.

*Holotype species*: *Atrozythia klamathica* J.K. Mitch. & Quijada 2021

*Description*: *Sexual morph*: see description for *Atrozythia klamathica* below. *Asexual morph*: see description of *Arthrographis lignicola* in Sigler & Carmichael, *Mycotaxon* **18**: 502-505 (1983).

*Notes*: This genus currently encompasses two species, both apparently uncommon or under-collected, with one known only in an apothecial morph and the other only in a hyphomycetous asexual morph. Both are found on dead or living conifers; there are some indications of a resinicolous habit in the type species, *A. klamathica*, but additional information is needed to elucidate the ecology of these fungi. In our phylogenetic analyses, the affinities of this group apparently lie closer to *Zythia* than to *Sarea*, but *Atrozythia* species are located on a relatively long branch compared to these two genera. There are apparently no closely matching, unnamed environmental sequences on GenBank assignable to this genus, possibly suggesting rarity rather than merely being overlooked.

***Atrozythia klamathica*** J.K. Mitch. & Quijada ***sp. nov.***

MycoBank MB838700.

*Etymology*: Named after the collection locality of the holotype, Klamath National Forest.

*Diagnosis*: See generic diagnosis above.

*Type*: **USA**: *California*: Siskiyou County, Klamath National Forest, southwest side of Forest Route 17N11, 41°50'03.6" N 123°25'42.1” W, 566 m a.s.l., apothecia on resinous wounds of living young *Chamaecyparis lawsoniana*, 12 Dec. 2017, *J.K. Mitchell JM0068* (FH 00965406 – holotype).

*Description*: *Sexual morph* apothecial. *Apothecia* discoid to cupulate, scattered, erumpent from the resin, consistency coriaceous and ascomata slightly shrunken when dry, but expanding and fleshy when moist, 0.7–1.2 mm diam, to 1 mm high, subsessile to short-stipitate (0.1–0.3 × 0.2–0.3 mm), stipe narrower toward the base. *Disc* concave to plane, round or somewhat irregular by internal growing pressure, smooth or slightly wrinkled, black (267.black) to dark greyish brown (62.d.gy. Br), with or without light white (263.White) to light blue grey (190.l.bGray) coating pruina; margin distinct, raised when immature but not protruding beyond the hymenium when mature, 0.5–1 mm thick, entire and smooth or radially cracked, concolourous with hymenium and usually pruinose. *Receptacle* concolourous with hymenium and margin, strongly roughened, more heavily pruinose, pruina extending downward on the stipe, anchoring hyphae surrounding the receptacle from base of stipe to lower flank and rarely at margin; pruina can be lost during development and is usually more frequent in immature apothecia. *Asci* (103–)131–158(–166) × (27.5–)29.5–36.5(–40.5) μm, cylindric-clavate, multispored, mature asci 35–50 μm below the hymenial surface prior to spore discharge, ascus dehiscence rostrate, inner wall material expanding, protruding *c*. 40–50 μm, reaching the hymenial surface at spore discharge; apex hemispherical, thick-walled, strongly staining in CR, apex with an apical chamber, apical wall 3–5 μm thick, chamber later disappearing and apical tip thickening, becoming 10–15.5 μm thick, projecting into the ascus, becoming dome-like, with intermediate morphologies also observed, inner wall not or faintly amyloid, outer wall intensely amyloid; lateral walls 1–3.5 μm thick, asci covered with an amyloid gel layer; base arising from a perforated crozier. *Ascospores* 1.8–2.3 μm diam, globose to subglobose, hyaline, inamyloid, aseptate, wall slightly thick and with one eccentric medium grey (265.med. Gy) lipid guttule. *Paraphyses* embedded in a thick, hyaline layer of gel, cylindrical, uninflated to medium clavate, straight or slightly wavy, terminal cell (5.5–)6.5–9(–11.5) × 2–3.3(–4.5) μm, covered by a strong yellowish brown (74.syBr) to deep yellowish brown (78.d.yBr) amorphous exudate, lower cells (6.5–)8.5–11 × 2–3 μm, basal cells (12.5–)14.5–18(–20.5) × 1.5–2 μm, simple, unbranched, hyaline, septate, septa strongly staining in CR, basal cells ± equidistantly septate, terminal and lower cells shorter, walls smooth, sparse tiny yellow grey (93.yGray) lipid guttules throughout, from the basal to terminal cells. *Excipulum* composed of two differentiated layers sharply delimited, *ectal excipulum* strongly gelatinised, (111–)127–165(–192) μm thick at lower flank and base, (32–)48–124(–132) μm thick at margin and upper flank, constituted of three layers; *innermost layer* of moderately packed *textura intricata* immersed in a pigmented gel, strong brown (55.s.Br) to dark brown (59.d.Br), with sparse dark greyish yellow (91.d.gy. Y) refractive amorphous lumps; *middle layer* with loosely packed hyaline cells, strongly gelatinised, parallel to each other (sometimes interwoven) and oriented perpendicular to the outer surface, *outermost layer* with shorter, parallel and very tightly packed cells without intercellular spaces, walls pigmented and surrounded by a strong brown (55.s.Br) to dark brown (59.d.Br) amorphous exudate, cortical layer irregular and black (267.Black). *Individual cells* at middle layer of ectal excipulum (5–)6.5–9(–10) × 2–3.5 μm at margin, (6.5–)8.5–12(–15.5) × 2–3 μm at lower flank and base, cell walls 0.5–1.5(–3.5) μm thick. *Medullary excipulum* of slightly gelatinised *textura intricata*, tightly packed, cells neither with intercellular spaces nor particular orientation, (10–)12.5–16.5(–19) × 2–3(–3.5) μm. *Asexual morph* unknown.

*Notes*: This species is known from two specimens (of which the holotype was sequenced twice) and is illustrated in Fig. 3. It was probably also observed once in Alaska (Goff 2020), but no specimen was collected. Little is known about its ecology or possible asexual morphs. Sequence and morphological data are sufficient to separate it from *Sarea* and *Zythia*, and it shows a closer affinity to the latter. Although apparently collected only twice, it is possible (given the rarity with which *Sarea difformis* is found on cupressaceous hosts) that *A. klamathica* is the fungus which was isolated as an endophyte of cupressaceous plants in central Oregon and reported as *S. difformis* (Petrini and Carroll 1981) but due to the lack of detailed data in the report, that supposition can neither be confirmed nor refuted. Culture work with fresh material should be done.

***Atrozythia lignicola*** (Sigler) J.K. Mitch., Garrido-Ben. & Pfister ***comb. nov.***

MycoBank MB838701.

*Basionym*: *Arthrographis lignicola* Sigler, *Mycotaxon* **18**: 502 (1983).

*Type*: **Canada**: *Alberta*: Division No. 13, Westlock, dried culture isolated from conifer wood chips and bark from a logging truck, Feb. 1978, *L. Sigler* [isol. 14 Feb. 1978] (UAMH 4095 – holotype, not seen; UAMH 4095, ATCC 52699, CBS 689.83, IFM 52650, IMI 282334 – ex-type cultures, not seen).

*Description*: *Sexual morph* unknown. *Asexual morph* fully described in the protologue (Sigler and Carmichael 1983).

*Notes*: Although hyphomycetes producing arthroconidia are thus far unknown as asexual morphs among members of the *Sareomycetes*, sequence data generated independently on four separate occasions from ex-type strains place this species as congeneric with *Atrozythia klamathica* (Murata et al. 2005; Kang et al. 2010; Giraldo et al. 2014; Saar 2018). This relationship with *Sareomycetes* has also been suggested in previous phylogenetic analyses (Giraldo et al. 2014). The species has been found both in North America (Sigler and Carmichael 1983; Wang and Zabel 1990; Lumley et al. 2001) and in Europe (Metzler 1997; Arhipova et al. 2011, as ‘*Arthrographis pinicola*’). No sexual morph is known, and as with its congener, *A. lignicola* appears to be rarely found and recognised.

***Sarea*** Fr., *Syst. orb. veg.* **1**: 86 (1825), *nom. sanct.* (Fries, *Elench. fung.* **2**: 14, 1828).

*Lectotype species*: *Peziza difformis* Fr., *nom. sanct.* (designated by Hawksworth and Sherwood 1981: 358).

*Synonyms*: *Coniothyrium* subgen. *Epithyrium* Sacc., *Syll. fung.* **10**: 268 (1892).

*Epithyrium* (Sacc.) Trotter, *Syll. fung.* **25**: 249 (1931).

*Lectotype species*: *Coniothyrium resinae* Sacc. & Berl. (designated by Sutton 1980: 625).

*Biatoridina* Schczedr. *Bot. Zhurn. (Moscow & Leningrad)* **49**: 1315 (1964); *nom. inval.* (Art. 40.1).

*Description*: *Sexual morph* apothecial. *Apothecia* black, erumpent from the resin, discoid, roundish to ellipsoid, coriaceous to fleshy, sessile with broad attachment; hymenium and tissues in section purple or brown, turning blue or without change in KOH. *Asci* clavate, multispored, dehiscence rostrate, apex hemispherical, thick-walled, ascus apex staining strongly in CR, with an apical chamber and thin apical wall, chamber later disappearing and apical tip thickening, projecting into the ascus, becoming dome-like, inner wall not or faintly amyloid, outer wall intensely amyloid and covered with an amyloid gel, base short-stipitate with a crozier. *Ascospores* globose to subglobose, hyaline, inamyloid, aseptate, wall slightly thick and with one lipid guttule. *Paraphyses* embedded in gel, cylindrical, uninflated to slightly clavate, straight or slightly bent at the apex, terminal cell covered by a dark brownish amorphous exudate, lower cells and basal cells hyaline and containing tiny yellowish lipid guttules; branched, usually bifurcate, septa strongly staining in CR, basal cells ± equidistantly septate, but lower and terminal cells shorter, walls smooth. *Excipulum* at margin and upper (-lower) flank composed of two well-delimited layers, ectal and medullary excipulum at lower flank to base not always differentiated, tissues strongly gelatinised. Ectal excipulum with loosely packed cells running parallel to each other and surrounded by hyaline or brownish gel, frequently bifurcated and oriented perpendicular to the outer surface, cortical layer of shorter, parallel, and very tightly packed cells covered by a dark brown to black amorphous exudate. Medullary excipulum of moderately packed *textura intricata*, cells gelatinised, gel dark brown, becoming lighter in the subhymenium. *Asexual morph* pycnidial; see descriptions of *Epithyrium* and *E. resinae* in Sutton (1980: 625-626) and *Sarea difformis* in Hawksworth & Sherwood (1981: 361-362).

*Notes*: The genus *Sarea* here is restricted to the group of species resembling the type, *S. difformis*. The two remaining species detected are morphologically indistinct but see notes under *Sarea coeloplata*.

No obvious morphological differences were detected among the (infrequently encountered) asexual morph of sequenced *Sarea* specimens; as a result, we retain all previously synonymised names with asexual type species as synonyms of *S*. *difformis*.

***Sarea difformis*** (Fr.) Fr., *Elench. fung.* **2**: 14 (1828).

*Synonyms*: *Peziza difformis* Fr., *Syst. Mycol.* **2**(1): 151 (1822), *nom. sanct.* (Fries, *l.c.*).

*Patellaria difformis* (Fr.) Schwein., *Trans. Amer. Philos. Soc.,* n.s. **4**(2): 236 (1832) [1834].

*Lecidea difformis* (Fr.) Nyl., *Observ. Peziz. Fenn.*: 68 (1868); *nom. inval.* (Art. 36.1).

*Tromera difformis* (Fr.) Arnold, *Flora* **57**(6): 85 (1874).

*Lecidea difformis* (Fr.) Nyl. ex Vain., *Meddeland. Soc. Fauna Fl. Fenn.* **2**: 65 (1878); *nom. illegit.* (Art. 53.1).

*Biatorella difformis* (Fr.) Vain., *Meddeland. Soc. Fauna Fl. Fenn.* **10**: 143 (1883).

*Biatora difformis* (Fr.) Willey, *in* Tuckerman, *Syn. N. Amer. Lich.* **2**: 130 (1888).

*Biatorella difformis* (Fr.) H. Olivier, *Mem. Real Acad. Ci. Barcelona,* [n.s.] **11**(15): 264 (1914); *comb. superfl.* (Art. 6.3).

*Biatorina difformis* (Fr.) Kirschst., *Ann. Mycol.* **36**(5/6): 378 (1938).

*Type*: **Germany**: *Bavaria*: im Wald bei Sugenheim, an Fichten [*Picea* sp.] auf ausgeflossenem Harze, 1871, *H. Rehm* [*Ascomyceten* no. 577] (K(M) – neotype, examined and designated by Hawksworth and Sherwood 1981: 366); FH 00995483, FH 01093951 – isoneotypes).

*Tromera sarcogynoides* A. Massal., *Flora* **41**(31): 507 (1858); *nom. inval.* (Art. 35.1).

*Tromera myriospora* var*. sarcogynoides* (A. Massal.) Kremp., *Denkschr. Königl.-Baier. Bot. Ges. Regensburg* **4**(2): 228 (1859); *nom. inval.* (Art. 35.1).

*Tromera myriospora* f. *sarcogynoides* (A. Massal.) Anzi, *Lich. Rar. Langob. Exs.* **7**: 267B (1862); *nom. inval.* (Art. 35.1).

*Lecidea resinae* f*. minor-denigrata* Nyl., *Lich. Lapp. Orient.*: 185 (1866).

*Coniothyrium resinae* Sacc. & Berl., *Atti Reale Ist. Veneto Sci. Lett. Arti,* ser. 6 **3**(4): 739 (1885) [1884-1885].

*Clisosporium resinae* (Sacc. & Berl.) Kuntze, *Revis. gen. pl.* **3**(3): 458 (1898).

*Lichenoconium resinae* (Sacc. & Berl.) Petr. & Syd., *Repert. Spec. Nov. Regni Veg. Beih.* **42**(3): 436 (1927).

*Epithyrium resinae* (Sacc. & Berl.) Trotter, *Syll. fung.* **25**: 250 (1931).

*Type*: **Italy**: *Veneto*: horto Patavino, in resina dejecta uda, *D. Saccardo* (PAD – Hb. Saccardo – holotype, examined by Hawksworth, *Persoonia* **9**(2): 194 (1977).

*Biatoridina pinastri* Schczedr., *Bot. Zhurn. (Moscow & Leningrad)* **49**(9): 1315 (1964); *nom. inval.* (Art. 40.1).

*Description*: *Apothecia* discoid, roundish to ellipsoid, scattered, or gregarious, erumpent from the resin, consistency coriaceous and apothecia slightly to moderately contracted when dry, expanding and fleshy when moist, 0.2–1.3 mm diam, to 0.5 mm high, sessile, entirely black (267.Black). *Disc and receptacle* rough; margin distinct, slightly raised when immature or dry but not protruding from the hymenium after rehydration, 0.5–1 mm thick, rough or radially cracked, concolourous with hymenium and receptacle. *Hymenium* and tissues in section light purple (222.l.P) to deep purple (219.deepP), pigments turning brilliant blue (177.brill. B) to deep blue (179.deepB) in KOH. *Asci* (34–)46.5–53.5(–78) × (9.5–)12.5–14.5(–18.5) μm, clavate, multispored, mature asci 10–30 μm below the hymenial surface prior to spore discharge, ascus dehiscence rostrate, inner wall material expanding, protruding *c*. 9–15 μm, reaching the hymenial surface at spore discharge; apex hemispherical, thick-walled, strongly staining in CR, apex with an apical chamber, apical wall 2-3.5 μm thick, chamber later disappearing and apical tip thickening, becoming 7-11 μm thick, projecting into the ascus, becoming dome-like, inner wall not or faintly amyloid, outer wall intensely amyloid; lateral walls 0.5–1.5 μm thick, asci covered with an amyloid gel layer; base short-stipitate and arising from a crozier. *Ascospores* (1.7–)2.1–2.3(–3) μm diam, globose to subglobose, hyaline, inamyloid, aseptate, wall slightly thick and with one eccentric medium grey (265.med. Gy) lipid guttule. *Paraphyses* embedded in gel, cylindrical, uninflated to slightly clavate, straight or slightly curved at the apex, terminal cell (4–)6–7.5(–11.5) × 1.5–2.5(–3) μm, covered with a deep brown (59.d.Br) to brown black (65.brBlack) amorphous exudate, lower cells (4.5–)7.5–8.5(–11.5) × 1.5–2.5 μm, basal cells (6.5–)9–10(–12) × 1.5–2.5 μm, bifurcate in lower cells, hyaline, septate, septa strongly staining in CR, basal cells ± equidistantly septate, but lower and terminal cells shorter, walls smooth, sparse tiny yellow grey (93.yGray) lipid guttules in all cells. *Excipulum* at margin and upper (-lower) flank composed of two well differentiated layers, lower flank to base not always differentiated into two types of tissues. *Ectal excipulum* strongly gelatinised, (41–)57–67(–92) μm thick at lower flank and base, (28–)49–60(–86) μm thick at margin and upper flank, cells loosely packed and surrounded by a light greyish brown (60.l.gy. Br) to medium brown (58.m.Br) gel, running parallel each other (sometimes interwoven), frequently bifurcated and oriented perpendicular to the outer surface, cortical layer with shorter, parallel and very tightly packed cells without intercellular spaces, walls strongly pigmented and surrounded by a dark brown (59.d.Br) to brown black (65.br. Black) amorphous exudate. *Ectal cells* (6.5–)10–12.5(–18.5) × 1.5–3 μm at upper flank and margin, (7–)11–13.5(–25.5) × 1–2.5 μm at lower flank and base, cell walls 0.5–1.5(–2) thick. *Medullary excipulum* of textura intricata, cells moderately packed and gelatinised, gel dark brown (59.d.Br.) to brown black (65.brBlack), becoming lighter in the subhymenium, cells (6.5–)10–12(–20.5) × (1.5–)2.5–4 μm.

*Notes*: The concept of *Sarea difformis* is here restricted to those specimens presenting a purple pigment in the hymenium which turns blue when a strong base is applied, a character clearly visible in one isoneotype (FH 00995483) and illustrated in Fig. 1. The other isoneotype housed in FH (FH 01093951) is quite poor, with only 2-3 intact apothecia; as a result, only a macromorphological examination was conducted of that specimen.

***Sarea coeloplata*** (Norman) J.K. Mitch., Garrido-Ben. & Quijada, ***comb. nov.***

MycoBank MB838702.

*Basionym*: *Biatorella coeloplata* Norman, *Öfvers. Kongl. Vetensk.-Akad. Förh.* **41**(8): 32 (1884).

*Type*: **Norway**: *Buskerud*: prope Drammen ad Gulskoven [= Gulskogen], *J.M. Norman* (TROM L-565247 – **lectotype designated here**, MBT 395923; MICH 62597 – isolectotype, MBT 395924).

*Synonyms*: ? *Tympanis abietis* P. Crouan & H. Crouan, *Fl. Finistère*: 43 (1867).

*Retinocyclus abietis* (P. Crouan & H. Crouan) J. W. Groves & D. E. Wells, *Mycologia* **48**: 869 (1957) [1956].

*Type*: **France**: *Finistère*, sur la partie rugueuse de l'écorce d'un sapin [*Abies* sp.] abattu, à la base des ergots, *P.M. Crouan & H.M. Crouan* (CO – Hb. Crouan – holotype, examined by Le Gal 1953: 131).

*Biatorella coeloplata* f. *carbonata* Norman, *Öfvers. Kongl. Vetensk.-Akad. Förh.* **41**(8): 32 (1884).

*Type*: **Norway**: *Buskerud*: prope Drammen ad Gulskoven [= Gulskogen], *J.M. Norman* (TROM L-565247 – **lectotype designated here**, MBT 395927).

*Description*: *Apothecia* macroscopically like *Sarea difformis*, sometimes larger, to 1.5 mm diam. Hymenium and excipulum in section light greyish brown (60.l.gy. Br) to dark greyish brown (62.d.gy. Br) and not changing to blue in KOH. *Asci* (30.5–)42.5–45.5(–62.5) × (11.5–)16–17.5(–22.5) μm. *Ascospores* 1.7–2.5 μm diam, morphology indistinguishable from *S. difformis*. *Paraphyses* cylindrical, uninflated to slightly clavate, straight or slightly bent in upper cells, terminal cell (4–)5.5–6(–8.5) × 1–3 μm, covered by a greyish brown (61.gy. Br) to deep brown (59.d.Br) amorphous exudate, terminal cell of lower cells (4–)5.5–6.5(–9.5) × 1–3 μm, terminal cell of basal cells (4.5–)7–8.5(–11.5) × 1–2.5 μm, branched, usually dichotomously and with connections close to terminal cell, but also below, in lower cells and basal cells; all other morphological features like *S. difformis*. *Ectal and medullary excipulum* morphology like *S. difformis*, but differing in colour, light greyish brown (60.l.gy. Br) to dark greyish brown (62.d.gy. Br), ectal excipulum (23.5–)51.5–60(–78) μm thick at lower flank and base, (12.5–)34–44.5(–71) μm thick at margin and upper flank, mostly with strong differentiation in the colour of ectal and medullary cells, being hyaline and surrounding by a colourless gel unlike *S. difformis* which is brownish. *Ectal cells* (5–)7–10.5(–20) × 2–3.5 μm at upper flank and margin, (5–)7–9(–12.5) × 1.5–3.5 μm at lower flank and base, cell walls 0.5–1(1.5) μm thick. *Medullary cells* (3.5–)8–11.5(–19.5) × 1.5–3.5 μm.

*Notes*: A specimen collected by Norman at the type locality and stored under the name *Biatorella coeloplata* in TROM is here designated the lectotype. Norman (1884) described a form, *Biatorella coeloplata* f*. carbonata*, for older apothecia; we use a single specimen to lectotypify this form as well as the species. Since it is clear that even Norman considered the two forms merely different developmental stages of the same fungus, we see no reason to consider this form a separate taxon.

The holotype of *Tympanis abietis* was not available for examination from CO. Its true affinities are unclear, but Le Gal's (1953) statement "L'hyménium est plongé dans une matière brunâtre qui en agglutine les éléments" in her description of the holotype likely place it in one of the two clades we assign to *S. coeloplata s. lat.*; morphological re-examination of the type should be conducted to verify this placement.

The description above applies to both *Sarea coeloplata* 1 and *Sarea coeloplata* 2 as presented in our phylogenetic analyses. We have been unable to separate the two morphologically, and thus we cannot assign the examined type to one clade or the other. We have observed morphological variations among collections (Fig. 1) and are confident that the difficulty of characterizing the members of these two clades may be overcome by careful analyses involving DNA and morphological examination of single apothecia. This will avoid the problem of mixed collections. For more information, see our discussion of mixed collections below.

***Zythia*** Fr., *Syst. orb. veg.* **1**: 118 (1825).

*Lectotype species*: *Sphaeria resinae* Fr. (designated by Clements and Shear 1931: 372).

*Synonyms*: *Tromera* A. Massal., *Flora* **41**(31): 507 (1858); *nom. inval.* (Art. 38.1).

*Tromera* A. Massal. ex Körb., *Parerga lichenol*: 453 (1865).

*Holotype species*: *Lecidea resinae* Fr.

*Retinocyclus* Fuckel, *Jahrb. Nassauischen Vereins Naturk.* **25-26**: 332 (1871) [1871-2].

*Lectotype species*: *Lecidea resinae* Fr. (designated by Hawksworth and Sherwood 1981: 358).

*Pycnidiella* Höhn., *Sitzungber. Kaiserl. Akad. Wiss., Wien. Math.-Naturwiss. Cl., Abt. 1* **124**(1-2): 91 (1915).

*Lectotype species*: *Cytospora resinae* Ehrenb. (designated by Clements and Shear 1931: 372).

*Description*: *Sexual morph* apothecial. *Apothecia* brilliant orange-yellow (67.brill. OY) to deep orange (51.deepO), erumpent from the resin, discoid to cupulate, roundish or slightly ellipsoid, coriaceous and darker when dry, fleshy and lighter after rehydration, hymenium and receptacle concolourous, margin usually differentiated and protruding slightly beyond the hymenium; sessile with broad attachment, sub-stipitate to prominently stipitate. Hymenium and tissue colours not changing in KOH. *Asci* and *ascospores* exhibiting morphology and reactions as in *Sarea*. *Paraphyses* cylindrical, uninflated to slightly or moderately clavate, straight or bent at the apex, completely surrounded by gel that contains hyaline or grey yellow (90.gy. Y) amorphous lumps, all cells with a high amount of brilliant orange-yellow (67.brill. OY) to vivid orange-yellow (66.v.OY) lipid guttules; terminal cell and 1–2 cells below covered by medium yellow (87.m.Y) rough amorphous exudate; usually branched at apical cells or cells below, rarely unbranched, frequently with anastomoses, septa frequently constricted and equidistantly septate with terminal and lower cells shorter (moniliform). *Excipulum and medulla* not well differentiated in section, although two layers can be noted mostly from the margin to the flanks because of the arrangement of cells and amount of pigments. *Ectal excipulum* in lower flank to margin strongly gelatinised, pigmented due to a high amount of brilliant orange-yellow (67.brill. OY) to vivid orange-yellow (66.v.OY) lipid guttules or not pigmented, cells moderately packed and running parallel to each other and surrounded by hyaline gel sometimes including hyaline or grey yellow (90.gy. Y) amorphous lumps, cortical layer with shorter, parallel or unoriented, tightly packed cells without intercellular spaces, amorphous rough exudate covering the cortical cells, hyaline or coloured between deep orange-yellow (72.d.OY) to brown orange (54.brO), usually more abundant at the margin, sometimes even appearing as glassy processes. Amyloid reaction present mostly in the ectal excipulum at the margin and flanks, or absent. *Medullary excipulum* composed of *textura intricata*, cells changing from ectal excipulum to medulla progressively, hyaline, less spaced and gelatinised; subhymenium somewhat similar or differentiated from medulla because of the presence of pigmented lipid guttules, cells without intercellular spaces and without gel. *Asexual morph* pycnidial; see descriptions of *Pycnidiella* and *P. resinae* (Ehrenb.) Höhn. in Sutton (1980: 544) and *Sarea resinae* in Hawksworth & Sherwood (1981: 365).

*Notes*: The history of typification in the genus *Zythia* is somewhat complicated. This is due both to the sparse protologue and apparent confusion among some authors as to whether or not Fries' *Sphaeria resinae* had been a combination of Ehrenberg's *Cytospora resinae*. This has been discussed at length in a recent publication on the matter (Mitchell and Quijada 2020).

***Zythia resinae*** (Ehrenb.) P. Karst., *Meddeland. Soc. Fauna Fl. Fenn.* **14**: 104 (1887) [1888].

*Synonyms*: *Cytospora resinae* Ehrenb., *Sylv. mycol. berol.*: 28 (1818); *nom. cons. prop.*

*Tubercularia resinae* (Ehrenb.) Thüm., *Oesterr. Bot. Z.* **30**(10): 313 (1880).

*Knyaria resinae* (Ehrenb.) Kuntze, *Revis. gen. pl.* **2**: 856 (1891).

*Pycnidiella resinae* (Ehrenb.) Höhn., *Sitzungber. Kaiserl. Akad. Wiss., Wien. Math.-Naturwiss. Cl., Abt. 1* **124**(1-2): 91 (1915).

*Type*: [**Germany**: *Berlin*], Hasenheide & Grunewald, *C.G. Ehrenberg* (B 700016297 & HAL 3029 F – syntypes, seen by Braun, *Schlechtendalia* **30**: 19 (2016), but see Mitchell and Quijada 2020).

*Sphaeria resinae* Fr., *Observ. mycol.* **1**: 180 (1815), *nom. sanct.* (Fries, *Syst. mycol.* **2**(2): 453, 1823).

*Nectria resinae* (Fr.) Fr., *Summa veg. Scand.* **2**: 388 (1849).

*Nectriella resinae* (Fr.) Sacc., *Syll. fung.* **2**: 451 (1883).

*Dialonectria resinae* (Fr.) Cooke, *Grevillea* **12**(64): 109 (1884).

*Type*: **Sweden:** *E.M. Fries*, *Scleromyceti Sueciae* 37 (UPS F-541757 – lectotype, examined and designated by Hawksworth and Sherwood 1981: 366; FH 00964792 – isolectotype); typ. cons. prop. for *Cytospora resinae* (proposed by Mitchell and Quijada 2020).

*Lecidea resinae* Fr., *Observ. mycol.* **1**: 180 (1815).

*Peziza resinae* (Fr.) Fr., *Syst. mycol.* **2**(1): 149 (1822); *nom. sanct.* (Fries, *l.c.*).

*Lecidea resinae* (Fr.) Nyl., *Mém. Soc. Imp. Sci. Nat. Cherbourg* **3**: 183 (1855); *comb. superfl.* (Art. 6.3).

*Biatorella resinae* (Fr.) Th. Fr., *Lich. arct.*: 199 (1860).

*Biatorella resinae* (Fr.) Mudd, *Man. Brit. lich.*: 191 (1861); *comb. superfl.* (Art. 6.3).

*Biatoridium resinae* (Fr.) Uloth, *Ber. Oberhess. Ges. Natur-Heilk.* **11**(4): 95 (1865).

*Tromera resinae* (Fr.) Körb., *Parerga lichenol.*: 453 (1865).

*Pezicula resinae* (Fr.) Fuckel, *Jahrb. Nassauischen Vereins Naturk.* **23-24**: 279 (1870) [1869-70].

*Biatora resinae* (Fr.) Tuck., *Gen. lich.*: 169 (1872).

*Sarea resinae* (Fr.) Kuntze, *Revis. gen. pl.* **3**(3): 515 (1898).

*Peziza myriospora* Hepp, *Die Flechten Europas* **6**: 332 (1857); *nom. illegit.* (Art. 52.1).

*Tromera myriospora* (Hepp) Anzi, *Cat. lich. Sondr.*: 117 (1860); *comb. inval.* (Art. 35.1).

*Peziza myriosperma* Hepp, *Abbild. beschr. spor., Synonymen-Register I-XII*: 13 (1860); *nom. illegit.* (Art. 52.1).

*Retinocyclus flavus* Fuckel, *Jahrb. Nassauischen Vereins Naturk.* **25-26**: 332 (1871) [1871-2]; *nom. illegit.* (Art. 52.1).

*Type*: **Sweden**, *E.M. Fries* (H 951143/H-ACH 431 B – lectotype, examined and designated by Hawksworth and Sherwood 1981: 366).

*Tromera xanthostigma* A. Massal., *Flora* **41**(31): 507 (1858); *nom. inval.* (Art. 35.1).

*Tromera myriospora* var*. xanthostigma* (A. Massal.) Kremp., *Denkschr. Königl.-Baier. Bot. Ges. Regensburg* **4**(2): 228 (1859); *nom. inval.* (Art. 35.1).

*Tromera myriospora* f*. xanthostigma* (A. Massal.) Anzi, *Lich. Rar. Langob. Exs.* **7**: 267A (1862); *nom. inval.* (Art. 35.1).

*Peziza resinae* var. *stipitulata* P. Karst., *Fungi Fenniae Exsiccati* **4**: 324 (1866); *nom. inval.* (Art. 38.1).

*Tromera resinae* var*. stipitulata* P. Karst., *Acta Soc. Fauna Fl. Fenn.* **2**(6): 154 (1885).

*Biatorella resinae* var*. stipitulata* (P. Karst.) Boud., *Hist. classific. discomyc. Europe*: 157 (1907).

*Type*: [**Finland**: *Kanta-Häme*:] Mustiala, Dec., *P.A. Karsten*, *Fungi Fenniae Exsiccati 324* (FH 01093952 – **lectotype designated here**, MBT 395925).

*Description*: See description above for *Zythia* and notes below.

*Notes*: The status of the basionym of *Zythia resinae* is somewhat confused, with authors treating *Cytospora resinae* either as a new name or as a new combination of Fries' *Sphaeria resinae*. Examination of the protologue (Ehrenberg 1818) shows no references, direct or indirect, to Fries' earlier name, and Ehrenberg explicitly includes his species in the index of new species and attributes it to himself ("mihi"); we thus accept this as having been a *species novum*. It is desirable to conserve *Cytospora resinae* with the same type as *Sphaeria resinae* (UPS F-541747) because these names are: (1) almost always treated as synonyms, (2) share the same epithet (and thus will demand a replacement name for one if they are taken out of synonymy and included in the same genus), and (3) are likely indistinguishable based on morphological features. This conservation has been formally proposed by Mitchell and Quijada (2020).

We do not provide an additional description for *Z. resinae* here since at present it is the only accepted species in this genus, and our description of the genus serves as a description of this broadly-defined species. It has been noted, however, that collections in our phylogenetic analyses do exhibit morphological variation, some visible in Fig. 2. Examples of this variation were found in the excipular tissues, *i.e.*, a slightly amyloid reaction in the excipulum of specimens in clade 8 (Fig. 2, j2), specimens with sessile apothecia in clades 3, 6 and 9 (Fig. 2, e1, i1, m1) *vs*. stipitate apothecia in clades 5 and 12 (Fig. 2, h1, k1), specimens with a strongly pigmented cortical layer in clades 2 and 3 (Fig. 2, f2, e2), an almost hyaline ectal excipulum in clades 1, 6 and 12 (Fig. 2, g2, i2, k2), ectal excipulum with high content of pigments in clades 9 and 13 (Fig. 2, m2, l2) and margin with glassy processes in clade 12 (Fig. 2, k2) (clade names are from Fig. S1). We also found examples of variation in the hymenium, *i.e.*, the presence of an additional thick amyloid gel layer in specimens in clade 3 (Fig. 2, e5), and paraphyses simple and not branched in the apical or lower cells in clades 6, 8 and 9 (Fig. 2, i9, j9, m9) *vs*. bifurcate or branched at apical cell in clades 2, 3, 6 and 12 (Fig. 2, f9, e9, l9, k9). We have not separated species within what is almost certainly a complex of many species because of questions of the prevalence of mixed collections and our inability to examine type material of *Lecidea resinae*. For additional information, see our discussion of mixed collections and species diversity below.

### Excluded species

#### Lecidea tantilla *and isonyms*

The invalid (Art. 35.2) names "*Lecidea tantilla* Nyl." and "*Lecidea resinae var. tantilla* Nyl.", which are, paradoxically, cited with the same protologue (Nylander 1857a), have historically been considered synonyms of *Sarea difformis*. Two specimens matching the original description were found in H (H-NYL 19509/H9510278 and H-NYL 21581/H9510242) and examined; both proved to be typical *Strangospora pinicola*. The name was accepted and validly published at species level by Leighton in 1871; four of the nine specimens he cites were found in K (Leighton 1871). Of these, the authors were able to examine three prior to herbarium closures due to the ongoing global pandemic (K(M)263364, K(M)263365, and K(M)263366). Two of these were *S. pinicola*, and one specimen was *Strangospora moriformis*. Based on these studies, we propose the following synonymies:

***Strangospora pinicola*** (A. Massal.) Körb., *Parerga lichenol.*: 173 (1860).

*Synonyms*: *Lecidea tantilla* Nyl., *Actes Soc. Linn. Bordeaux, sér. 3* **21**: 363 (1857) [1856]; *nom. inval.* (Art. 35.2).

*Lecidea resinae* var*. tantilla* Nyl., *Actes Soc. Linn. Bordeaux, sér. 3* **21**: 363 (1857) [1856]; *nom. inval.* (Art. 35.2).

*Lecidea tantilla* Nyl. ex Leight., *Lich. Fl. Gr. Brit.*: 354 (1871).

*Biatorella tantilla* (Nyl. ex Leight.) H. Olivier, *Mem. Real Acad. Ci. Barcelona,* [n.s.] **11**(5): 8 (264) (1914).

*Type*: [**United Kingdom**: *England*: West Midlands,] Shropshire, Wilcot [t], 12 May 1871, *W.A. Leighton* (K(M)263366 – **lectotype designated here**, MBT 395926).

### Misapplied names

The specimen issued as "*Lecidea resinae* Fr." under number 277 of Leighton's *Lichenes Britannici Exsiccati* (FH 00964658) is *Biatoridium monasteriense*, which had not been described at the time of issue (Leighton 1858). Mudd (1861), citing this and other specimens, described *Z. resinae* as having a green thallus, brown apothecia, a thin margin, ellipsoid spores, and having been collected on elms (*Ulmus* sp*.*). None of these traits characterise any species in *Sareomycetes*. That his conception of *Z. resinae* was incorrect and at least partly based on *B. monasteriense* is confirmed by Magnusson's examination and reidentification of one of Mudd's specimens in the Rehm herbarium (Magnusson 1935). Mudd (1861) also described the new variety *Biatorella resinae* var*. rubicundula*, which has been accepted as being an synonym of a *Strangospora* species (Fries 1874; Rehm 1889); unfortunately, type material could not be located at K or BM for examination (Angela Bond & Gothamie Weerakoon, pers. comm.). Many subsequent authors refer to specimens cited or issued by Mudd and Leighton (Crombie 1870; Leighton 1872, 1879; Smith 1926), perpetuating this error.

A similar case to the preceding arose in Southern California around the turn of the twentieth century. Hasse reported *Z. resinae* from the area three times, first in a publication by McClatchie (1897), then in two of his own (Hasse 1898, 1908). He describes the substrate of the specimens as bark, and in the last publication describes the species with black apothecia turning brown when moist, and without margins. These features are all uncharacteristic of species in *Sareomycetes*. Examination of a specimen labelled "*Lecidea (Biatora) resinae* Fr." (*i.e.*, *Zythia resinae*) sent by Hasse to George Knox Merrill (FH 00964657) revealed that it was a specimen of *Strangospora moriformis*. Additionally, the collecting information matches that given in his 1898 publication, suggesting that this is the specimen he based that report on. An additional Farlow Herbarium specimen (FH 00480746) matches the collecting information and description of the 1908 publication and was originally determined by Hasse as "*Biatorella resinae* (Fr.)" (*i.e.*, *Zythia resinae*) but later changed by him to "*Biatorella moriformis* (Ach.) Th. Fr." (*i.e.*, *Strangospora moriformis*) with the later identification confirmed by an annotation by Magnusson. These specimens, along with his description, suggest that his concept of *Z. resinae* was at the time partly or completely based on *S. moriformis*, but that he later realised his error. By 1913, Hasse removed *Zythia resinae* from his list of Southern California lichens entirely (Hasse 1913).

## DISCUSSION

### Species diversity

The number of species in *Sarea s.lat.* (*i.e.*, *Sareomycetes*) has long been a matter of discussion. Hawksworth and Sherwood (1981) traced the idea of there being only a single species for both black and orange fungi to Johann (Hepp 1857b: Tab. 37 Fig. 1) superfluous name *Peziza myriospora*, noting that he designated two forms ("a" being orange and "b" being black); in the printed *Synonymen-Register* (p. 13) to vols. I–XII, however, he used the name “*Peziza myriosperma* Hepp” which may have been a *lapsus*, but similarly must be treated as validly published but superfluous as it refers back to no. 332 (Hepp 1860); this name is missing from *Index Fungorum*. If this was a mistake, the mistake was repeated with the publication of the printed *Synonymen-Register* (p. 16) to vols. I-XVI (Hepp 1867). Hepp's designation of these two forms is presumably in the boxed set of the exsiccata (Sayre 1969) as we could not find them in either an example of the unbound exsiccata (FH 00964656), the specimen from the Patouillard Herbarium (FH 00964655), or those Lee Davies (pers. comm.) examined in K(M); each contains a single specimen, and the labels make no mention of colour or forms. A *Sarea* species dominates in both specimens in FH, and Hepp cited “*Synon. Peziza et Lecid. Resinae Fries*” (*i.e. Zythia resinae*) as a synonym of his proposed new name; it is likely that he considered both orange and black fungi to be a single species (Hepp 1857a, 1857b). Consideration of the orange and black apothecia as representing a single species carried into the 20^th^ century (Nylander 1857b, 1866; Koerber 1865; Leighton 1872; Fink 1935). As stated by Hawksworth and Sherwood (1981), the orange and black fungi, each treated as a single species, rested in separate genera (for *Z. resinae*, *Biatorella*; for *S. difformis*, *Retinocyclus*) for much of the 20th century. Based on morphological similarities, they were then united in a single genus, *Sarea*, where they stood as two separate species, easily differentiated by colour, although they noted that the differences in iodine reactions and the different asexual morphs “might be considered possible grounds for separation at the generic level” (Hawksworth and Sherwood 1981).

The current study employed integrative taxonomy (Goulding and Dayrat 2016; Haelewaters et al. 2018; Lücking et al. 2020) to assess the number of species in *Sareomycetes*. In addition to two species in the new genus *Atrozythia*, one previously undescribed and one not previously recognised as a relative of this group, it was determined that the black and orange fungi deserve to each be treated in separate genera. However, species concepts in *Zythia* and *Sarea* cannot be assessed straightforwardly. The phylogenetic structure combined with the distribution of the internal clades in Fig. 4 strongly suggests that cryptic speciation is occurring in both genera, with at least five and four species in *Sarea* and *Zythia*, respectively. The black fungi are recognised as the core genus *Sarea* and are conservatively interpreted here as three phylospecies and two morphospecies based on tree topology and the combined ABGD-BFD species delimitation approach. *Sarea difformis*, the type species of the genus, is quite distinctive and specimens are easily identifiable based on the purple pigment in the hymenium and (sometimes) stipe that turns blue in application of strong base (*e.g.*, Fig. 1, g1-5). The remaining morphospecies and two phylospecies represent *Biatorella coeloplata*, here combined as *Sarea coeloplata*; the type could not be assigned to a single phylospecies due to issues addressed in our discussion of mixed collections. The existence of cryptic speciation is even more evident in the orange fungi, which are recognised in the genus *Zythia* and are provisionally retained as a single species, *Zythia resinae*. The results of morphological and ABGD analyses together with the phylogenetic structure observed in Fig. 4 indicate, however, that there are likely many species. The least conservative estimates in our ABGD analyses suggested the existence of up to 24 or 52 putative species, which in our opinion represent inflated estimates, as is often the case when non-distance based methods for species delimitation are used, such as those that use tree branch lengths or the coalescent (*e.g.* Bayesian Poisson Tree Processes (bPTP) and Generalized Mixed Yule Coalescent (GMYC) models; Pons et al. 2006; Zhang et al. 2013). In contrast, the ABGD solutions involving 6 or 10 species are more in accordance with the phylogenetic structure represented in Fig. 4. Given the contradictions between the different approaches, and due to inability to examine the type specimen of *Lecidea resinae* and the issues caused by mixed collections, we refrain from formally proposing and naming any new species in *Zythia*. Our adoption of this much more conservative vision of species diversity in *Zythia* aims at avoiding falsely circumscribing entities that do not represent actual species, even if it implies failing to recognise clearly delimited entities (Miralles and Vences 2013; Carstens et al. 2013).

Furthermore, it may happen that the well supported clades observed in Figs 4 and S1 merely represent geographic structure of the *Sarea* and *Zythia* datasets. Both genera are widely distributed in Europe, North America, Asia, and Africa, with *Z. resinae* also present in Australasia (Hawksworth and Sherwood 1981; Gadgil and Dick 1999; Beimforde et al. 2020). Records from the Southern Hemisphere almost certainly represent anthropogenic introductions, but the Northern Hemisphere distribution is still broad. Similar broad distributions are known in other taxa, and although they can suggest cryptic speciation (Zhong and Pfister 2004; Stadler et al. 2014; Lücking et al. 2014, 2017; Skrede et al. 2017; Tanney and Seifert 2019), it is not always the case (Pringle et al. 2005; Quijada et al. 2016; Liu et al. 2017; Baral et al. 2018). In addition to the broad geographic range, *Sareomycetes* species are found on the resin of a wide variety of host species. *Sarea* species are found on the resin of seven genera in *Pinaceae* and *Z. resinae* is found on twelve or thirteen genera in *Cupressaceae* and *Pinaceae* (see Table S6). This broad host range is again not necessarily indicative of cryptic diversity (Johnston and Park 2005; Baral et al. 2018), but is suggestive (Herrera et al. 2015; Martinović et al. 2016; Pärtel et al. 2017). Finally, published nuITS sequences assignable to the *Sareomycetes* are variable at levels greater than the standard 3% threshold for species delimitation in fungi (Izzo et al. 2005; Ciardo et al. 2006; Blaalid et al. 2013; Geml et al. 2014; Gweon et al. 2015), and greater than even the genus threshold (5.7% difference) suggested for filamentous fungi in a recent study (Vu et al. 2019). While such thresholds are known to not be constant across kingdom Fungi and thresholding is not an ideal way to delimit species (Nilsson et al. 2008; Kõljalg et al. 2013; Lücking et al. 2020), this is also suggestive of cryptic diversity.

### Biogeography and Host Specificity

Little to no phylogeographic pattern in the studied *Sareomycetes* species is recovered in our analyses. This may be due to the fact that conifers in *Pinaceae* and *Cupressaceae* have been widely introduced around the world for ornamental and commercial purposes (Farjon 2017). We hypothesise that a number of *Sareomycetes* strains have been distributed worldwide, travelling on the resin of hosts or as endophytes. The most obvious example is the introduction of *S. coeloplata* 1 to Antarctica reported in a study of the wood decay fungi on huts dating from the early 20th century (Held et al. 2003; Arenz et al. 2006). This fungus presumably was inhabiting the pinaceous timber brought to build the Discovery Hut on Ross Island (77° S), during the Discovery Expedition (1901–04). Our haplotype network suggests that the origin of that strain was in Northern or Central Europe, where the countries supplying materials for these expeditions are located. The persistence of this species over the course of a century is perhaps an indication of how easy it would be to accidentally introduce these fungi to a new area. Another clear and relatively recent introduction is that of both *Zythia resinae* and *S. coeloplata* 1 to Cape Verde (reported in this study). Since no conifers are native to Cape Verde, we can again be sure that this is a case of human introduction (Hansen and Sunding 1993; Arechavaleta Hernández et al. 2005; Farjon 2017); *Pinus* spp. and *Cupressus* spp. have been widely introduced to Cape Verde (Frahm et al. 1996). At least two haplotypes of *Zythia resinae* and *Sarea coeloplata* 1 from Macaronesia (Cape Verde and the Canary Islands) are identical, or closely related, to haplotypes from the Iberian Peninsula. This makes sense since these archipelagos have close historical relationships with Spain and Portugal. The reports of *Zythia resinae* from New Zealand almost certainly represent a third instance of anthropogenic introduction. *Pinaceae* and *Cupressaceae* are the only families known to host fungi in *Sareomycetes*; of these families, only two species in *Cupressaceae* are native to New Zealand (De Lange and Rolfe 2010), but all reports of *Zythia* are from *Abies*, *Pinus*, and *Pseudotsuga*, in *Pinaceae* (Gadgil and Dick 1999; Beimforde et al. 2020). A final apparent indicator of ease of transmission through wood projects are a series of seven nuITS sequences uploaded to GenBank and misidentified as '*Hormococcus conorum*' and '*Zythia pinastri*' (NCBI, NLM, Bethesda (MD) 2020a, 2020b, 2020c, 2020d, 2020e, 2020f, 2020g). Since these are part of a project titled "Imported wood products to United States as vectors for potential invasive fungal species," it may be surmised that these were generated from imported wood products. On the other hand, the almost complete lack of genetic structure in the geographic distributions of species and the extensive geographic distribution in the Northern Hemisphere of some genetic lineages may be also due to long-distance dispersal of minute spores by wind, or even migratory birds, which use coniferous trees as perches in their migration routes (Hallenberg and Kúffer 2001; Muñoz et al. 2004; Wilkinson et al. 2012; Viana et al. 2016). Based on age estimates for the divergence among closely related haplotypes in all *Sareomycetes* species, intercontinental dispersal of lineages could have occurred during the Quaternary (< 2.59 Ma), and this could have been concomitant with events of population expansion, as suggested by neutrality test results in the nuITS and nuLSU markers. Larger datasets assembled with a population-genetics scope are needed to evaluate these hypotheses. Nevertheless, there are exceptions to this general pattern, since seven clades in total contain only specimens from relatively restricted, and sometimes sympatric, ranges: one from the eastern USA (*Zythia resinae* clade 13 in Fig. S1), one from New England (*Z. resinae* clade 5 in Fig. S1), one from the Pacific Slope (*Atrozythia klamathica*), and three from Japan (*Z. resinae* clades 2, 4, & 7 in Fig. S1). Without broader sampling, particularly in Asia and Africa, and considering all available environmental sequences, it is difficult to determine if these are truly lineages of limited range, or a sampling artifact.

Likewise, there is little overall pattern of host specificity, except perhaps at the host family level. This might be expected, since resin composition is broadly similar within each conifer family (Langenheim 2003; Lambert et al. 2005) but still varies among species (Lambert et al. 2007) and even varies within a single species (Tappert et al. 2011). If there is a pattern of specificity even at family level, it appears not to hold for all species. For example, *Sarea coeloplata* 1 was found growing on *Thuja occidentalis* (ACD0147) in addition to a number of species in *Pinaceae*. Similarly*, Zythia resinae* clade 8 (in Fig. S1) encompasses primarily specimens on *Pinaceae*, but also a specimen found growing on *Cupressus forbesii* (JM0077), and the two known specimens of *Atrozythia klamathica* are from hosts in different families. Although this could be explained by a complete lack of host specificity, an alternative explanation is that different strains/species in *Sareomycetes* in some way selectively grow on resin containing or lacking certain components. Production of specific resin components need not mirror evolutionary relationships (Tappert et al. 2011), so what currently appears random may still contain a hidden pattern. Nonetheless, there are clades suggestive of host specificity at the host generic or specific level, even if most clades are found on mixed hosts. *Zythia resinae* clade 4 (in Fig. S1) contains only samples found associated with *Chamaecyparis obtusa*. *Zythia resinae* clade 5 (in Fig. S1) and an unnumbered clade appearing only in our three-gene and mtSSU analyses appear to be found only on *Chamaecyparis* spp. and *Cupressus* spp., respectively. Perhaps significantly, each of these clades also shows a fairly restricted geographic pattern, noted above, and each of these clades is among the least well-sampled, supported groups in our phylogeny. Wider, more robust sampling could change the pattern seen. Ultimately, a more detailed understanding of the specific ecology of species in *Sareomycetes* is needed to generate and test hypotheses regarding host specificity.

Our dating analyses provide additional insight into host specificity in *Sareomycetes* at the temporal scale (Fig. 6). The results of our dating analyses match well with estimates of the diversification of the tree host genera of these fungi. Our estimate of 120.88 Ma (181.35–75.76 Ma, 95 % HPD) for the crown node of *Sareomycetes* places the origin of this group concurrent with or after the origins of *Cupressaceae* and *Pinaceae* in the Cretaceous Period (Mao et al. 2012; Lu et al. 2014; Leslie et al. 2018), and roughly concurrent with the origin of the genus *Pinus* in the Jurassic Period (Saladin et al. 2017; Leslie et al. 2018). This suggests that the *Sareomycetes* evolved to exploit the new niche of resin provided by *Pinus* or another, now extinct taxon (Smith et al. 2017; Leslie et al. 2018). The origins of the genera *Atrozythia*, *Sarea*, and *Zythia* and subsequent diversification in *Sarea* (specific estimates given in Table S5) also correspond well with a later period of diversification of host genera in *Cupressaceae* and *Pinaceae* of these fungi in the Cenozoic Era (Leslie et al. 2018). This occurred during and following a period of global cooling (Scotese 2016) together with some of the last important geological events, including Cenozoic orogenies, which influenced the worldwide distribution of conifers. Close evolutionary histories among fungi and their hosts are well known in several parasitic and ectomycorrhizal fungal clades (Takamatsu 2013; Sánchez-Ramírez et al. 2017; Looney et al. 2020)

### Mixed Collections

An unexpected complicating problem was uncovered during these investigations. Prior to this study, authors have noted that both *Sarea* and *Zythia* species can be found growing on the same piece of resin (Hawksworth and Sherwood 1981; Spier and Aptroot 2000; Yatsyna 2017). This was noted in our study of specimens: *Atrozythia klamathica* was found growing alongside *Zythia resinae* (JM0068), and *Z. resinae* was found growing with *Sarea difformis* (*e.g.*, PV-D863), *S. coeloplata* 1 (*e.g.*, ACD0147), and *S. coeloplata* 2 (*e.g.*, IGB316). Less obviously, it was discovered that multiple clades of *Z. resinae* or species of *Sarea* can be found mixed in a single collection. This was first seen when sequencing multiple loci for specimen BHI-F779. An initial DNA extraction, PCR, and sequencing yielded sequences matching *Z. resinae* clade 1; a subsequent round of sequencing from the same collection yielded sequences matching *Z. resinae* clade 13. Later, *S. difformis* was detected living alongside *S. coeloplata* 1 (*e.g.*, JM0072) and *S. coeloplata* 2 (*e.g.*, JM0011). This ability to share substrate with closely related species, while ecologically interesting, poses serious challenges to the identification of morphological synapomorphies and matching them with the corresponding phylogenetic clade. Given the frequency with which we have found mixed collections, it cannot be excluded that some of the specimens we sequenced and examined morphologically contain mixes of *S. coeloplata* 1 and 2 or mixes of multiple *Z. resinae* clades. This could account for the lack of consistent morphology observed during our investigations of these species and informs our decision to not name these clades.

Based on our experience, future investigation of this family should be conducted by extracting DNA, examining micromorphology, and performing culture work from single apothecia. While this can be a challenge, given that apothecia are typically <1 mm in diameter, we feel that this is the only reliable way of accurately characterizing this group of fungi.

### Morphological Observations

#### Colour changes in sections

We observed that microtome cut sections of *Zythia resinae* stored out of light in dried gum arabic solution on glass slides for a period of several months showed a marked degradation of pigment. Only the high concentration of pigments in the ectal excipulum and in the epithecium remained evident. A similar pattern was observed in sections permanently mounted in glycerine. In addition to colour loss, the encrusting layer over the ectal excipulum and the epithecium was found to dissolve, further altering morphological characters of the fungus.

Such changes posed a challenge to morphological examination, since they create artificial morphological patterns that differ from those seen in recent or fresh material, or even in fungarium material. For these reasons, to accurately assess pigment-related and other morphological characters, we recommend that any morphological examination of *Zythia* species be done on newly sectioned material rather than material sectioned by previous investigators and stored on glass slides or mounted.

#### Ascus dehiscence

Previous authors have reported the asci of *Sareomycetes* as “lecanoralean” (*i.e.*, “rostrate") and “not functionally bitunicate" (Hawksworth and Sherwood 1981; Nash et al. 2008) or of the broadly defined "archaeascé" type (Letrouit-Galinou 1973). Our observations indicate that all three genera, and *Atrozythia* in particular, have ascus dehiscence characterised by a rupture of the outer layer at the tip of the ascus and protrusion of an inner wall. The inner wall extends some distance beyond the outer wall, varying among species. This agrees with the electron microscopic examination of a *Sarea* species performed by Bellemère (1994a, 1994b). It is not clear in our observations whether there is any zone of full wall separation between the inner and outer layers; we thus agree with the view that this is the "rostrate" type of ascus dehiscence (Eriksson 1981; Bellemère 1994a).

### Ecology

#### Are fungi in Sareomycetes lichenised?

The controversy regarding the ecology of species in *Sarea* and *Zythia* is long-standing; they have often been thought of as lichens. This is reflected in the taxonomy of the synonymous names. This idea goes back to Fries' original publication, in which he placed *Zythia resinae* in the lichen genus *Lecidea* and included the phrase "*crusta tenuissima membranacea contigua cinerascenti*" apparently describing a lichen thallus (Fries 1815). Hawksworth and Sherwood (1981) provided evidence that he had corresponded regarding its possible lichen nature with his colleague, the eminent lichenologist Erik Acharius whom he much respected as the last student to defend his thesis before Linnaeus. Since Fries' time, various authors have included *Sarea* and *Zythia* species among the lichenised fungi (Arnold 1858; Tulasne and Tulasne 1861; von Krempelhuber 1861; Nylander 1866; Vainio 1883; Fink 1935; Tucker and Jordan 1979; Etayo 1996; Purahong et al. 2017). A number of other authors were vaguer. Hepp (1857a) included an unnumbered, mixed specimen of *Zythia resinae* and a *Sarea* sp. in his exsiccata, *Die Flechten Europas*. His opinion of whether it was a lichen or fungus, however, is obscured by the fact that the specimen was provided as an example of something easily confused with the black-apothecial lichen he included as number 332 ('*Calicium inquinans γ. sessile*'). Other authors referred to species in *Sareomycetes* as intermediate between lichens and fungi, sometimes placing them in named groups (*e.g.,* "Lichenes ambigua," "Lichenes parasitici," "Pseudolichenes," "Hybridolichenes," and "Fungilli lichenoides") (Anzi 1860; Fries 1860; Koerber 1865; Ohlert 1870; Lettau 1912). One of the more unusual cases is that of Cappelletti (1924), who stated that *S. difformis* could be found both lichenised and non-lichenised in different samples. This situation is known in some fungi (Wedin et al. 2004, 2006), but that Cappelletti reported this relationship in several resinicolous fungi casts doubt on his observations. Additionally, the concepts of some authors accepting species in *Sareomycetes* as lichens have been based on incorrectly identified material; (see sections “Excluded Names" and "Misapplied Names" above). Other mycologists and lichenologists, including the majority of modern authors, treat species in *Sareomycetes* as non-lichenised. We accept them as non-lichenised fungi.

#### Are fungi in Sareomycetes parasitic?

The occurrence of these fungi on resinous wounds has inevitably raised the question of whether they are parasitic (Kujala 1950; Groves and Wells 1956; Malençon 1979; Hawksworth 1981; Suto and Kanamori 1990). This question has been investigated by attempting to satisfy Koch's postulates, with varying results. The first of these was conducted by Ayers (1941), who used one of his cultures of *Z. resinae* to attempt to infect *Pinus strobus*; he saw no effect. Researchers in the then-north-western-USSR used inoculation studies to investigate a disease of pines. They called the fungus they identified as the causal agent "*Biatoridina pinastri*," which they proved was the asexual morph of a *Sarea* species (Shchedrova 1964, 1965). In a broad study of conifer associated discomycetes, Smerlis (1973) concluded that *Z. resinae* was mildly pathogenic, producing cankers on every pinaceous host tested. An inoculation study conducted in the 1980s to determine the cause of a disease of *Pinus koraiensis* in north-eastern China also found no evidence of infection by *Z. resinae* and identified the true causal agent, *Tympanis confusa* (Sūn et al. 1983; Cuī et al. 1984; Xiang et al. 1985; Xiang and Song 1988; Kobayashi et al. 1990). A similar study in Japan on a disease of *Pinus thunbergii* gave the same results; inoculations with *Z. resinae* produced no symptoms, but inoculations with a species of *Ascocalyx* did (Kobayashi and Kusunoki 1985; Kobayashi and Zhao 1989). Additional studies to determine the causal agent of the resinous stem canker of *Chamaecyparis obtusa* determined that *Z. resinae* did not cause symptoms on hosts in *Pinaceae* or *Cupressaceae*, and identified the causal agent as *Cistella japonica* (Hayashi and Kobayashi 1985; Yokozawa et al. 1986, 1989; Suto 1987, 1992, 1997, 1998; Kobayashi et al. 1990). The varying results and generality of these tests leave unresolved the question of pathogenicity of species in *Sareomycetes*; some authors assume pathogenicity and others accept a saprobic lifestyle, as summarised by Beimforde et al. (2020).

#### Fungi in Sareomycetes as endosymbionts of photosynthetic organisms

Other aspects of the ecology of species in *Sareomycetes* have been established with more certainty. These fungi have frequently been isolated as endophytes of conifers in *Pinaceae* (Petrini and Fisher 1988; Kowalski and Kehr 1992; Giordano et al. 2009; Koukol et al. 2012; Arhipova et al. 2015; Sanz-Ros et al. 2015; U’Ren and Arnold 2016; Marmolejo Monciváis 2018) and *Cupressaceae* (Petrini and Carroll 1981; Suto and Ougi 1999; Sieber 2007). This pattern is consistent with previous studies that have shown both saprobes and parasites living within their potential hosts (Fisher and Petrini 1992; Kogel et al. 2006; Oses et al. 2008). Somewhat more unusually, species in *Sareomycetes* have also been isolated as endophytes of grasses (Sánchez Márquez et al. 2008), mistletoes (Peršoh 2013), and possibly deciduous woody plants (Novas and Carmarán 2008). Apart from the *Pinus*-dwelling mistletoe, which presumably allows the fungus close access to the resin seeping from any wounds created by the mistletoe, the occurrence of these fungi in these various hosts is difficult to explain. A closer look at the cupressaceous endophytisms reveals a similarly difficult-to-explain pattern: a *Sarea* species was isolated (Petrini and Carroll 1981), but the current work represents the first report of a *Sarea* species sporulating on a cupressaceous host. Several studies have found *Sarea* and *Zythia* species living within thalli of foliose and fruticose lichens in Europe and Asia (Peršoh and Rambold 2012; NCBI, NLM, Bethesda (MD) 2018a, 2018b; Masumoto and Degawa 2019; Yang et al. 2020). One group of researchers has apparently even recovered *Sarea coeloplata* 1 (identified as *Hormococcus conorum*) associated with a marine alga, *Fucus vesiculosus*, in the Venetian Lagoon (NCBI, NLM, Bethesda (MD) 2013). Many questions about the ecology of this family remain.

#### The ecology of Atrozythia species

This uncertainty extends to our new genus, *Atrozythia.* Some cellulolytic capacity has been reported for *A. lignicola* (Sigler and Carmichael 1983), and the fungus has been recovered from both diseased (Sigler and Carmichael 1983) and dead/rotting wood (Sigler and Carmichael 1983; Wang and Zabel 1990; Metzler 1997; Lumley et al. 2001; Arhipova et al. 2011) of *Pinus* and *Picea* (although the possibility of isolation from *Populus tremuloides* by Lumley et al. (2001) cannot be excluded entirely). Additional study is needed to determine if *A. lignicola* is resinicolous, since all other members of *Sareomycetes* seem to be, or if it has some other lifestyle. *Atrozythia klamathica*, known thus far from only two specimens, was found fruiting directly on the resin of *Chamaecyparis lawsoniana* and *Tsuga heterophylla*; it presumably shares a similar ecology with other members of *Sareomycetes*.

### Taxonomic Placement

The placement of species in *Sareomycetes* in the fungal tree of life has had a long and confused history, which we attempt to elucidate here with more details than Beimforde et al. (2020). In the late nineteenth century authors grouped species generally among the fleshy discomycetes (Crouan and Crouan 1867; Cooke 1871; Saccardo 1889) and more specifically with *Dermateaceae* (Karsten 1885; Saccardo 1889) or *Patellariaceae* (Fuckel 1871), or among the lichenised fungi in *Lecideaceae*, allied with *Biatora* (Tuckerman 1872; Stein 1879). A number of mycologists between 1889 and 1934 (starting with Rehm) placed species among the *Patellariaceae* (see Table S7). Researchers later placed species variously in *Lecanorales* and *Helotiales*, or declined to place them; for instance, the first (and several subsequent) edition(s) of the *Dictionary of the Fungi, Retinocyclus* is listed as belonging with the lichen fungi or in *Helotiales*, and *Sarea* as being of uncertain placement (Ainsworth and Bisby 1943, 1950). Placement was stabilised in 1981, when Hawksworth and Sherwood, based on morphological similarities with *Agyrium rufum*, placed *Sarea* and *Zythia* in *Agyriaceae* (*Lecanorales*) (Hawksworth and Sherwood 1981). Subsequent molecular evidence indicated that *A. rufum* was unrelated to the remainder of *Agyriaceae* (Lumbsch et al. 2007; Lumbsch and Huhndorf 2010) and resulted in the move of *Sarea* and *Zythia* to *Trapeliaceae* (Hodkinson and Lendemer 2011). Not all authors followed these placements. In the course of an electron microscopical study of asci, Bellemère stated that the placement of both *S. difformis* and *Z. resinae* based on ascus ultrastructure was uncertain, and noted that the two species differed in their method of ascus dehiscence (Bellemère 1994b). This study must be considered with some caution, since the substrate of the *Z. resinae* specimen used was said to be stone, indicating that the specimen was likely misidentified. Schultheis et al. (2001) placed *Sarea difformis* under the heading "*Ascomycetes Incertae Sedis*". The application of molecular techniques was needed to properly place these taxa.

The history of the multiple publications attempting to elucidate the taxonomic position of these fungi using molecular data is outlined by Beimforde et al. (2020). Reliance on these publications is likely the reason for uncertain placements or placements in *Leotiomycetes* by several subsequent authors (Lumbsch et al. 2007; Kirk et al. 2008; Eriksson 2014; Hüseyin and Selçuk 2014; Miadlikowska et al. 2014; Garrido-Benavent 2015). Recently, use of information from six genes and sampling taxa throughout *Pezizomycotina* resulted in the erection of the new class *Sareomycetes* to accomodate *Sarea* and *Zythia* (Beimforde et al. 2020). This placement explains over two centuries of confusion and uncertainty.

## CONCLUSION

Our studies of species in *Sareomycetes* have revealed the existence of three genera, one described as new. *Sarea* is restricted to the group of species traditionally identified as *Sarea difformis*, but shown to be at least three phylospecies, *Sarea difformis s. str.*, with a purple hymenial pigment, and two cryptic species lacking such a pigment and identifiable morphologically with the type of *Biatorella coeloplata*, combined here as *Sarea coeloplata*. *Zythia* is resurrected for *Z. resinae* (syn. *Sarea resinae*), which is retained provisionally as a single, highly diverse species. *Atrozythia* and the new species *A. klamathica* are described, and a combination is made for *Arthrographis lignicola*. The family name *Zythiaceae* is resurrected as an earlier name for *Sareaceae*. This family displays few biogeographic patterns and little evidence of host specificity. It is shown to have arisen in the late Jurassic or Cretaceous; subsequent diversification occurred roughly concurrently with the diversification of *Cupressaceae* and *Pinaceae*. Further work on this family is recommended, including type studies on *Lecidea resinae* and *Tympanis abietis,* use of precise methodologies to study the two phylospecies assignable to *S. coeloplata* and to split the *Zythia resinae* complex, and collection of the data required to do population genetic analyses at least for *Zythia*.

## Supplementary Information

**Additional file 1: File S1.** PCR Protocols Used. PCR recipes (including specific components) and cycling parameters used for amplification of sequences used in this study. Protocols are listed under the primer pair they apply to.

**Additional file 2: File S2**. Full Specimen Citations. Full information for specimens examined, including fine locality data, host, collection date, and collector number.

**Additional file 3: Table S1.** Sequences used in three-locus analyses. Specimens and sequences used in phylogenetic analyses for Figs. 4, 5 and 6b, S1, S2, S3, S4, S5, and S8, S9, S10, S11, S12, with updated identifications, identifiers, holding institutions, collecting localities, host data, and associated references. Unmarked sequences were downloaded from GenBank (https://www.ncbi.nlm.nih.gov/genbank/), * indicates a sequence from the UNITE database (https://unite.ut.ee/), and † indicates a sequence from the NARO Genebank (https://www.gene.affrc.go.jp/databases-micro_search_en.php).

**Additional file 4: Table S2.** Tests for strict molecular clock. Test for strict molecular clock for each locus conducted in MEGA 5 prior to performing the three-locus dating analyses (see section “Inferring a Time Frame for The Diversification of *Sareomycetes*” in Materials and Methods). Tested under two different topologies (ML and Bayesian). *denotes rejection of the null hypothesis (*i.e.*, equal rates).

**Additional file 5: Table S3.** Sequences used in six-locus analyses. GenBank sequences used for dating analyses in Figs. 6a, S6, and S7 arranged alphabetically by class.

**Additional file 6: Table S4.** Calibration fossils. List of the six fossil calibrations used to estimate the age of the crown node of *Sareomycetes* with BEAST based on a six-locus dataset with 169 different fungal taxa. (Ma: million years ago).

**Additional file 7: Table S5.** Divergence time estimates of lineages in *Sareomycetes*. Divergence time estimates (Ma) of *Sareomycetes* and the main lineages within obtained using five different secondary calibration approaches with BEAST. The median (in millions of years, Ma) and 95% HPD intervals (in brackets) are given for each divergence time estimate. For simplicity, the “Epochs” interval for each row is based on the five median estimates and it does not consider the corresponding 95% HPD intervals.

**Additional file 8: Table S6.** Prior host records for *Sarea* spp. and *Zythia resinae*. Some representative literature and specimen database host reports of species in *Zythia* and *Sarea* prior to this study. Host genera are arranged alphabetically by family, and *Cupressus* is given in both its broad sense (encompassing *Callitropsis*, *Cupressus s.str.*, *Hesperocyparis*, and *Xanthocyparis*) and its strict sense, differentiated from *Hesperocyparis*. * indicates a report which is ambiguous.

**Additional file 9: Table S7.** Taxonomic history of *Sareomycetes*. Historical taxonomic placements of genera accepted here in *Sareomycetes*, arranged chronologically with references.

**Additional file 10: Figure S1.** *Sareomycetes* nuITS phylogram and species delimitation scenarios based on ABGD. Maximum likelihood tree reconstruction obtained with RAxML based on nuITS data that depicts phylogenetic relationships among the studied *Sareomycetes* specimens. The voucher code of each sample is provided. Coloured boxes delineate the different taxa (genus, species) considered in the present study; full Latin names are available in the legend on the upper-left corner. Bootstrap support values are shown for each node. On the right margin of *Zythia*, species delimitation schemes are based on ABGD 6 (column I), 10 (II), 15 (III), and 24 (IV) putative species solutions. On the right margin of *Sarea*, the schemes are based on ABGD 2 (column I), 3 (II), 7 (III), and 16 (IV) putative species solutions.

**Additional file 11: Figure S2.** *Sareomycetes* nuLSU phylogram. Maximum likelihood tree reconstruction obtained with RAxML based on nuLSU data that depicts phylogenetic relationships among the studied *Sareomycetes* specimens. The voucher code of each sample is provided. Coloured boxes delineate the different taxa (genus, species) considered in the present study; full Latin names are available in the legend on the upper-left corner. Bootstrap support values are shown for each node.

**Additional file 12: Figure S3.** *Sareomycetes* mtSSU phylogram. Maximum likelihood tree reconstruction obtained with RAxML based on mtSSU data that depicts phylogenetic relationships among the studied *Sareomycetes* specimens. The voucher code of each sample is provided. Coloured boxes delineate the different taxa (genus, species) considered in the present study; full Latin names are available in the legend on the upper-left corner. Bootstrap support values are shown for each node.

**Additional file 13: Figure S4.** ABGD results for species delimitation in *Zythia*. A Histogram showing the distribution of pairwise genetic distances (K2P) among sequences (specimens). B–C Graphs showing the inferred number of clusters (*i.e.*, ABGD partitions or putative species) with different Prior intraspecific divergence (P) values. Analyses in B and C used a value for the relative gap width (*X*) of 0.5 and 1.0, respectively.

**Additional file 14: Figure S5.** ABGD results for species delimitation in *Sarea*. A Histogram showing the distribution of pairwise genetic distances (K2P) among sequences (specimens). B–D Graphs showing the inferred number of clusters (*i.e.*, ABGD partitions or putative species) with different Prior intraspecific divergence (P) values. Different values for the relative gap width (*X*) were used: 0.5 (B), 1.0, and 1.5 (C).

**Additional file 15: Figure S6.** Six-locus phylogram for *Ascomycota* with nodal support. Nodal support calculated for the time-calibrated MCC tree constructed in BEAST using a six-locus dataset and 169 fungal taxa, including representatives of the main *Ascomycota* lineages and *Basidiomycota* (outgroup). The colour of circles indicates the strength of nodal support (see legend on the upper-left corner); the size of each circle was deliberately chosen to fit the size of the node, and therefore has no associated information. The class *Sareomycetes*, which represents the focal group of the present study, is highlighted in red. Accession numbers for each marker and considered species are available in Table S3. Ma: million years ago.

**Additional file 16: Figure S7.** Six-locus phylogram for *Ascomycota* with 95% HPD intervals. Nodal 95% Highest Posterior Density (HPD) intervals estimated for divergence ages in the time-calibrated MCC tree constructed in BEAST using a six-locus dataset and 169 fungal taxa, including representatives of the main *Ascomycota* lineages and *Basidiomycota* (outgroup). The class *Sareomycetes*, which represents the focal group of the present study, is highlighted in red. Accession numbers for each marker and considered species are available in Table S3. Ma: million years ago.

**Additional file 17: Figure S8.** Three-locus MCC tree calibrated using a date inferred from the six-locus analysis. Time-calibrated MCC tree estimated from a concatenated dataset of ribosomal (nuITS and nuLSU) and mitochondrial (mtSSU) markers from specimens belonging into class *Sareomycetes* using BEAST. The tree was calibrated imposing a time estimate of 120.88 Ma (181.35–75.76 Ma, 95 % HPD) on the crown node of *Sareomycetes* based on results of our six-locus dating analysis. Nodal blue bars show the 95% HPD intervals for the estimated divergence ages. The voucher code of each sample is provided. Ma: million years ago.

**Additional file 18: Figure S9.** Three-locus MCC tree calibrated using a mtSSU rate inferred from the six-locus analysis. Time-calibrated MCC tree estimated from a concatenated dataset of ribosomal (nuITS and nuLSU) and mitochondrial (mtSSU) markers from specimens belonging into class *Sareomycetes* using BEAST. The tree was calibrated imposing a mtSSU rate of 3.28 × 10^−10^ s/s/y inferred for the *Sareomycetes* clade in the six-locus dating approach. Nodal blue bars show the 95% HPD intervals for the estimated divergence ages. The voucher code of each sample is provided. Ma: million years ago.

**Additional file 19: Figure S10.** Three-locus MCC tree calibrated using a nuLSU rate inferred from the six-locus analysis. Time-calibrated MCC tree estimated from a concatenated dataset of ribosomal (nuITS and nuLSU) and mitochondrial (mtSSU) markers from specimens belonging into class *Sareomycetes* using BEAST. The tree was calibrated imposing a nuLSU rate of 2.68 × 10^−10^ s/s/y inferred for the *Sareomycetes* clade in the six-locus dating approach. Nodal blue bars show the 95% HPD intervals for the estimated divergence ages. The voucher code of each sample is provided. Ma: million years ago.

**Additional file 20: Figure S11.** Three-locus MCC tree calibrated using a nuITS rate estimated for *Erysiphales*. Time-calibrated MCC tree estimated from a concatenated dataset of ribosomal (nuITS and nuLSU) and mitochondrial (mtSSU) markers from specimens belonging into class *Sareomycetes* using BEAST. The tree was calibrated imposing a nuITS rate of 2.52 × 10^−9^ s/s/y calculated for the fungal order *Erysiphales* by Takamatsu and Matsuda (2004). Nodal blue bars show the 95% HPD intervals for the estimated divergence ages. The voucher code of each sample is provided. Ma: million years ago.

**Additional file 21: Figure S12.** Three-locus MCC tree calibrated using a nuITS rate estimated for *Melanohalea*. Time-calibrated MCC tree estimated from a concatenated dataset of ribosomal (nuITS and nuLSU) and mitochondrial (mtSSU) markers from specimens belonging into class *Sareomycetes* using BEAST. The tree was calibrated imposing a nuITS rate of 3.41 × 10^−9^ s/s/y calculated for the lichenised fungal genus *Melanohalea* by Leavitt et al. (2012). Nodal blue bars show the 95% HPD intervals for the estimated divergence ages. The voucher code of each sample is provided. Ma: million years ago.

## Data Availability

The datasets supporting the conclusions of this article are available in the TreeBase repository, project S27765.
