# Supplementary material for: Sareomycetes: more diverse than meets the eye
Source: IMA Fungus. 2021 Mar 16;12:6. doi: 10.1186/s43008-021-00056-0 (PMC7961326; doi:10.1186/s43008-021-00056-0)
Supplement: Supplementary file 1 — Additional file 1: File S1. PCR Protocols Used. PCR recipes (including specific components) and cycling parameters used for amplification of sequences used in this study. Protocols are listed under the primer pair they apply to. [file 43008_2021_56_MOESM1_ESM.docx]

*NS1-NS4:*

PCR Solution

| Component | Concentration | Volume (µL) |
| --- | --- | --- |
| EconoTaq | 5 U/µL | 0.125 |
| dNTPs | 10 mM each | 0.5 |
| Forward Primer | 10 µM | 1.25 |
| Reverse Primer | 10 µM | 1.25 |
| PCR Buffer | 10x | 2.5 |
| DNA Template | 1x | 5 |
| ddH_2_O | — | 14.375 |
| Total | — | 25 |

PCR Program:

1. 95°C, 5 minutes
2. 95°C, 1 minute
3. 53°C, 30 seconds
4. 72°C, 2 minutes
5. Go to 2, repeat 2-4, 35 times
6. 72°C, 10 minutes
7. 4°C, ∞

*ITS1F-5.8S:*

PCR Solution

| Component | Concentration | Volume (µL) |
| --- | --- | --- |
| EconoTaq | 5 U/µL | 0.125 |
| dNTPs | 10 mM each | 0.5 |
| BSA | 1% in H_2_O | 1 |
| Forward Primer | 10 µM | 1.25 |
| Reverse Primer | 10 µM | 1.25 |
| PCR Buffer | 10x | 2.5 |
| DNA Template | .01-1x | 5 |
| ddH_2_O | — | 13.375 |
| Total | — | 25 |

PCR Program:

1. 95°C, 4 minutes
2. 95°C, 1 minute
3. 53°C, 1 minute
4. 72°C, 45 seconds
5. Go to 2, repeat 2-4, 40 times
6. 72°C, 7 minutes
7. 4°C, ∞

*5.8SR-ITS4:*

PCR Solution

| Component | Concentration | Volume (µL) |
| --- | --- | --- |
| EconoTaq | 5 U/µL | 0.125 |
| dNTPs | 10 mM each | 0.5 |
| BSA | 1% in H_2_O | 1 |
| Forward Primer | 10 µM | 1.25 |
| Reverse Primer | 10 µM | 1.25 |
| PCR Buffer | 10x | 2.5 |
| DNA Template | .01-1x | 5 |
| ddH_2_O | — | 13.375 |
| Total | — | 25 |

PCR Program:

1. 95°C, 4 minutes
2. 95°C, 1 minute
3. 55°C, 1 minute
4. 72°C, 45 seconds
5. Go to 2, repeat 2-4, 40 times
6. 72°C, 7 minutes
7. 4°C, ∞

*ITS1F-ITS4:*

PCR Solution

| Component | Concentration | Volume (µL) |
| --- | --- | --- |
| EconoTaq | 5 U/µL | 0.125 |
| dNTPs | 10 mM each | 0.5 |
| BSA | 1% in H_2_O | 1 |
| Forward Primer | 10 µM | 1.25 |
| Reverse Primer | 10 µM | 1.25 |
| PCR Buffer | 10x | 2.5 |
| DNA Template | .01-1x | 5 |
| ddH_2_O | — | 13.375 |
| Total | — | 25 |

PCR Program:

1. 95°C, 4 minutes
2. 95°C, 1 minute
3. 53°C, 1 minute
4. 72°C, 1 minute
5. Go to 2, repeat 2-4, 40 times
6. 72°C, 7 minutes
7. 4°C, ∞

*ITS1F-LR3:*

PCR Solution

| Component | Concentration | Volume (µL) |
| --- | --- | --- |
| EconoTaq | 5 U/µL | 0.125 |
| dNTPs | 10 mM each | 0.5 |
| BSA | 1% in H_2_O | 1 |
| Forward Primer | 10 µM | 1.25 |
| Reverse Primer | 10 µM | 1.25 |
| PCR Buffer | 10x | 2.5 |
| DNA Template | .01-1x | 5 |
| ddH_2_O | — | 13.375 |
| Total | — | 25 |

PCR Program:

1. 95°C, 5 minutes
2. 95°C, 1 minute
3. 53°C, 30 seconds
4. 72°C, 2 minutes
5. Go to 2, repeat 2-4, 35 times
6. 72°C, 10 minutes
7. 4°C, ∞

*LR0R-LR5:*

PCR Solution

| Component | Concentration | Volume (µL) |
| --- | --- | --- |
| EconoTaq | 5 U/µL | 0.125 |
| dNTPs | 10 mM each | 0.5 |
| BSA | 1% in H_2_O | 1 |
| Forward Primer | 10 µM | 1.25 |
| Reverse Primer | 10 µM | 1.25 |
| PCR Buffer | 10x | 2.5 |
| DNA Template | .01-1x | 5 |
| ddH_2_O | — | 13.375 |
| Total | — | 25 |

PCR Program:

1. 95°C, 4 minutes
2. 95°C, 1 minute
3. 53°C, 1 minute
4. 72°C, 1 minute 30 seconds
5. Go to 2, repeat 2-4, 40 times
6. 72°C, 7 minutes
7. 4°C, ∞

*ITS1F-LR5:*

PCR Solution

| Component | Concentration | Volume (µL) |
| --- | --- | --- |
| DNA Template | 1x | 1 |
| Forward Primer | 10 µM | 2.5 |
| Reverse Primer | 10 µM | 2.5 |
| ddH_2_O | — | 5.7 |
| REDExtract-N-Amp PCR ReadyMix | 1x | 13.3 |
| Total | — | 25 |

PCR Program:

1. 94°C, 3 minutes
2. 94°C, 1 minute
3. 50°C, 45 seconds
4. 72°C, 1 minute 30 seconds
5. Go to 2, repeat 2-4, 35 times
6. 72°C, 10 minutes
7. 4°C, ∞

*mrSSU1-mrSSU3R:*

PCR Solution

| Component | Concentration | Volume (µL) |
| --- | --- | --- |
| Q5 | 2 U/µL | 0.25 |
| dNTPs | 10 mM each | 0.5 |
| BSA | 1% in H_2_O | 1 |
| Forward Primer | 10 µM | 1.25 |
| Reverse Primer | 10 µM | 1.25 |
| PCR Buffer | 5x | 5 |
| DNA Template | .01-1x | 5 |
| ddH_2_O | — | 10.75 |
| Total | — | 25 |

PCR Program:

1. 98°C, 30 seconds
2. 98°C, 10 seconds
3. 62°C, 30 seconds
4. 72°C, 30 seconds
5. Go to 2, repeat 2-4, 35 times
6. 72°C, 2 minutes
7. 4°C, ∞

*fRPB2-5F-fRPB2-7cR:*

PCR Solution

| Component | Concentration | Volume (µL) |
| --- | --- | --- |
| Q5 | 2 U/µL | 0.25 |
| dNTPs | 10 mM each | 0.5 |
| Forward Primer | 10 µM | 1.25 |
| Reverse Primer | 10 µM | 1.25 |
| PCR Buffer | 5x | 5 |
| DNA Template | 1x | 5 |
| ddH_2_O | — | 11.75 |
| Total | — | 25 |

PCR Program:

1. 98°C, 30 seconds
2. 98°C, 10 seconds
3. 67.5°C, 30 seconds, -1°C/cycle
4. 72°C, 40 seconds
5. Go to 2, repeat 2-4, 10 times
6. 98°C, 10 seconds
7. 63°C, 30 seconds
8. 72°C, 40 seconds
9. Go to 2, repeat 6-8, 35 times
10. 72°C, 2 minutes
11. 4°C, ∞

*fRPB2-7cF-fRPB2-11aR:*

PCR Solution

| Component | Concentration | Volume (µL) |
| --- | --- | --- |
| Q5 | 2 U/µL | 0.25 |
| dNTPs | 10 mM each | 0.5 |
| Forward Primer | 10 µM | 1.25 |
| Reverse Primer | 10 µM | 1.25 |
| PCR Buffer | 5x | 5 |
| DNA Template | 1x | 5 |
| ddH_2_O | — | 11.75 |
| Total | — | 25 |

PCR Program:

1. 98°C, 30 seconds
2. 98°C, 10 seconds
3. 72°C, 30 seconds, -1°C/cycle
4. 72°C, 40 seconds
5. Go to 2, repeat 2-4, 10 times
6. 98°C, 10 seconds
7. 67.5°C, 30 seconds
8. 72°C, 40 seconds
9. Go to 2, repeat 6-8, 35 times
10. 72°C, 2 minutes
11. 4°C, ∞
