# Supplementary material for: Sareomycetes: more diverse than meets the eye
Source: IMA Fungus. 2021 Mar 16;12:6. doi: 10.1186/s43008-021-00056-0 (PMC7961326; doi:10.1186/s43008-021-00056-0)
Supplement: Supplementary file 2 — Additional file 2: File S2. Full Specimen Citations. Full information for specimens examined, including fine locality data, host, collection date, and collector number. [file 43008_2021_56_MOESM2_ESM.docx]

***Atrozythia klamathica***: **USA**: *Washington*: Whatcom County, Baker Lake, Boulder Creek Campground on Baker Lake Road, 48°42'53” N 121°41'40” W, 287 m a.s.l., apothecia on resin on bole of *Tsuga heterophylla,* 12 Mar. 2018, *M. Haldeman* *2748* (hb. Haldeman).

***Sarea coeloplata***: **Austria**: *Styria*: Wald unter dem Schutzhaus am Stuhleck, auf Harz und Rinde auf Lärchenzweigen [*Larix* sp.], 29 Jun. 1900, *F.X.R. von Höhnel* (FH 0096533). – **Canada**: *Newfoundland and Labrador*: Newfoundland, Division No. 5, Mount Ignoble top, 48°59'55.5” N 57°45'08.7” W, 283 m a.s.l., on *Picea mariana* resin, 26 May 2018, *A. Voitk* *18.05.26.AV04* (VAL); ibid., *A. Voitk* *18.05.26.AV05* (VAL). *Prince Edward Island*: Kings County, Southampton Wildlife Management Area, 46°21'01.9” N 62°34'10.6” W, *Picea* resin, 3 Oct. 2014, *R.T. McMullin* *14963* (CANL); Prince County, Greenpark Provincial Park, 46°35'33.6” N 63°53'33.7” W, *Picea* resin, 30 Sep. 2014, *R.T. McMullin* *14565* (CANL 129898). *Quebec*: Lac Clair near Quebec [City?], on spruce [*Picea* sp.], Sep. 1888, *W.G. Farlow* (FH 00995487). *Yukon*: 60°48'13” N 137°26'03” W, 670 m a.s.l., on conifer exudate [probably *Picea* sp.], 7 Jun. 2011, *J.C. Lendemer* *29136* (NY 01575087). – **Cape Verde**: *Santo Antão*: Agua das Caldeiras, 17°06'58.91” N 25°04'10.68” W, 1387 m a.s.l., on *Pinus* cf. *nigra* resin, Aug. 2017, *I. Garrido-Benavent* *IGB457* (VAL). – **Europe**: *J.A.P. Hepp*, *Die Flechten Europas s.n.* (FH 00964656). – **France**: *Sarthe*: Bourg-le-Roi, sur la resine des pins [*Pinus* sp.], Aug. 1907, *E. Monguillon* *2092* (B 600006463). – **Germany**: *Baden-Württemberg*: Donnstetten auf der Württemberger Alp, an Fichten [*Picea* sp.], 1866, *C.A. Kemmler*, *L.G. Rabenhorst Lichenes Europaei* *786* (FH 00965340). *Hessen*: Bergstraße-Odenwald, Oberschönmattenwag, Ellenbachtal, 300 m a.s.l., *Picea*-Harz, 3 May 1953, *O. Behr* *6699* (B 600006464). *North Rhine-Westphalia*: Münster, zwischen Lütkenbeck u. Angelmotte [und Angelmodde], auf Wurzeln von Tannen [*Abies* or *Pinus* sp.], Jul. 1861 (B 600198626). – **Italy**: *Sondrio*: ad coniferarum truncos et ramos resina illinitos, *M. Anzi Lichenes Rariores Langobardi Exsiccati* *267B* (FH 00965341). *Vercelli*: Valsesia, presso Riva, nelle anfrattuosità e cicatrici resinose della corteccia delle conifere, 1863, *A. Carestia*, *Erbario Crittogamico Italiano 1166* (FH 00965351). – **Norway**: *Buskerud*: prope Drammen ad Gulskoven [= Gulskogen], *J.M. Norman* (TROM L-42692). *Hedmark*: Furuberget Quarry, 60°49'01.11” N 11°02'23.53” E, on resin of fallen *Pinus sylvestris* attacked by *Cronartium flaccidum* or *Peridermium pini*, 14 Sep. 2017, *P. Vetlesen* *PV-D836-B* (FH 00965387). – **Spain**: *Madrid*: San Lorenzo de el Escorial, Pinar de Abantos, 40°35'38.77” N 4°09'36.11” W, 1200 m a.s.l., on *Pinus pinaster* resin, 5 Jun. 2017, *I. Garrido-Benavent* *IGB454* (VAL). *Santa Cruz de Tenerife*: Tenerife, Lomo de la Jara, on *Pinus radiata* resin, 23 May 2019, *R.N. Piñero* *19052301* (VAL). *Soria*: Abejar, Playa Pita, 41°50'16.42” N 2°46'43.85” W, 1090 m a.s.l., on *Pinus sylvestris* resin, 16 Mar. 2014, *I. Garrido-Benavent* *IGB316* (VAL). *Valencia*: Barx, Pla de la Nevereta, 38°59'52.43” N 0°18'15.35” W, 677 m a.s.l., on *Pinus halapensis* resin, 4 Jan. 2018, *I. Garrido-Benavent* *IGB452* (VAL); Quatretonda, Pla de Mora, 38°59'58.67” N 0°22'34.71” W, 223 m a.s.l., on *Pinus halapensis* resin, 27 Dec. 2017, *I. Garrido-Benavent* *IGB448* (VAL); ibid., *I. Garrido-Benavent* *IGB451* (VAL). – **Switzerland**: *Grisons*: Davos, Davos Lake, 46°49'07.06” N 9°51'25.33” E, 1579 m a.s.l., on *Picea abies* resin, 18 Jun. 2018, *I. Garrido-Benavent* *IGB716* (VAL); ibid., *I. Garrido-Benavent* *IGB717* (VAL). *Wallis*: Unter Wallis, Va. d'Herens, S. of Sion. Arbey, c. 1 km W of Evolène, c. 46°06'36.0” N 7°29'02.1” E, c. 1450 m a.s.l., 26 Jul. 1990, *H. Sipman* 30286 (B 600080177). – **USA**: *Arkansas*: Faulkner County, Cove Creek Natural Area, 35°17'24” N 92°28'48” W, on *Pinus* resin, 7 Oct. 2010, *J.C. Lendemer, D. Ladd & C.A. Morse* *26230-A* (NY 01218604). *California*: Del Norte County, Jedediah Smith Redwoods State Park, 41°49'11.9” N 124°07'02.3” W, 35 m a.s.l., on *Tsuga heterophylla* resin, 13 Dec. 2017, *J.K. Mitchell* *JM0071* (FH); ibid., 41°48'44.3” N 124°06'32.3” W, 57 m a.s.l., on *Tsuga heterophylla* resin, 13 Dec. 2017, *J.K. Mitchell* *JM0072* (FH); ibid., Redwoods National Park, 41°32'05.6” N 124°04'16.0” W, 10 m a.s.l., on *Picea sitchensis* resin, 14 Dec. 2017, *J.K. Mitchell* *JM0073.8* (FH); El Dorado County, Eldorado National Forest, Placerville Ranger District Headquarters, 38°44'15.1” N 120°39'51.0” W, 985 m a.s.l., on *Pinus ponderosa* resin, 6 Dec. 2017, *J.K. Mitchell* *JM0047* (FH); ibid., *J.K. Mitchell* *JM0048* (FH); ibid., 38°44'10.1” N 120°39'52.1” W, 1022 m a.s.l., on *Pinus nigra* subsp. *laricio* resin, 6 Dec. 2017, *J.K. Mitchell* *JM0049.1* (FH); Humboldt County, Prairie Creek Redwoods State Park, 41°21'13.5” N 124°01'35.5” W, 32 m a.s.l., on *Picea sitchensis* resin, 14 Dec. 2017, *J.K. Mitchell* *JM0075.2* (FH); Nevada County, Tahoe National Forest, Supervisor's Office, 39°16'09.6” N 121°01'02.3” W, 784 m a.s.l., on *Pinus ponderosa* resin, 7 Dec. 2017, *J.K. Mitchell* *JM0055* (FH); Plumas County, Plumas National Forest, 39°42'26.7” N 121°11'39.8” W, 1060 m a.s.l., on *Pseudotsuga menziesii* var. *menziesii* resin, 8 Dec. 2017, *J.K. Mitchell* *JM0064.2* (FH); Sierra County, Tahoe National Forest, 39°31'10.1” N 121°00'03.1” W, 668 m a.s.l., on *Pseudotsuga menziesii* var. *menziesii* resin, 7 Dec. 2017, *J.K. Mitchell* *JM0060.2* (FH); ibid., 39°31'09.3” N 121°00'03.1” W, 668 m a.s.l., on *Pinus ponderosa* resin, 7 Dec. 2017, *J.K. Mitchell* *JM0061.2* (FH); Siskiyou County, Klamath National Forest, 41°50'04.5” N 123°25'35.0” W, 549 m, a.s.l., on *Pseudotsuga menziesii* var. *menziesii* resin, 12 Dec. 2017, *J.K. Mitchell* *JM0070.2* (FH 00965409); Yuba County, Tahoe National Forest, 39°24'08.0” N 121°04'46.2” W, 505 m a.s.l., on *Pseudotsuga menziesii* var. *menziesii* resin, 7 Dec. 2017, *J.K. Mitchell* *JM0057.2* (FH); ibid., 39°24'16.8” N 121°04'34.9” W, 524 m a.s.l., on *Pinus ponderosa* resin, 7 Dec. 2017, *J.K. Mitchell* *JM0058.2* (FH); ibid., 39°31'09.8” N 121°00'03.5” W, 668 m a.s.l., on *Pseudotsuga menziesii* var. *menziesii* resin, 7 Dec. 2017, *J.K. Mitchell* *JM0059* (FH). *Maine*: York County, York, on spruce [*Picea* sp.] roots, *R. Thaxter 1459* (FH 00965337); ibid., on spruce [*Picea* sp.] resin, 12 Aug. 1897, *R. Thaxter* (FH 00995492). *Massachusetts*: Bristol County, New Bedford, on white pine [*Pinus strobus*], 1883, *H. Willey* (FH 00979165); Essex County, Appleton Farms Grass Rides, 42°38'40.80” N 70°52'04.20” W, 21 May 2017, *E. Kneiper & J.K. Mitchell* *JM0003* (FH); ibid., Groveland, Aug. 1890, *W.G. Farlow* (FH 00445743); ibid., on *Pinus rigida*, Aug. 1890, *W.G. Farlow* (FH 00979164); Middlesex County, Concord, Estabrook Woods, 42°29'00.26” N 71°21'24.88” W, on *Pinus strobus* resin, 19 Sep. 2017, *J.K. Mitchell* *JM0017* (FH); ibid., 42°29'00.19” N 71°21'24.15” W, 67 m a.s.l., on resin of *Pinus strobus*, 25 Jan. 2020, *J.K. Mitchell & D.E.W. Adamec* *JM0132* (FH); Suffolk County, Arnold Arboretum, 42°17'55.34” N 71°07'33.38” W, on *Pinus tabuliformis* 16576N resin, 27 Jul. 2017, *J.K. Mitchell* *JM0011* (FH); ibid., 42°17'53.71” N 71°07'40.06” W, on *Picea glehnii* 16485-B resin, 8 Oct. 2017, *J.K. Mitchell & L. Quijada* *JM0020* (FH); ibid., Thompson Island, 42°18'44.69” N 71°00'39.81” W, on *Pinus nigra* resin, 17 May 2017, *L.A. Kappler & J.K. Mitchell* *BHI-F925* (FH); ibid., 42°18'44.96” N 71°00'40.93” W, on *Pinus nigra* resin*,* 17 May 2017, *L.A. Kappler & J.K. Mitchell* *BHI-F926* (FH); Worcester County, Devens Reserve Forces Training Area, 42°28'22.60” N 71°39'11.34” W, 87 m a.s.l., on hardened trunk resin of *Pinus rigida*, 10 Sep. 1998, *E. Kneiper* *K987694* (FH 00405294). *New Hampshire*: Carroll County, Tamworth, Chocorua, on pine [*Pinus* sp.] resin, 23 Sep. 1909, *W.G. Farlow* (FH 00979162); Hillsborough County, Antrim, Loveren's Mill Cedar Swamp Preserve, 43°14'22.96” N 72°01'28.19” W, 336 m a.s.l., on *Abies balsamea* resin, 10 Sep. 2018, *J.K. Mitchell & Luis Quijada* *JM0104.2* (FH). *New York*: Adirondacks, Essex County, Keene Valley, Sep. 1902, *W.G. Farlow* (FH 00995485). *Ohio*: Morgan County, Burr Oak State Park, 39°31'44.24” N 82°01'38.14” W, on *Pinus strobus* resin, 7 Oct. 2017, *T.J. Curtis* *JM-TJC01* (KE 5869). *Oregon*: Lane County, Eugene, Hendricks Park, on *Pseudotsuga* resin, 5 Aug. 1978, *M.A. Sherwood* (FH 00965334); ibid., H. J. Andrews Experimental Forest, 472 m a.s.l., 16 Mar. 1979, *M.A. Sherwood* (FH 00965336). *Wisconsin*: Door County, Whitefish Dunes State Park, 44°55'22.8” N 87°11'39.8” W, 190 m a.s.l., on resin of *Thuja occidentalis*, 10 May 2019, *A.C. Dirks* *ACD0147* (MICH 139996). *Vermont*: Washington County, Calais, Chickering Bog Natural Area, 44°19'48.00” N 72°28'17.60” W, on *Pinus* cf. *banksiana* resin, 21 Oct. 2017, *J.K. Mitchell & L. Quijada* *JM0024* (FH); ibid., 44°19'43.30” N 72°28'17.50” W, on *Pinus* cf. *banksiana* resin, 21 Oct. 2017, *J.K. Mitchell & L. Quijada* *JM0027* (FH); ibid., 44°19'28.80” N 72°28'24.40” W, on *Abies balsamea* resin, 21 Oct. 2017, *J.K. Mitchell & L. Quijada* *JM0029* (FH).

***Sarea difformis***: **Canada**: *British Columbia*: Calvert Island, 51°39'18.0” N 128°08'16.8” W, resinicolous, 18 Jun. 2018, *R.T. McMullin 19801* (CANL 132189). *Nova Scotia*: Halifax County, Old Annapolis Road Nature Reserve, 44°45'03.9” N 63°56'33.5” W, resinicolous, 25 Jun. 2017, *R.T. McMullin* *17350* (CANL). *Ontario*: Nipissing District, Algonquin Provincial Park, 45°54'08.5” N 77°53'13.1” W, *Picea* sp., 1 Sep. 2013, *R.T. McMullin* *12673* (CANL 132522). *Prince Edward Island*: Kings County, Dromore Wildlife Management Area, 46°18'30.3” N 62°49'47.8” W, *Picea* resin, 7 Oct. 2014, *R.T. McMullin* *14881* (CANL 129879); Queens County, Mount Stewart Wildlife Management Area, 46°22'55.9” N 62°51'40.0” W, *Picea* resin, 1 Oct. 2014, *R.T. McMullin* *14453* (CANL). – **Czechia**: *Central Bohemia*: Brdy Hills, Nepomuk, 49°40'02” N 13°49'05” E, 765 m a.s.l., on resin of *Picea abies,* 15 Aug. 2018, *J. Malíček & J. Vondrák* *12001* (hb. Malíček). *Plzen*: Srby, 49°31'21” N 13°34'25” E, 550 m a.s.l., on resin of *Picea abies*, 25 Oct. 2018, *J. Malíček & J. Vondrák* *12161* (hb. Malíček). – **Europe**: *J.A.P. Hepp,* *Die Flechten Europas s.n.* (FH 00964655). – **Italy**: *Vercelli*: Valsesia, presso Riva, nelle anfrattuosità e cicatrici resinose della corteccia delle conifere, 1863, *A. Carestia*, *Erbario Crittogamico Italiano 1166* (FH 00965351). – **Norway**: *Hedmark*: Furuberget Quarry, 60°49'01.11” N 11°02'23.53” E, on resin of fallen *Pinus sylvestris* attacked by *Cronartium flaccidum* or *Peridermium pini*, 14 Sep. 2017, *P. Vetlesen* *PV-D836* (FH 00965386). – **USA:** *Arkansas*: Faulkner County, Cove Creek Natural Area, 35°17'24” N 92°28'48” W, on *Pinus* resin, 7 Oct. 2010, *J.C. Lendemer, D. Ladd & C.A. Morse* *26230* (NY 01218605). *California*: Del Norte County, Jedediah Smith Redwoods State Park, 41°48'44.3” N 124°06'32.3” W, 57 m a.s.l., on *Tsuga heterophylla* resin, 13 Dec. 2017, *J.K. Mitchell* *JM0072* (FH); ibid., Redwoods National Park, 41°32'05.6” N 124°04'16.0” W, 10 m a.s.l., on *Picea sitchensis* resin, 14 Dec. 2017, *J.K. Mitchell* *JM0074.1* (FH); Plumas County, Plumas National Forest, 39°42'31.9” N 121°11'40.3” W, 1056 m a.s.l., on resin of *Pinus lambertiana* resin, 8 Dec. 2017, *J.K. Mitchell* *JM0065.2* (FH). *Georgia*: Douglas County, Sweetwater Creek State Park, 33°45'12.86” N 84°37'44.54” W, on *Pinus* cf. *taeda* resin, 21 Jul. 2017, *J.K. Mitchell & M. Barrios* *JM0010.1* (FH 00965390); White County, Unicoi State Park, 34°42'43.00” N 83°43'49.60” W, on *Pinus* sp. resin, 16 Jul. 2017, *J.K. Mitchell* *JM0009.2* (FH 00965389). *Indiana*: Monroe County, Morgan-Monroe State Forest, 39°18'16” N 86°23'24” W, 259 m a.s.l., on *Pinus strobus* resin, 13 Apr. 2017, *J.C. Lendemer* *51265* (NY 02795595); ibid., 39°17'56” N 86°23'37” W, 232 m a.s.l., on *Pinus strobus* resin, 13 Apr. 2017, *J.C. Lendemer 51272* (NY 02795588). *Maine*: Washington County, Eagle Hill Institute, 44°27'35.03” N 67°55'53.01” W, 5 m a.s.l., on *Picea rubens* resin, 22 May 2017, *E. Kneiper* *JMEK* (FH); ibid., 44°27'23.36” N 67°55'44.11” W, 51 m a.s.l., on *Pinus banksiana* resin, 28 May 2017, *J.M. Karakehian* *17052821F* (FH); ibid., 44°27'36.01” N 67°55'46.92” W, on *Picea* cf. *glauca* resin, 3 Jul. 2017, *J.K. Mitchell* *JM0007* (FH); ibid., 44°27'34.8” N 67°55'58.6” W, resinicolous on *Picea*, 7 Jun. 2018, *R.T. McMullin* *19157* (CANL); ibid., Machiasport, on living tree (fir [*Abies* sp.]), 26 Aug. 1898, *M.A. Barker* *44* (FH 00995486); York County, Kittery, Kittery Point, on resin of *Pinus strobus*, 5 Feb. 1887, *R. Thaxter* (FH 00979158); ibid., *R. Thaxter* *2886* (FH 00995497); ibid., York, on spruce [*Picea* sp.] roots, *R. Thaxter 1459* (FH 00965337); ibid., on resin of *Picea* sp., *R. Thaxter*, *Reliquiae Farlowianae 669* (FH 00995493); ibid., *R. Thaxter 5573* (FH 00995499). *Massachusetts*: Bristol County, New Bedford, on pine [*Pinus* sp.] gum, 1865, *H. Willey* (FH 00965345); ibid., on white pine [*Pinus strobus*], 1882, *H. Willey* *950* (FH 00965344); Essex County, Groveland, on *P*[*inus*] *rigida*, Aug. 1890, *W.G. Farlow* (FH 00979163); Middlesex County, Concord, Estabrook Woods, 42°28'59.96” N 71°21'24.97” W, on *Pinus strobus* resin, 19 September 2017, *J.K. Mitchell* *JM0015* (FH); Norfolk County, Blue Hills Reservation, on resin on bark, 18 Apr. 1993, *D.H. Pfister* (FH 00965333); Suffolk County, Boston, Arnold Arboretum, 42°17'55.53” N 71°07'31.63” W, on *Pinus strobus* 'Contorta' resin, 13 May 2017, *J.K. Mitchell* *JM0001* (FH); ibid., 42°17'55.34” N 71°07'33.38” W, on *Pinus tabuliformis* 16576N resin, 27 Jul. 2017, *J.K. Mitchell* *JM0011* (FH); Worcester County, Petersham, Harvard Forest, 42°32'15.03” N 72°10'58.94” W, on *Picea mariana* resin, 13 May 2018, *J.K. Mitchell & L. Quijada* *JM0082* (FH). *Minnesota*: Isanti County, Cedar Creek Ecosystem Science Reserve, 45°25'15.39” N 93°11'48.88” W, 292 m a.s.l., on *Pinus strobus* resin, 11 Aug. 2019, *J.K. Mitchell* *JM0108* (FH 00965393). *New Hampshire*: Carroll County, Intervale, *R. Thaxter* (FH 00979159); ibid., Tamworth, Chocorua, Sep. 1907, *W.G. Farlow* (FH 00995494); ibid., on *P*[*inus*] *strobus*, Aug. 1910, *W.G. Farlow* (FH 00979161); Coos County, Randolph, on fir [*Abies* sp.] gum, 1885, *H. Willey* *1015* (FH 00979157); ibid., *H. Willey 1015* (FH 00965342); ibid., Shelburne, Sep. 1891, *W.G. Farlow* (FH 00995490); ibid., White Mountains National Forest, Tuckerman Ravine Trail, 44°15'41.45” N 71°16'02.38” W, 882 m a.s.l., on *Abies balsamea* resin, 16 Jun. 2018, *J.K. Mitchell* *JM0091* (FH 00965398); ibid., 44°15'49.75” N 71°16'40.29” W, 1049 m a.s.l., on *Picea rubens* resin, 16 Jun. 2018, *J.K. Mitchell* *JM0092* (FH 00965399). *North Carolina*: Swain County, Great Smoky Mountains National Park, 35°32'25-33'17” N 83°29'36"-44” W, 1768-1859 m a.s.l., 10 Oct. 2011, *E.A. Tripp & J.C. Lendemer* *2261* (NY 01685454). *Tennessee*: Sevier County, Great Smoky Mountains National Park, Boulevard Trail, 35°38'03” N 83°24'50” W, 1814 m a.s.l., 7 Aug. 2012, *E.A. Tripp & J.C. Lendemer* *3446* (NY 01685081); ibid., Bullhead Trail, 35°39'36"-40'32” N 83°27'02"-29'08” W, on *Picea* sap, 9 Oct. 2011, *J.C. Lendemer, E.A. Tripp & E. Darling* *30379* (NY 01237252); ibid., Sugarland Mountain Trail, resinicolous on *Picea*, 26 Oct. 2017, *R.T. McMullin* *19017* (NY 03303142). *Vermont*: Washington County, Calais, Chickering Bog Natural Area, 44°19'26.30” N 72°28'39.20” W, on *Picea* sp. resin, 21 Oct. 2017, *J.K. Mitchell & L. Quijada* *JM0031* (FH); ibid., 44°19'31.30” N 72°28'48.30” W, on *Larix laricina* resin, 21 Oct. 2017, *J.K. Mitchell & L. Quijada* *JM0032* (FH).

***Zythia resinae***: **Cape Verde**: *Santiago*: São Miguel, Serra Malagueta, 15°10'46.99” N 23°40'21.11” W, 1029 m a.s.l., on *Pinus canariensis* resin, 29 Jul. 2017, *I. Garrido-Benavent IGB456* (VAL). – **China**: *Heilongjiang*: Jixi, Hulin, Dōngfāng hóng, on *Pinus koraiensis* resin, 4 Sep. 1986, *T. Kobayashi & J.-Z. Zhao* *FPH-6930* (TFM); Mudanjiang, Ning'an, Dōngjīng zhèn, on *Pinus koraiensis* resin, 11 Sep. 1986, *T. Kobayashi & J.-Z. Zhao* *FPH-6932* (TFM); ibid., Jiangshanjiao Experimental Forest Farm, on *Pinus koraiensis* resin, 9 Sep. 1986, *T. Kobayashi* *FPH-6926* (TFM); Qitaihe, Boli, on *Pinus koraiensis* resin, 15 Sep. 1986, *T. Kobayashi & J.-Z. Zhao* *FPH-6931* (TFM). *Yunnan*: Lijiang County, Lijiang, Elephant Mountain, 26°53'18” N 100°14'12” E, 2400 m a.s.l., on resinous trunk of *Pinus* sp., 20 Oct. 2002, *H. Sipman* *49954* (B 600202098); ibid., 26°53'13” N 100°14'05” E, 2550 m a.s.l., on *Pinus* sp. resin, 20 Oct. 2002, *A. Aptroot* *56089* (DUKE 0133124). – **Czechia**: *Central Bohemia*: Brdy Hills, 49°44'52” N 13°56'44” E, 650 m a.s.l., on resin of *Larix decidua*, 30 Aug. 2018, *J. Malíček & J. Vondrák* *12020* (hb. Malíček); ibid., Jince, 49°45'44” N 13°56'21” E, 580 m a.s.l., on resin of *Larix decidua*, 27 Aug. 2018, *J. Malíček & J. Vondrák* *12018* (hb. Malíček); ibid., Nepomuk, 49°40'06” N 13°49'34” E, 730 m a.s.l., on resin of *Picea abies*, 15 Aug. 2018, *J. Malíček & J. Vondrák* *12005* (hb. Malíček); ibid., Strasice, 49°43'34” N 13°47'56” E, 610 m a.s.l., on resin of *Larix decidua*, 20 Aug. 2018, *J. Malíček & J. Vondrák* *11998* (hb. Malíček). *Plzen*: Srby, 49°31'21” N 13°34'25” E, 550 m a.s.l., on resin of *Larix decidua*, 25 Oct. 2018, *J. Malíček & J. Vondrák* *12159* (hb. Malíček). – **Dominican Republic**: *La Vega Province*: Parque Nacional Juan B. Perez, on resin of *Pinus occidentalis*, 7 Jan. 2002, *S. Cantrell, T. Iturriaga, J. Lodge, D.H. Pfister & M. de la Cruz* *DR-56* (FH 00965385). – **Japan**: *Ibaraki Prefecture*: Naka-gun, Hitachiota-shi, Mchiya, on *Pinus* bark and resin, 9 Nov. 2002, *T. Hosoya* *THX-134* (TNS-F-41522). – **Norway**: *Hedmark*: Furuberget Quarry, 60°49'01.11” N 11°02'23.53” E, on resin of fallen *Pinus sylvestris* attacked by *Cronartium flaccidum* or *Peridermium pini*, 14 Sep. 2017, *P. Vetlesen* *PV-D836-B* (FH 00965387). – **Spain**: *Santa Cruz de Tenerife*: Tenerife, Los Revolcaderos, on *Pinus radiata* resin, 16 Dec. 2017, *R.N. Piñero* *17121601* (VAL); ibid., 11 Apr. 2018, *R.N. Piñero* *18041101* (VAL). *Soria*: Abejar, Playa Pita, 41°50'16.42” N 2°46'43.85” W, 1090 m a.s.l., on *Pinus sylvestris* resin, 16 Mar. 2014, *I. Garrido-Benavent* *IGB316* (VAL). *Valencia*: Barx, Pla de la Nevereta, 38°59'52.43” N 0°18'15.35” W, 677 m a.s.l., on *Pinus halapensis* resin, 4 Jan. 2018, *I. Garrido-Benavent* *IGB453* (VAL); Quatretonda, 38°57'46.35” N 0°22'31.18” W, 367 m a.s.l., on *Cupressus arizonica* resin, 20 Aug. 2013, *I. Garrido-Benavent IGB317* (VAL); ibid., Pla de Mora, 38°59'58.67” N 0°22'34.71” W, 223 m a.s.l., on *Pinus halapensis* resin, 27 Dec. 2017, *I. Garrido-Benavent* *IGB449* (VAL); ibid., on *Cupressus sempervirens* resin, 27 Dec. 2017, *I. Garrido-Benavent* *IGB450* (VAL). – **USA**: *Arizona*: Coconino County, San Francisco Peaks, 35°21’ N 111°41’ W, 3450 m a.s.l., on *Pinus aristata* resinous bark, 12 Jun. 1998, *M. Westberg* *851* (LD 1356193). *California*: Del Norte County, Redwoods National Park, 41°32'05.6” N 124°04'16.0” W, 10 m a.s.l., on *Picea sitchensis* resin, 14 Dec. 2017, *J.K. Mitchell* *JM0074.2* (FH); Plumas County, Plumas National Forest, 39°42'26.7” N 121°11'39.8” W, 1060 m a.s.l., on *Pseudotsuga menziesii* resin, 8 Dec. 2017, *J.K. Mitchell* *JM0064.1* (FH); ibid., 39°42'31.9” N 121°11'40.3” W, 1056 m a.s.l., on *Pinus lambertiana* resin, 8 Dec. 2017, *J.K. Mitchell* *JM0065.1* (FH); San Diego County, Cleveland National Forest, 32°51'13.1” N 116°34'40.5” W, 1170 m a.s.l., on *Cupressus forbesii* bark and resin, 27 Dec. 2017, *J.K. Mitchell & M.D. Mitchell* *JM0077* (FH); Siskiyou County, Klamath National Forest, 41°50'03.6” N 123°25'42.1” W, 566 m a.s.l., on *Chamaecyparis lawsoniana* resin, 12 Dec. 2017, *J.K. Mitchell* *JM0068* (FH 00965406). *Georgia*: Douglas County, Sweetwater Creek State Park, 33°45'12.86” N 84°37'44.54” W, on *Pinus* cf. *taeda* resin, 21 Jul. 2017, *J.K. Mitchell & M. Barrios* *JM0010.2* (FH 00965391); White County, Unicoi State Park, 34°42'43.00” N 83°43'49.60” W, on *Pinus* sp. resin, 16 Jul. 2017, *J.K. Mitchell* *JM0009.1* (FH 00965388). *Idaho*: Clearwater County, 2 km NE of Southwick, 46°37'20.42” N 116°27'05.98” W, 785 m a.s.l., on resin on bark of bole of *Pseudotsuga menziesii*, 26 Aug. 2017, *M. Haldeman* *2514* (hb. Haldeman). *Maine*: Lincoln County, Southport, Pratts Island, on resin of *Picea*, 19 Feb. 1989, *D.H. Pfister* (FH 00965332); Washington County, Eagle Hill Institute, 44°27'36.00” N 67°55'49.40” W, on *Picea* cf. *glauca* resin, 3 Jul. 2017, *J.K. Mitchell* *JM0006* (FH); ibid., Milbridge, 44°32'24.10” N 67°52'52.60” W, on *Picea glauca* resin, 6 Jul. 2017, *J.K. Mitchell* *JM0008* (FH). *Massachusetts*: Barnstable County, Cape Cod National Seashore, Marconi Beach, 41°54'41.37” N 69°58'49.03” W, 9 m a.s.l., on resin of *Chamaecyparis thyoides*, 18 Oct. 2019, *J.K. Mitchell & D.E.W. Adamec* *JM0120* (FH); ibid., on *Chamaecyparis thyoides* canker, 15 Oct. 2011, *J.M. Karakehian* *11101502* (FH); Essex County, Appleton Farms Grass Rides, 42°38'30.10” N 70°51'49.30” W, on *Pinus* sp. resin, 21 May 2017, *E. Kneiper & J.K. Mitchell* *JM0004* (FH); Middlesex County, Concord, Estabrook Woods, 42°29'00.15” N 71°21'23.47” W, 67 m a.s.l., on resin of *Pinus strobus*, 25 Jan. 2020, *J.K. Mitchell & D.E.W. Adamec* *JM0131* (FH); Norfolk County, Blue Hills Reservation, on resin on bark, 18 Apr. 1993, *D.H. Pfister* (FH 00965333); ibid., Webb Memorial State Park, 42°15'29.58” N 70°55'22.62” W, 1 m a.s.l., on resin of live *Pinus nigra* tree, 29 Mar. 2017, *A.C. Dirks & J.K. Mitchell* *BHI-F779* (FH); Plymouth County, Grape Island, 42°16'15.67” N 70°55'07.43” W, on resin flow of *Pinus strobus* tree, 3 May 2017, *L.A. Kappler & J.K. Mitchell* *BHI-F871* (FH); Suffolk County, Arnold Arboretum, 42°17'55.49” N 71°07'33.83” W, on *Pinus sylvestris* 438-57-B resin, 27 Jul. 2017, *J.K. Mitchell* *JM0012* (FH); ibid., 42°17'54.93” N 71°07'29.95” W, on *Chamaecyparis obtusa* resin, 30 Oct. 2017, *J.K. Mitchell & L. Quijada* *JM0036* (FH); Worcester County, Devens Reserve Forces Training Area, 42°28'22.60” N 71°39'11.34” W, 87 m a.s.l., on hardened trunk resin of *Pinus rigida*, 10 Sep. 1998, *E. Kneiper* *K987694* (FH 00405294); ibid., Petersham, Harvard Forest, 42°32'09.37” N 72°11'16.15” W, on resin of a live *Pinus strobus* tree, 18 Aug. 2017, *J.K. Mitchell* *JM0014* (FH); ibid., Princeton, Mass Audubon's Wachusett Meadow Wildlife Sanctuary, 42°27'20.1” N 71°54'18.7” W, 312 m a.s.l., on resin of planted *Juniperus virginiana*, 28 Dec. 2019, *J.K. Mitchell* *JM0125* (FH). *Michigan*: Washtenaw County, Ann Arbor, University of Michigan North Campus, 42°17'43.8” N 83°43'29.9” W, 289 m a.s.l., on resin of *Pinus sylvestris*, 9 Nov. 2019, *A.C. Dirks* *ACD0229* (MICH 139997). *Minnesota*: Isanti County, Cedar Creek Ecosystem Science Reserve, 45°25'15.39” N 93°11'48.88” W, 292 m a.s.l., on resin of *Pinus strobus*, 11 Aug. 2019, *J.K. Mitchell* *JM0107* (FH 00965392). *North Carolina*: Camden County, North River Game Land, 36°21'24” N 76°13'06” W, 0 m a.s.l., on *Taxodium* exudate, 12 Apr. 2012, *B. P. Hodkinson, J. Allen, R. C. Harris & J. C. Lendemer* *18239* (NY 01886893); Onslow County, Jacksonville, on *Juniperus scopulorum* ‘SkyRocket’ resinous wound, 7 Apr. 2006, *J. Morton* (NCSLG 17391). *Oregon*: Lane County, Eugene, Hendricks Park, on *Pseudotsuga* resin, 5 Aug. 1978, *M.A. Sherwood* (FH 00965334). *Rhode Island*: Washington County, Ell Pond Preserve, 41°30'22.00” N 71°46'46.66” W, on *Chamaecyparis thyoides* resin, 26 Nov. 2017, *J.K. Mitchell & L. Quijada* *JM0044* (FH). *Washington*: Whatcom County, Baker Lake, 48°42'53” N 121°41'40” W, 287 m a.s.l., on resin on bole of 71 cm diameter *Pseudotsuga menziesii*, 12 Mar. 2018, *M. Haldeman* *2747* (hb. Haldeman). *Wisconsin*: Dane County, Mazomanie Bottoms State Natural Area, 43°13'34.7” N 89°48'14.0” W, 225 m a.s.l., on resin of *Pinus* sp., 4 May 2019, *A.C. Dirks* *ACD0083* (MICH 139995); Door County, Whitefish Dunes State Park, 44°55'22.8” N 87°11'39.8” W, 190 m a.s.l., on resin of *Thuja occidentalis*, 10 May 2019, *A.C. Dirks* *ACD0147* (MICH 139996).
