# Supplementary material for: Sareomycetes: more diverse than meets the eye
Source: IMA Fungus. 2021 Mar 16;12:6. doi: 10.1186/s43008-021-00056-0 (PMC7961326; doi:10.1186/s43008-021-00056-0)
Supplement: Supplementary file 4 — Additional file 4: Table S2. Tests for strict molecular clock. Test for strict molecular clock for each locus conducted in MEGA 5 prior to performing the three-locus dating analyses (see section “Inferring a Time Frame for The Diversification of Sareomycetes” in Materials and Methods). Tested under two different topologies (ML and Bayesian). *denotes rejection of the null hypothesis (i.e., equal rates). [file 43008_2021_56_MOESM4_ESM.docx]

|  | | **ML estimate** | | | | | **MrBayes consensus** | | | | |
| --- | --- | --- | --- | --- | --- | --- | --- | --- | --- | --- | --- |
| **nrITS K2+Γ+I** | | | **ln*L*** | **Param** | **(+Γ)** | **(+I)** | | **ln*L*** | **Param** | **(+Γ)** | **(+I)** |
| With Clock | | | -2936.883 | 86 | 0.755 | 0.60 | | -2907.169 | 86 | 0.564 | 0.37 |
| Without Clock  P (Ho: = rates) |  | | -2799.163 | 168 | 0.75 | 0.52 | | -2685.295 | 168 | 0.66 | 0.44 |
|  |  | | 1.15e^-7^* | | | | | 1.28e^-27^* | | | |
| **nuLSU K2+Γ+I** | | | **ln*L*** | **Param** | **(+Γ)** | **(+I)** | | **ln*L*** | **Param** | **(+Γ)** | **(+I)** |
| With Clock | | | -2630.479 | 87 | 0.145 | 0.74 | | -2502.123 | 87 | 0.316 | 0.83 |
| Without Clock | | | -2282.383 | 170 | 0.70 | 0.82 | | -2399.594 | 170 | 0.31 | 0.83 |
| P (Ho: = rates) | | | 4.81e^-66^* | | | | | 0.02* | | | |
| **mtSSU HKY+Γ** | | | **ln*L*** | **Param** | **(+Γ)** |  | | **ln*L*** | **Param** | **(+Γ)** |  |
| With Clock | | | -2469.963 | 71 | 0.219 |  | | -2441.517 | 71 | 0.180 |  |
| Without Clock | | | -2377.089 | 136 | 0.20 |  | | -2380.866 | 136 | 0.20 |  |
| P (Ho: = rates) | | | 0.001* | | | | | 0.695 | | | |
