# Supplementary material for: Sareomycetes: more diverse than meets the eye
Source: IMA Fungus. 2021 Mar 16;12:6. doi: 10.1186/s43008-021-00056-0 (PMC7961326; doi:10.1186/s43008-021-00056-0)
Supplement: Supplementary file 6 — Additional file 6: Table S4. Calibration fossils. List of the six fossil calibrations used to estimate the age of the crown node of Sareomycetes with BEAST based on a six-locus dataset with 169 different fungal taxa. (Ma: million years ago). [file 43008_2021_56_MOESM6_ESM.docx]

| Calibration | | Evidence | Age (Ma) | Geological Time | Prior distribution and settings | References |
| --- | --- | --- | --- | --- | --- | --- |
| A | Crown of family *Parmeliaceae* | *Anzia electra* Rikkinen & Poinar is a lichenized fungus that was found in Baltic amber | 55–35 | Early Oligocene to Late Eocene | Exponential (mean= 45, offset= 40; initial: 43) | Rikkinen and Poinar 2002; Lücking and Nelsen 2018 |
| B | *Cyphelium-Calicium* clade | *Calicium* sp. fossil |  |  | Exponential (mean= 40, offset= 35; initial: 37) | Rikkinen 2003; Pérez-Ortega et al. 2016 |
| C | Crown of class *Coniocybomycetes* | *Chaenotheca* sp. fossil from Baltic amber |  |  | Exponential (mean= 98.9, offset= 35; initial: 70) | Rikkinen 2003; Pérez-Ortega et al. 2016 |
| D | Crown of family *Aspergillaceae* | *Aspergillus collembolorum* Dörfelt & A.R. Schmidt was found overgrowing a springtail (suborder *Entomobryomorpha*) in Baltic amber |  |  | Exponential (mean= 40, offset= 35; initial: 37) | Dörfelt and Schmidt 2005; Lutzoni et al. 2018; Samarakoon et al. 2019 |
| E | Common ancestor of *Capnodiales* | *Metacapnodium succinum* (Dörfelt, A.R. Schmidt & J. Wunderl.) Rikkinen, Dörfelt, A.R.  Schmidt & J. Wunderl. | ~100 (minimum age of 100 Mya for the crown age of  *Capnodiales*) |  | truncated normal distribution  mean = 100, standard deviation = 150, confidence interval  = 400; truncated upper = infinit; lower: 100 | Schmidt et al. 2014; Samarakoon et al. 2019 |
| F | Crown of *Pezizomycotina* | *Paleopyrenomycites devonicus* fossil |  |  | Exponential (mean= 67.8, offset= 400; initial: 400) | Taylor et al. 2005; Prieto and Wedin 2013; Beimforde et al. 2014; Pérez-Ortega et al. 2016; Samarakoon et al. 2019 |

**References:**

Beimforde C, Feldberg K, Nylinder S, et al (2014) Estimating the Phanerozoic history of the *Ascomycota* lineages: combining fossil and molecular data. Molecular Phylogenetics and Evolution 78:386–398. https://doi.org/10.1016/j.ympev.2014.04.024

Dörfelt H, Schmidt AR (2005) A fossil *Aspergillus* from Baltic amber. Mycological Research 109(8):956–960. https://doi.org/10.1017/S0953756205003497

Lücking R, Nelsen MP (2018) Ediacarans, Protolichens, and Lichen-Derived *Penicillium*. In: Krings M, Harper CJ, Cuneo NR, Rothwell GW (eds) Transformative Paleobotany: Papers to Commemorate the Life and Legacy of Thomas N. Taylor. San Diego: Elsevier Science & Technology, pp 551–590

Lutzoni F, Nowak MD, Alfaro ME, et al (2018) Contemporaneous radiations of fungi and plants linked to symbiosis. Nature Communications 9:5451. https://doi.org/10.1038/s41467-018-07849-9

Pérez-Ortega S, Garrido-Benavent I, Grube M, et al (2016) Hidden diversity of marine borderline lichens and a new order of fungi: *Collemopsidiales* (*Dothideomyceta*). Fungal Diversity 80(1):285–300. https://doi.org/10.1007/s13225-016-0361-1

Prieto M, Wedin M (2013) Dating the diversification of the major lineages of *Ascomycota* (*Fungi*). PLoS ONE 8(6):e65576. https://doi.org/10.1371/journal.pone.0065576

Rikkinen J (2003) Calicioid lichens from European Tertiary amber. Mycologia 95(6):1032–1036. https://doi.org/10.1080/15572536.2004.11833019

Rikkinen J, Poinar GO (2002) Fossilised *Anzia* (*Lecanorales*, lichen-forming *Ascomycota*) from European Tertiary amber. Mycological Research 106(8):984–990. https://doi.org/10.1017/S0953756202005907

Samarakoon MC, Hyde KD, Hongsanan S, et al (2019) Divergence time calibrations for ancient lineages of *Ascomycota* classification based on a modern review of estimations. Fungal Diversity 96(1):285–346. https://doi.org/10.1007/s13225-019-00423-8

Schmidt AR, Beimforde C, Seyfullah LJ, et al (2014) Amber fossils of sooty moulds. Review of Palaeobotany and Palynology 200:53–64. https://doi.org/10.1016/j.revpalbo.2013.07.002

Taylor TN, Hass H, Kerp H, et al (2005) Perithecial ascomycetes from the 400 million year old Rhynie chert: an example of ancestral polymorphism. Mycologia 97(1):269–285. https://doi.org/10.1080/15572536.2006.11832862
