# Supplementary material for: Sareomycetes: more diverse than meets the eye
Source: IMA Fungus. 2021 Mar 16;12:6. doi: 10.1186/s43008-021-00056-0 (PMC7961326; doi:10.1186/s43008-021-00056-0)
Supplement: Supplementary file 7 — Additional file 7: Table S5. Divergence time estimates of lineages in Sareomycetes. Divergence time estimates (Ma) of Sareomycetes and the main lineages within obtained using five different secondary calibration approaches with BEAST. The median (in millions of years, Ma) and 95% HPD intervals (in brackets) are given for each divergence time estimate. For simplicity, the “Epochs” interval for each row is based on the five median estimates and it does not consider the corresponding 95% HPD intervals. [file 43008_2021_56_MOESM7_ESM.docx]

|  | Calibrated node with Age estimate | mtSSU subst. rate | nuLSU subst. rate | *Erysiphales* nuITS subst. rate | *Melanohalea* nuITS subst. rate | Epochs |
| --- | --- | --- | --- | --- | --- | --- |
| *Sareomycetes* crown node | 115.77 (164.75–73.62) | 149.37 (216.5–94.19) | 114.81 (181.16–67.04) | 72.87 (107.99–47.87) | 53.1 (78.55–34.31) | Upp. Jurassic–Eocene |
| *Zythia* crown node | 35.07 (57.32–17.76) | 46.94 (68.7–29.06) | 37.08 (56.05–21.99) | 23.18 (33.56–14.96) | 17.17 (24.68–11.04) | Eocene–Miocene |
| *Atrozythia* crown node | 17.43 (36.66–4.87) | 23.56 (46.85–7.57) | 18.25 (37.92–6.04) | 11.66 (22.5–4.41) | 8.38 (16.47–3.18) | Oligocene–Miocene |
| *Sarea* crown node | 39.86 (67.66–21.28) | 54.6 (81.94–34.04) | 41.77 (68.81–22.7) | 26.24 (40.35–15.78) | 19.38 (29.25–11.6) | Eocene–Miocene |
| *Zythia*-*Atrozythia* split | 77.69 (118.81–43.95) | 100.86 (150.32–63.85) | 78.54 (125.67–45.89) | 49.81 (74.41–31.71) | 36.49 (53.68–22.89) | Low. Cretaceous–Eocene |
| *A. klamathica*-*A. lignicola* split | 17.43 (36.66–4.87) | 23.56 (46.85–7.57) | 18.25 (37.92–6.04) | 11.66 (22.5–4.41) | 8.38 (16.47–3.18) | Oligocene–Miocene |
| *S. difformis* crown | 17.04 (29.97–8.49) | 23.91 (37.03–14.16) | 18.01 (30.74–8.95) | 11.4 (18.23–6.42) | 8.39 (13.11–4.58) | Oligocene–Miocene |
| *S. coeloplata* 1 crown | 9.7 (19.12–4.09) | 13.14 (22.88–6.37) | 10.06 (18.77–4.3) | 6.37 (11.29–3.02) | 4.69 (8.25–2.25) | Miocene–Pliocene |
| *S. coeloplata* 2 crown | 11.27 (20.99–4.4) | 15.28 (25.81–7.52) | 11.98 (21.41–5.73) | 7.46 (12.72–3.73) | 5.48 (9.38–2.77) | Miocene |
