# Supplementary figures and images for: Sareomycetes: more diverse than meets the eye
Source: IMA Fungus. 2021 Mar 16;12:6. doi: 10.1186/s43008-021-00056-0 (PMC7961326; doi:10.1186/s43008-021-00056-0)

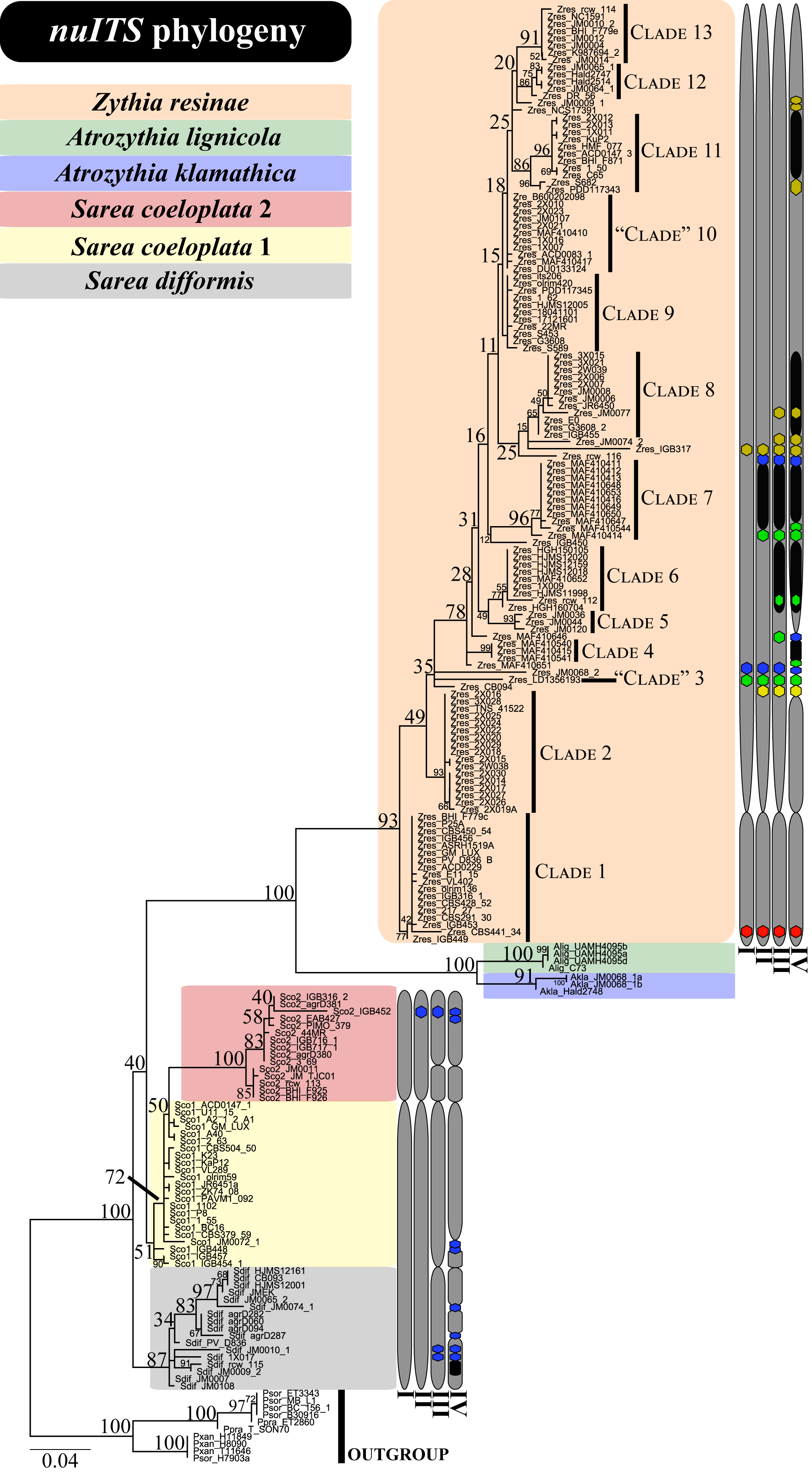

Supplement: Supplementary file 10 — Additional file 10: Figure S1. Sareomycetes nuITS phylogram and species delimitation scenarios based on ABGD. Maximum likelihood tree reconstruction obtained with RAxML based on nuITS data that depicts phylogenetic relationships among the studied Sareomycetes specimens. The voucher code of each sample is provided. Coloured boxes delineate the different taxa (genus, species) considered in the present study; full Latin names are available in the legend on the upper-left corner. Bootstrap support values are shown for each node. On the right margin of Zythia, species delimitation schemes are based on ABGD 6 (column I), 10 (II), 15 (III), and 24 (IV) putative species solutions. On the right margin of Sarea, the schemes are based on ABGD 2 (column I), 3 (II), 7 (III), and 16 (IV) putative species solutions. [file 43008_2021_56_MOESM10_ESM.tif]

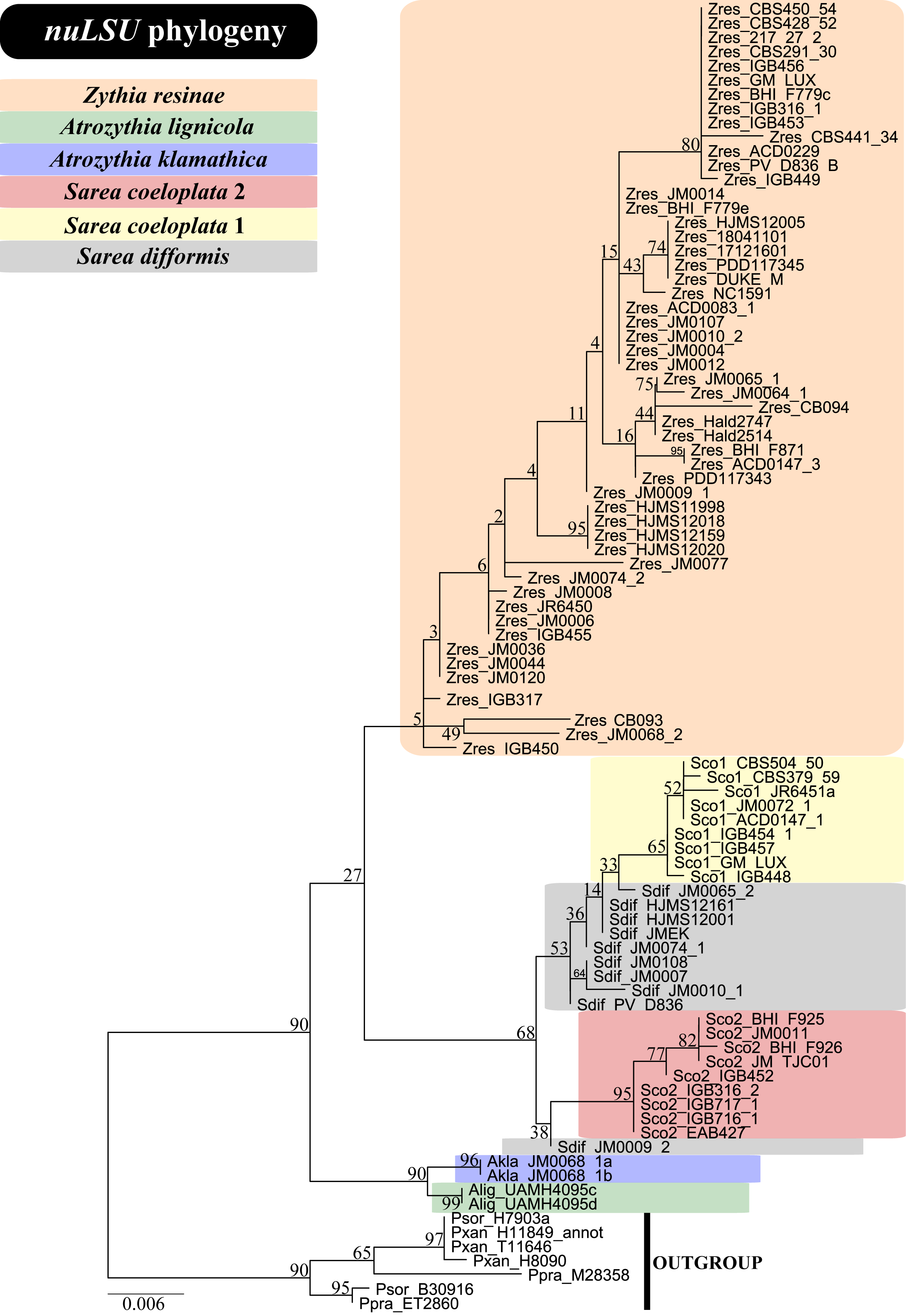

Supplement: Supplementary file 11 — Additional file 11: Figure S2. Sareomycetes nuLSU phylogram. Maximum likelihood tree reconstruction obtained with RAxML based on nuLSU data that depicts phylogenetic relationships among the studied Sareomycetes specimens. The voucher code of each sample is provided. Coloured boxes delineate the different taxa (genus, species) considered in the present study; full Latin names are available in the legend on the upper-left corner. Bootstrap support values are shown for each node. [file 43008_2021_56_MOESM11_ESM.tif]

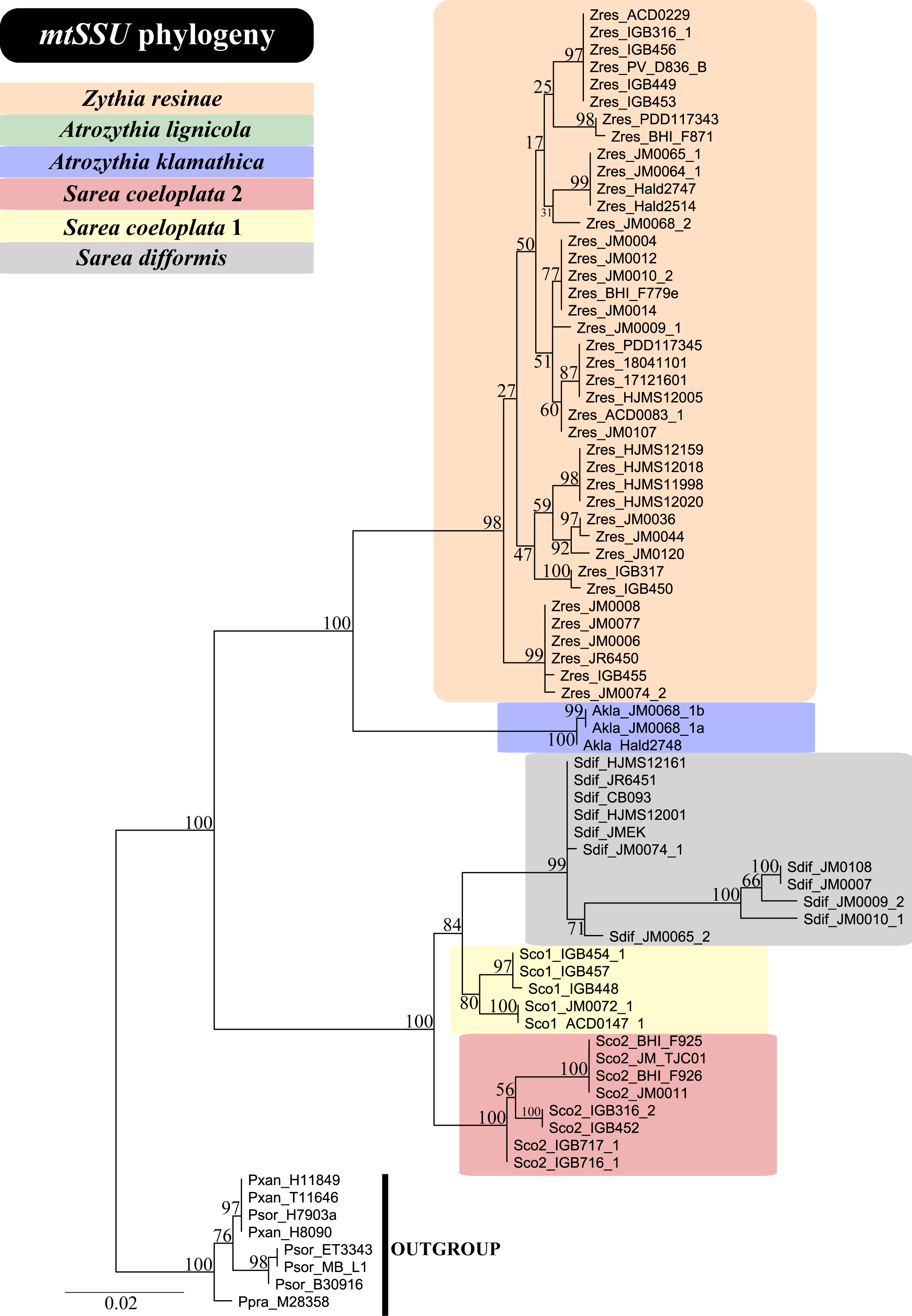

Supplement: Supplementary file 12 — Additional file 12: Figure S3. Sareomycetes mtSSU phylogram. Maximum likelihood tree reconstruction obtained with RAxML based on mtSSU data that depicts phylogenetic relationships among the studied Sareomycetes specimens. The voucher code of each sample is provided. Coloured boxes delineate the different taxa (genus, species) considered in the present study; full Latin names are available in the legend on the upper-left corner. Bootstrap support values are shown for each node. [file 43008_2021_56_MOESM12_ESM.tif]

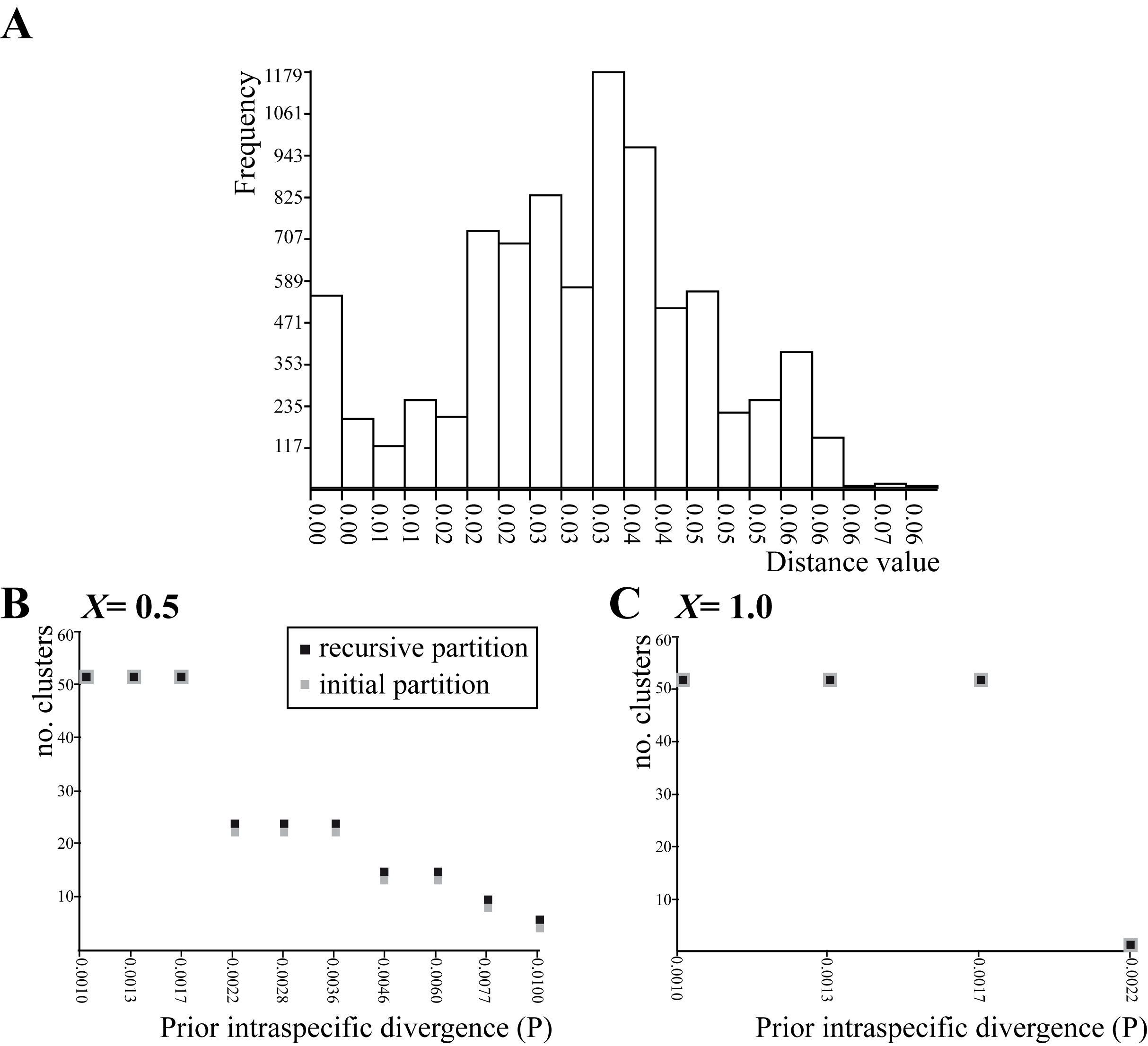

Supplement: Supplementary file 13 — Additional file 13: Figure S4. ABGD results for species delimitation in Zythia. A Histogram showing the distribution of pairwise genetic distances (K2P) among sequences (specimens). B–C Graphs showing the inferred number of clusters (i.e., ABGD partitions or putative species) with different Prior intraspecific divergence (P) values. Analyses in B and C used a value for the relative gap width (X) of 0.5 and 1.0, respectively. [file 43008_2021_56_MOESM13_ESM.tif]

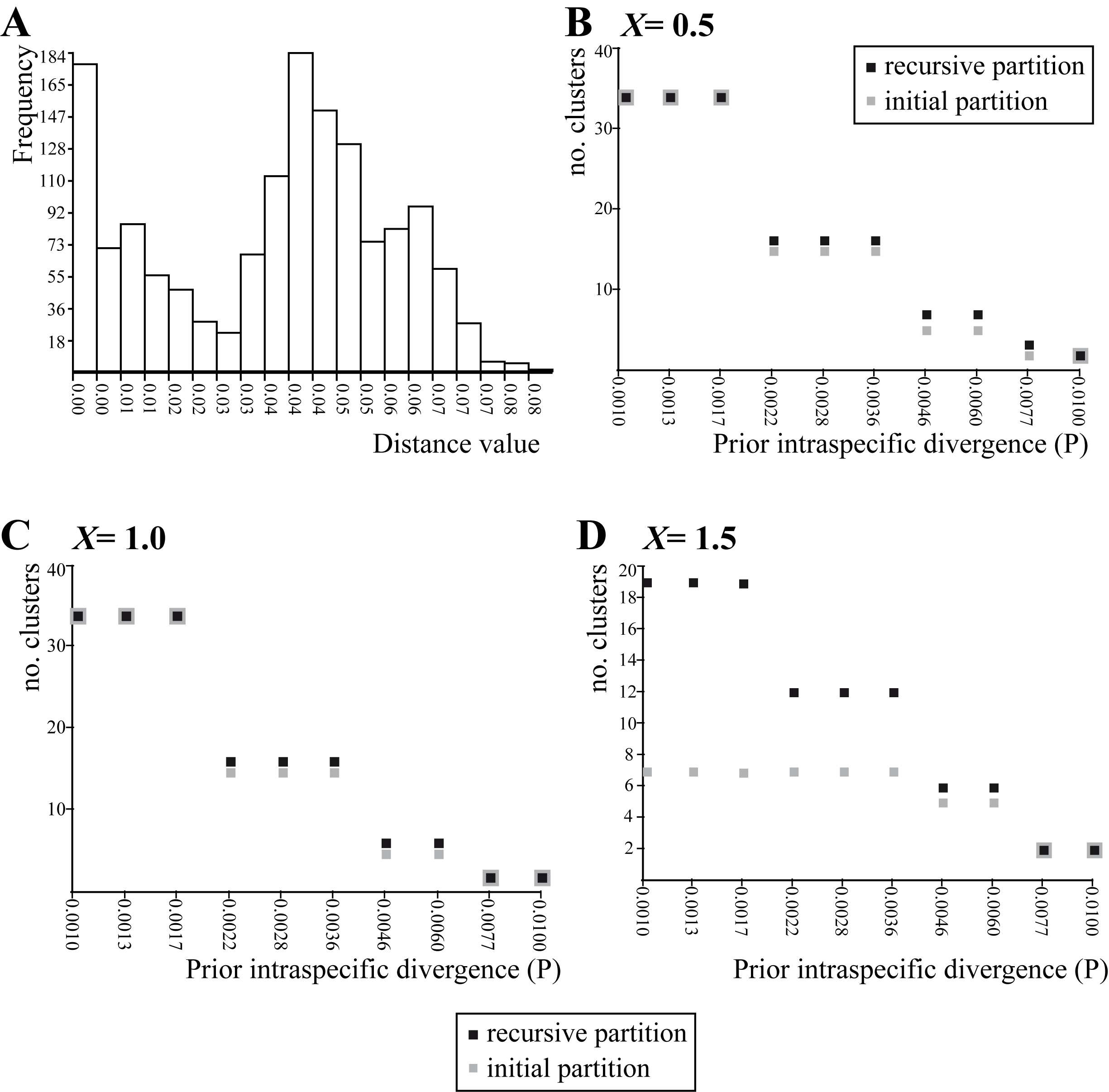

Supplement: Supplementary file 14 — Additional file 14: Figure S5. ABGD results for species delimitation in Sarea. A Histogram showing the distribution of pairwise genetic distances (K2P) among sequences (specimens). B–D Graphs showing the inferred number of clusters (i.e., ABGD partitions or putative species) with different Prior intraspecific divergence (P) values. Different values for the relative gap width (X) were used: 0.5 (B), 1.0, and 1.5 (C). [file 43008_2021_56_MOESM14_ESM.tif]

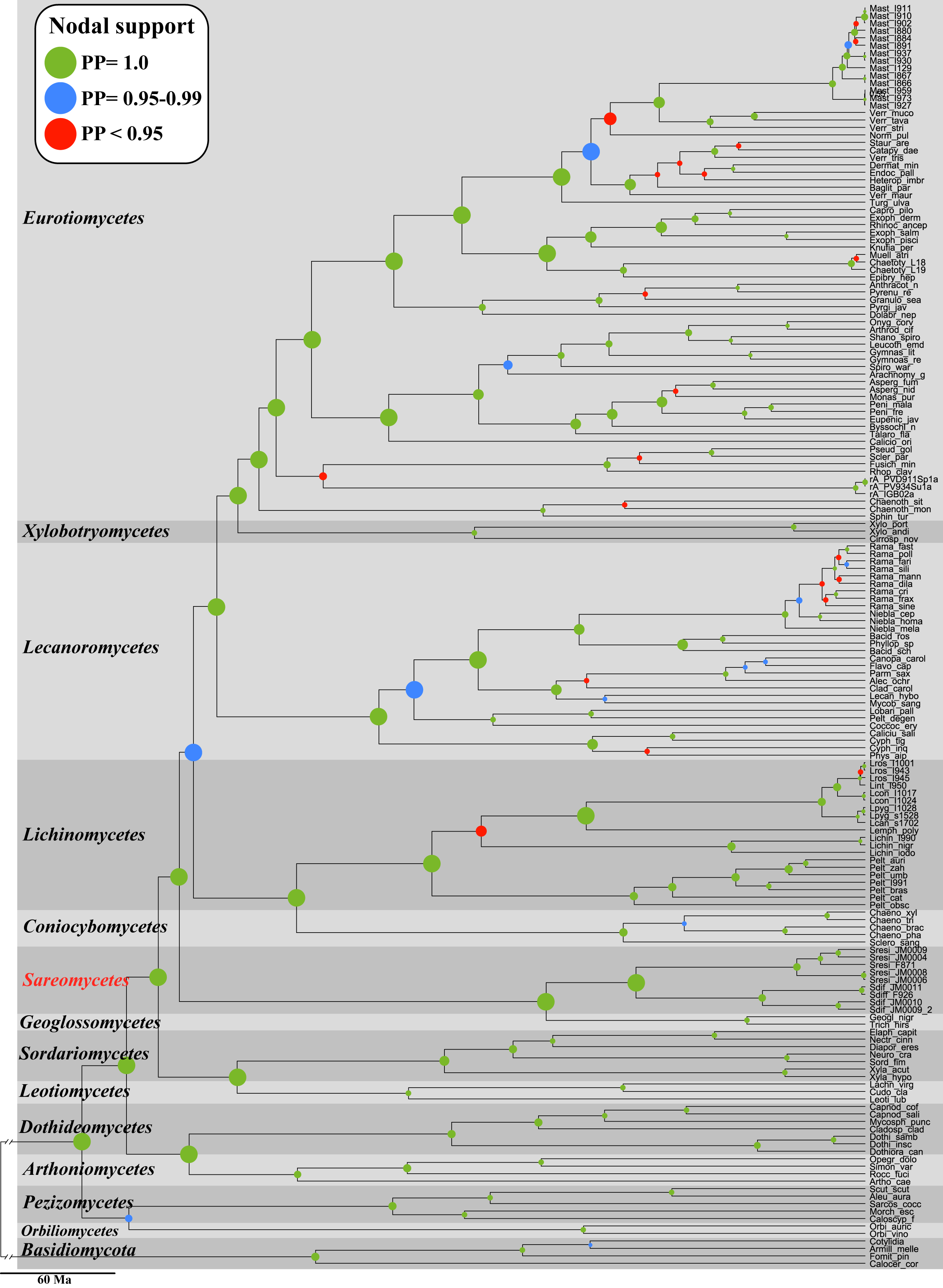

Supplement: Supplementary file 15 — Additional file 15: Figure S6. Six-locus phylogram for Ascomycota with nodal support. Nodal support calculated for the time-calibrated MCC tree constructed in BEAST using a six-locus dataset and 169 fungal taxa, including representatives of the main Ascomycota lineages and Basidiomycota (outgroup). The colour of circles indicates the strength of nodal support (see legend on the upper-left corner); the size of each circle was deliberately chosen to fit the size of the node, and therefore has no associated information. The class Sareomycetes, which represents the focal group of the present study, is highlighted in red. Accession numbers for each marker and considered species are available in Table S3. Ma: million years ago. [file 43008_2021_56_MOESM15_ESM.tif]

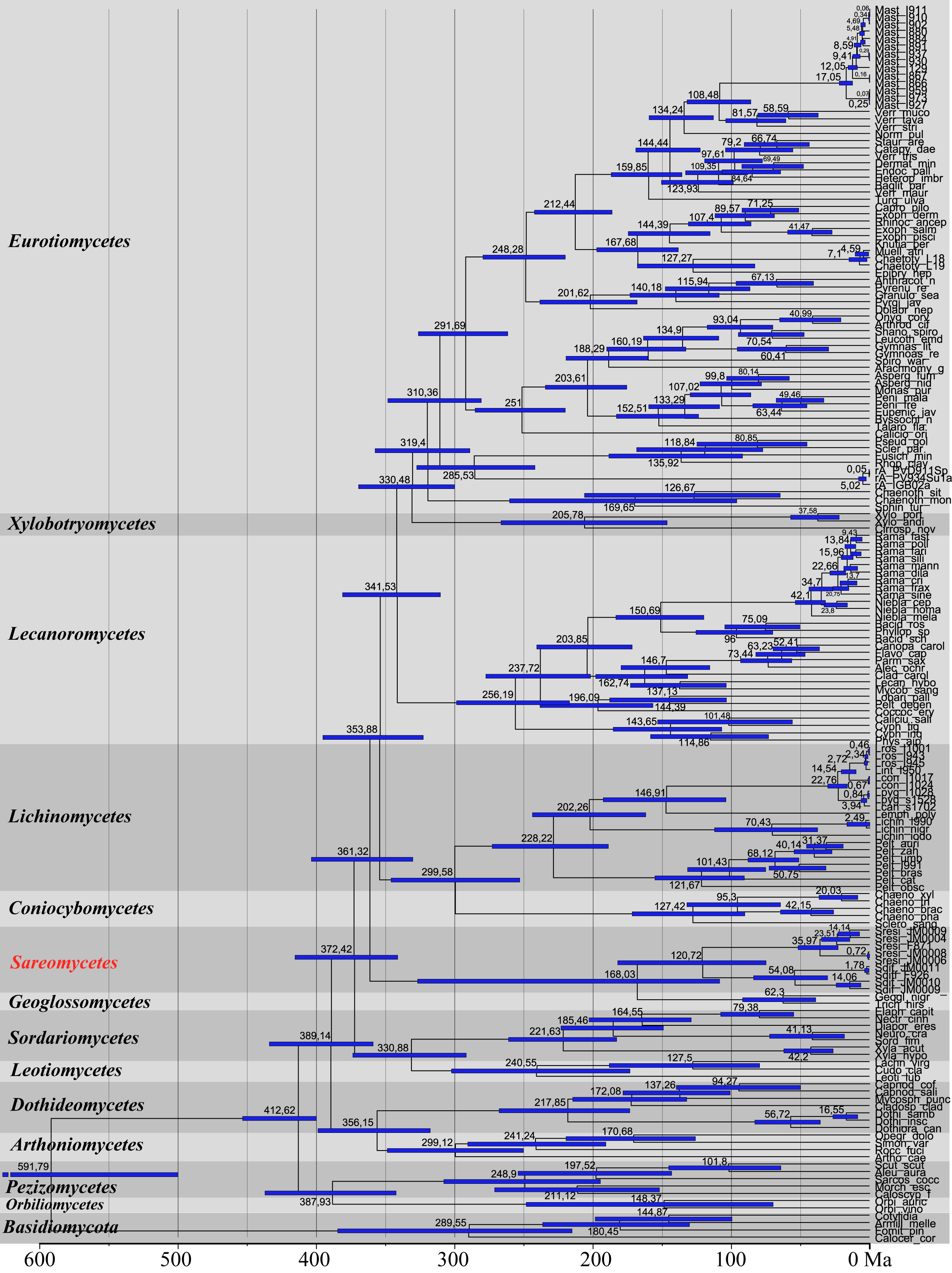

Supplement: Supplementary file 16 — Additional file 16: Figure S7. Six-locus phylogram for Ascomycota with 95% HPD intervals. Nodal 95% Highest Posterior Density (HPD) intervals estimated for divergence ages in the time-calibrated MCC tree constructed in BEAST using a six-locus dataset and 169 fungal taxa, including representatives of the main Ascomycota lineages and Basidiomycota (outgroup). The class Sareomycetes, which represents the focal group of the present study, is highlighted in red. Accession numbers for each marker and considered species are available in Table S3. Ma: million years ago. [file 43008_2021_56_MOESM16_ESM.tif]

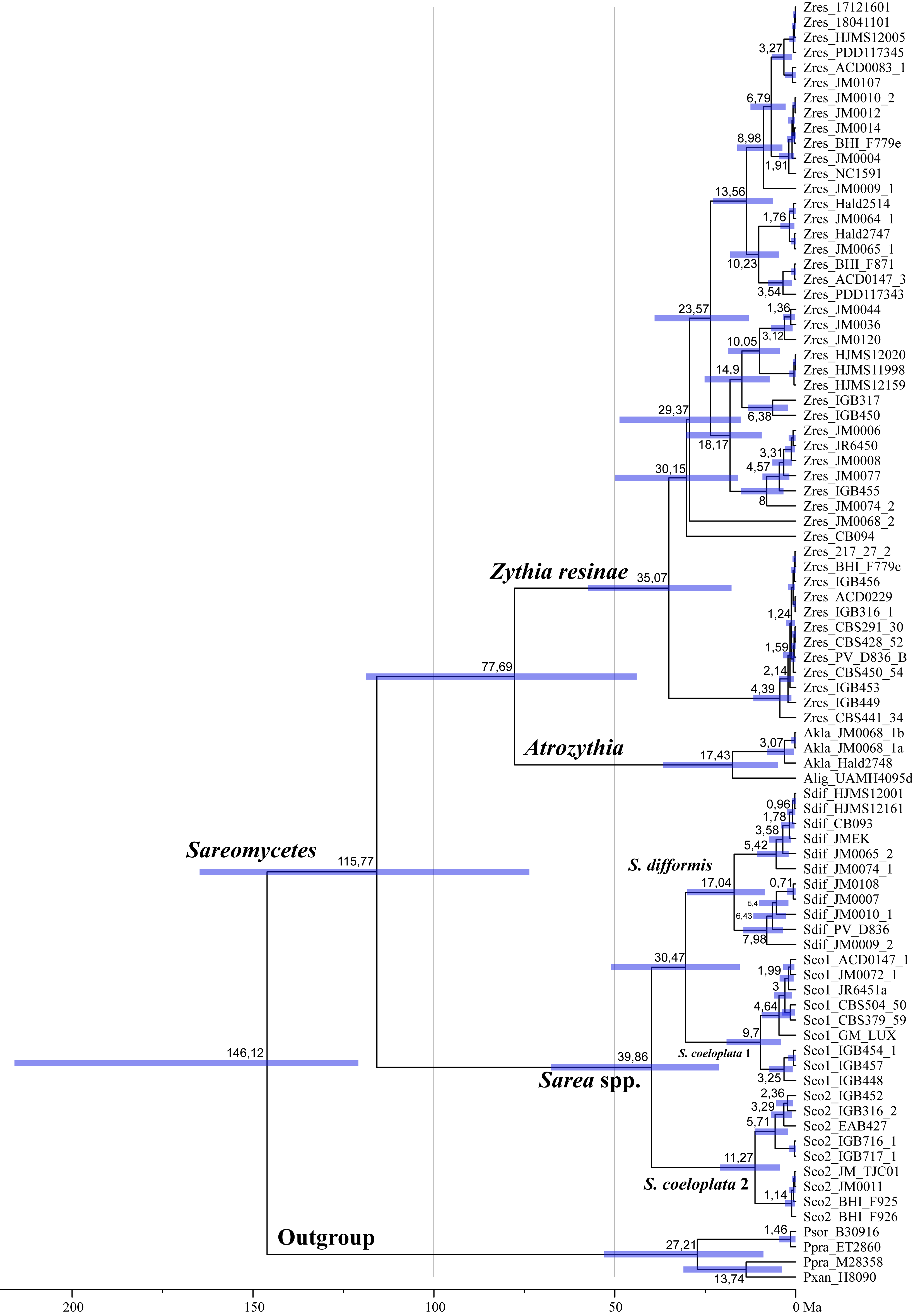

Supplement: Supplementary file 17 — Additional file 17: Figure S8. Three-locus MCC tree calibrated using a date inferred from the six-locus analysis. Time-calibrated MCC tree estimated from a concatenated dataset of ribosomal (nuITS and nuLSU) and mitochondrial (mtSSU) markers from specimens belonging into class Sareomycetes using BEAST. The tree was calibrated imposing a time estimate of 120.88 Ma (181.35–75.76 Ma, 95 % HPD) on the crown node of Sareomycetes based on results of our six-locus dating analysis. Nodal blue bars show the 95% HPD intervals for the estimated divergence ages. The voucher code of each sample is provided. Ma: million years ago. [file 43008_2021_56_MOESM17_ESM.tif]

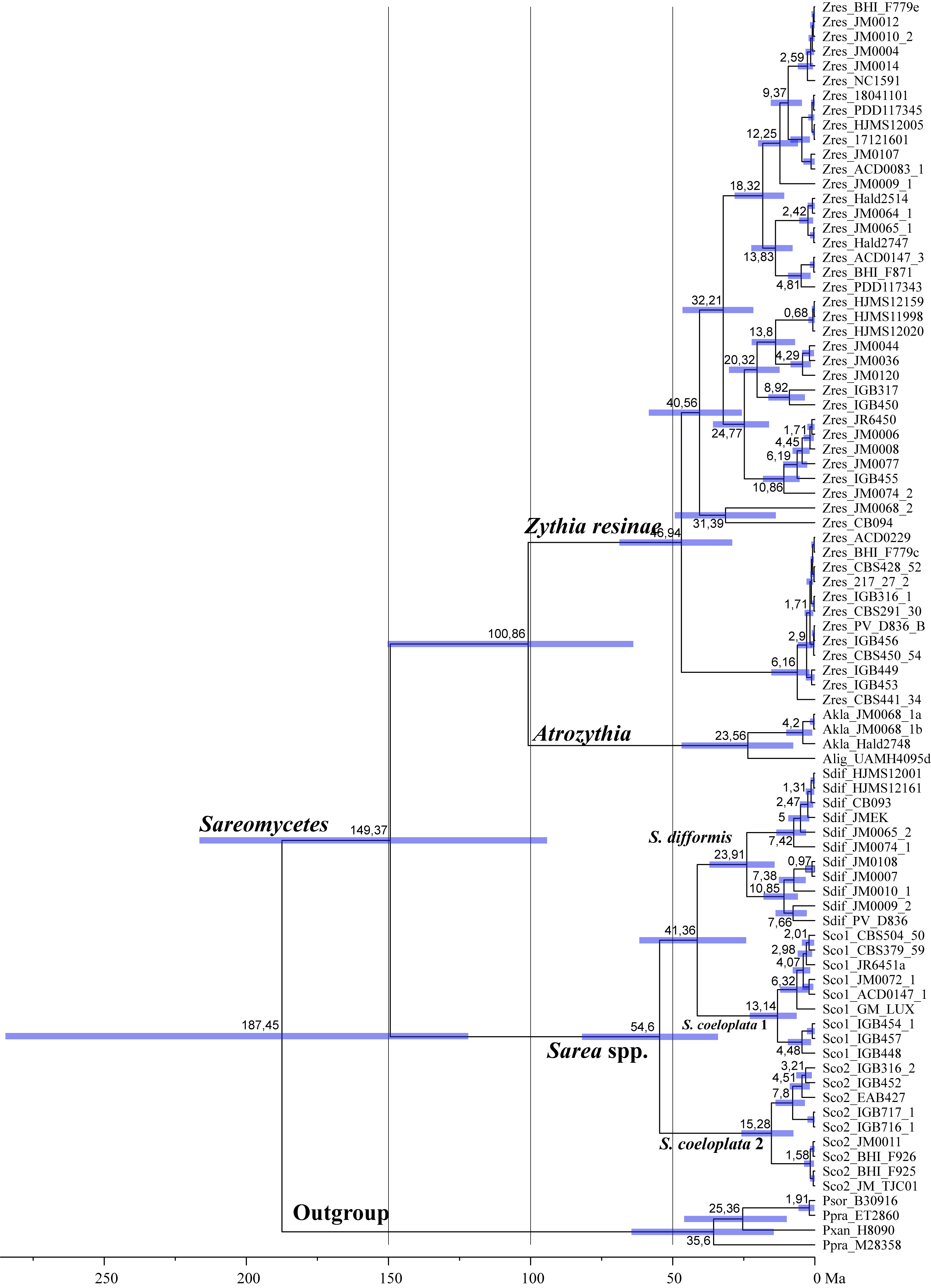

Supplement: Supplementary file 18 — Additional file 18: Figure S9. Three-locus MCC tree calibrated using a mtSSU rate inferred from the six-locus analysis. Time-calibrated MCC tree estimated from a concatenated dataset of ribosomal (nuITS and nuLSU) and mitochondrial (mtSSU) markers from specimens belonging into class Sareomycetes using BEAST. The tree was calibrated imposing a mtSSU rate of 3.28 × 10−10 s/s/y inferred for the Sareomycetes clade in the six-locus dating approach. Nodal blue bars show the 95% HPD intervals for the estimated divergence ages. The voucher code of each sample is provided. Ma: million years ago. [file 43008_2021_56_MOESM18_ESM.tif]

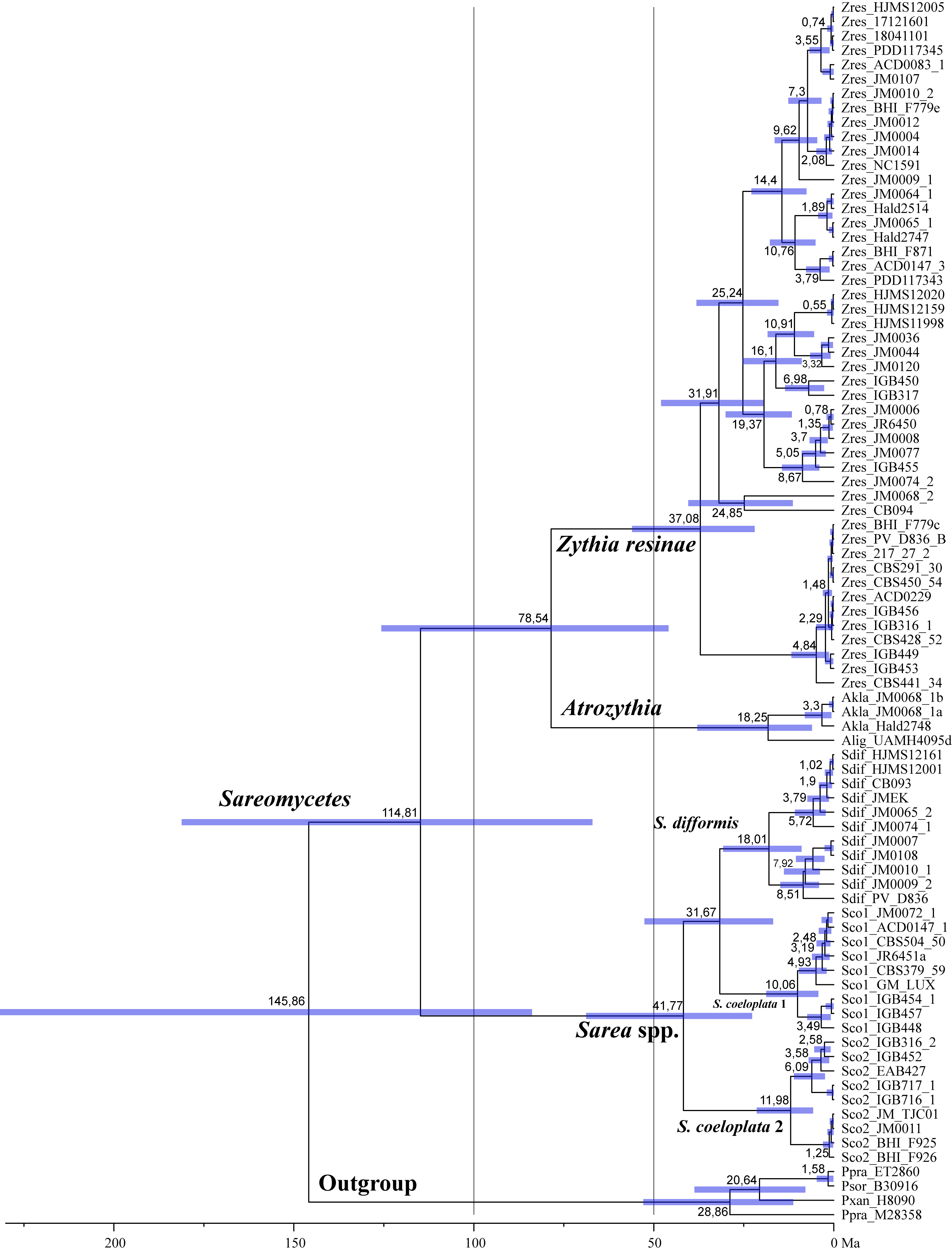

Supplement: Supplementary file 19 — Additional file 19: Figure S10. Three-locus MCC tree calibrated using a nuLSU rate inferred from the six-locus analysis. Time-calibrated MCC tree estimated from a concatenated dataset of ribosomal (nuITS and nuLSU) and mitochondrial (mtSSU) markers from specimens belonging into class Sareomycetes using BEAST. The tree was calibrated imposing a nuLSU rate of 2.68 × 10−10 s/s/y inferred for the Sareomycetes clade in the six-locus dating approach. Nodal blue bars show the 95% HPD intervals for the estimated divergence ages. The voucher code of each sample is provided. Ma: million years ago. [file 43008_2021_56_MOESM19_ESM.tif]

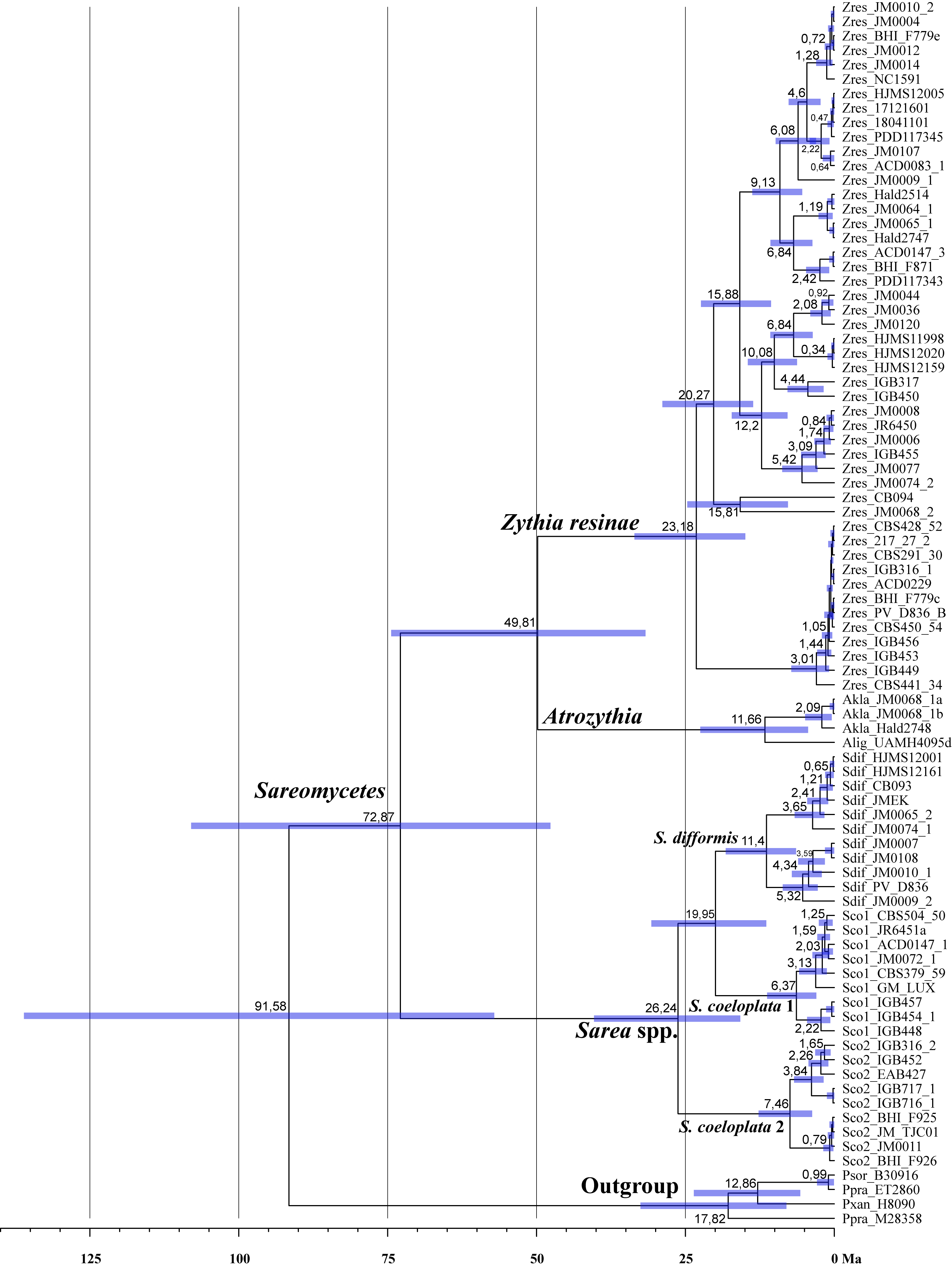

Supplement: Supplementary file 20 — Additional file 20: Figure S11. Three-locus MCC tree calibrated using a nuITS rate estimated for Erysiphales. Time-calibrated MCC tree estimated from a concatenated dataset of ribosomal (nuITS and nuLSU) and mitochondrial (mtSSU) markers from specimens belonging into class Sareomycetes using BEAST. The tree was calibrated imposing a nuITS rate of 2.52 × 10−9 s/s/y calculated for the fungal order Erysiphales by Takamatsu and Matsuda (2004). Nodal blue bars show the 95% HPD intervals for the estimated divergence ages. The voucher code of each sample is provided. Ma: million years ago. [file 43008_2021_56_MOESM20_ESM.tif]

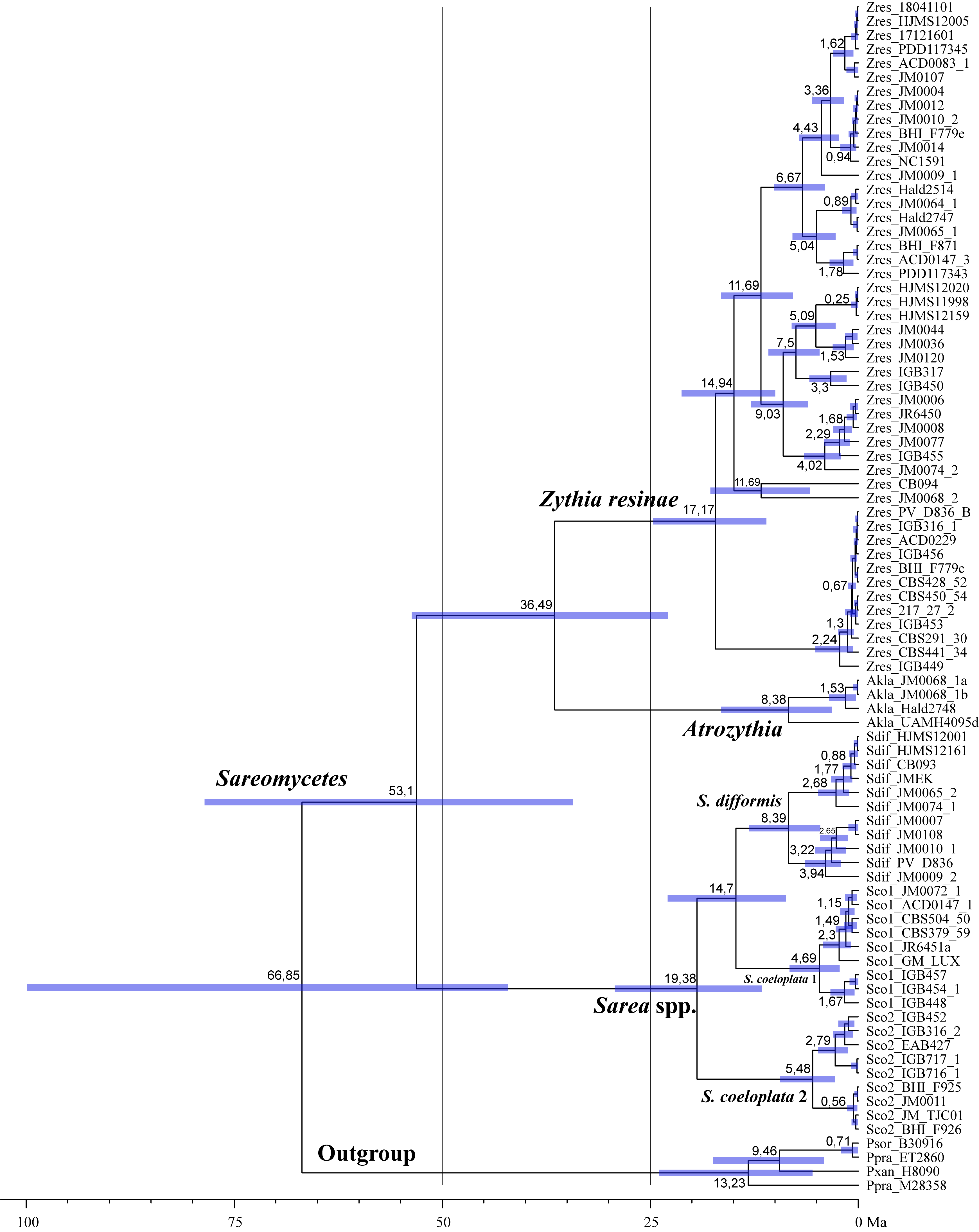

Supplement: Supplementary file 21 — Additional file 21: Figure S12. Three-locus MCC tree calibrated using a nuITS rate estimated for Melanohalea. Time-calibrated MCC tree estimated from a concatenated dataset of ribosomal (nuITS and nuLSU) and mitochondrial (mtSSU) markers from specimens belonging into class Sareomycetes using BEAST. The tree was calibrated imposing a nuITS rate of 3.41 × 10−9 s/s/y calculated for the lichenised fungal genus Melanohalea by Leavitt et al. (2012). Nodal blue bars show the 95% HPD intervals for the estimated divergence ages. The voucher code of each sample is provided. Ma: million years ago. [file 43008_2021_56_MOESM21_ESM.tif]
